# Synthesis of Pyridin-1(2*H*)-ylacrylates and the Effects of Different Functional Groups on Their Fluorescence

**DOI:** 10.3390/molecules28186511

**Published:** 2023-09-08

**Authors:** Weiguang Yang, Danyang Luo, Guanrong Li, Qiaoli Luo, Martin G. Banwell, Lanmei Chen

**Affiliations:** 1 Key Laboratory of Big Data Mining and Precision Drug Design of Guangdong Medical University, The Marine Biomedical Research Institute, Guangdong Medical University, Zhanjiang 524023, China; huluodanyang@163.com (D.L.); lgr971005@163.com (G.L.); 2 School of Chemistry and Chemical Engineering, Lingnan Normal University, Zhanjiang 524048, China; luoql@lingnan.edu.cn; 3 Institute for Advanced and Applied Chemical Synthesis (IAACS), Jinan University, Guangzhou 510632, China

**Keywords:** 2-Aminopyridines, fluorescence, functional groups, pyridin-1(2*H*)-ylacrylates, sulfonyl azides, terminal ynones

## Abstract

While fluorescent organic materials have many potential as well as proven applications and so have attracted significant attention, pyridine–olefin conjugates remain a less studied subset of such systems. Herein, therefore, we report on the development of the straightforward syntheses of pyridin-1(2*H*)-ylacrylates and the outcomes of a study of the effects of substituents on their fluorescent properties. Such compounds were prepared using a simple, metal-free and three-component coupling reaction involving 2-aminopyridines, sulfonyl azides and propiolates. The fluorescent properties of the ensuing products are significantly affected by the positions of substituents on the cyclic framework, with those located in central positions having the greatest impact. Electron-withdrawing groups tend to induce blue shifts while electron-donating ones cause red shifts. This work highlights the capacity that the micro-modification of fluorescent materials provides for fine-tuning their properties such that they may be usefully applied to, for example, the study of luminescent materials.

## 1. Introduction

**Figure 1:**
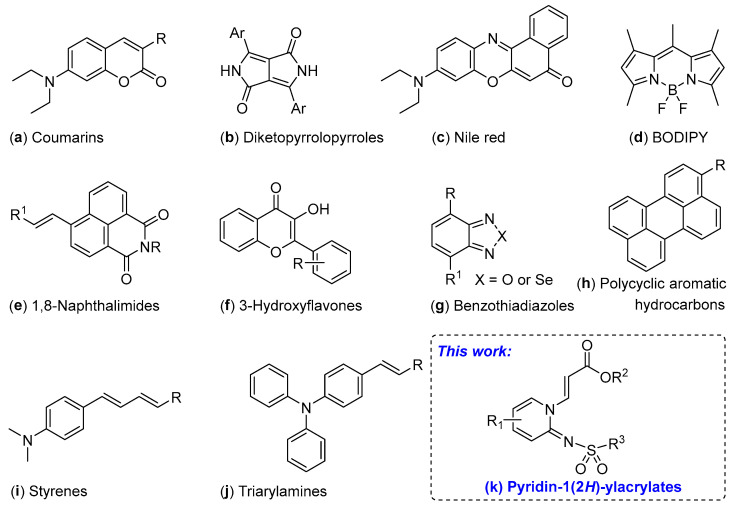
Structures of some key organic fluorescing agents.

Luminescent materials have attracted intense interest owing to their applications in, for example, light-emitting diodes (LEDs), solar cells and laser dyes, as well as their use as photosensitizers, biomolecular labels and molecular probes [1,2,3,4,5,6]. More particularly, organic materials have been widely exploited for their electroluminescence properties because of their simple molecular structures, often ready accessibility and their excellent optical and electrical properties [7], as evidenced by their exploitation in solid lasers [8], field-effect transistors [9], fluorescence probes and biomarkers [10,11,12,13]. Examples of compounds that have been of great value in developing detailed understandings of photophysical processes [14,15,16] are, as shown in Figure 1, coumarin derivatives (**a**) [17,18], diketopyrrolopyrroles (**b**) [19], Nile red-type dyes (**c**) [20], BODIPYs (**d**) [21,22], 1,8-naphthalimides (**e**) [23,24], 3-hydroxyflavones (**f**) [25], benzothiadiazoles (**g**) [26,27], polycyclic aromatic hydrocarbons (**h**) [28], styrenes (**i**) [29,30,31] and triarylamines (**j**) [32,33,34,35,36,37,38]. Although these compounds display good luminous properties, the often-multi-ring structures and attendant structural rigidities together with the complexities of their synthesis and modification can detract from their use. As such, it remains important to identify and prepare new organic fluorescent materials possessing distinctive structural features and photoelectric properties.

Linking pyridines with other π-conjugated motifs can provide a simple and efficient means for preparing non-multi-ring-fused fluorescent materials [39,40,41]. While examples of such systems include imidazo-pyridines [42,43] and pyrrolo [3,4-*c*]pyridines [44], as well as coumarin–pyridine [45] and pyridine–triazole–coumarin conjugates [46], only a few embody pyridine/open-π-chain constructs. Cyanopyridines [47,48] are perhaps the most conspicuous examples of these and are known to be excellent fluorescence agents with the photophysical properties of such conjugated systems able to be fine-tuned through the introduction of different substituents on the heterocyclic ring (and that are thought to, inter alia, affect crystal packing and the associated band gaps).

Based on the foregoing, we sought to prepare pyridin-1(2*H*)-ylacrylates (Figure 1, **k**) in the expectation that these should be both readily accessible and display useful fluorescent properties. In the event, and as detailed below, an efficient, one-pot and three-component coupling process did indeed deliver such compounds. Furthermore, the fluorescent properties of these could be tuned through variations in the substituents attached to the “parent” framework.

## 2. Results and Discussion

### 2.1. Synthesis of Pyridin-1(2H)-ylacrylate Derivatives

Informed by earlier studies [49,50], our present investigations began with efforts to prepare ethyl (*E*)-3-((*E*)-2-(tosylimino)pyridin-1(2*H*)-yl)acrylate (**4a**) via coupling of 2-aminopyridine (**1a**,), ethyl propiolate (**2a**) and *p-*tosyl azide (**3a**)—see Table 1. The initial reactions were carried out at 60 °C in the presence of ferrocene [Fe(C_5_H_5_)_2_] and using DCE as solvent. After 12 h, and following chromatographic purification, target **4a** was obtained in a 68% yield (Table 1, Entry 1). In efforts to improve this outcome, various other catalysts were screened and so revealed that both ferrous- and ferric-based-catalysts were effective (Table 1, Entries 2−4) while Pd-, Rh- and Cu-based ones were not (Table 1, Entries 5–7). Surprisingly, the reaction giving the highest yield of the desired product involved no catalyst at all (Table 1, Entry 8). While comparable yields were obtained using toluene, CH_2_Cl_2_, DCE, THF, 1,4-dioxane, DMSO or DMF as solvents, employing MeCN as the reaction medium gave target **4a** in the highest yield (78%) (Table 1, Entries 9–15). Monitoring the reaction by TLC revealed that it was complete in less than 12 h at 60 °C (Table 1, Entries 16–23).

**Scheme 1:**
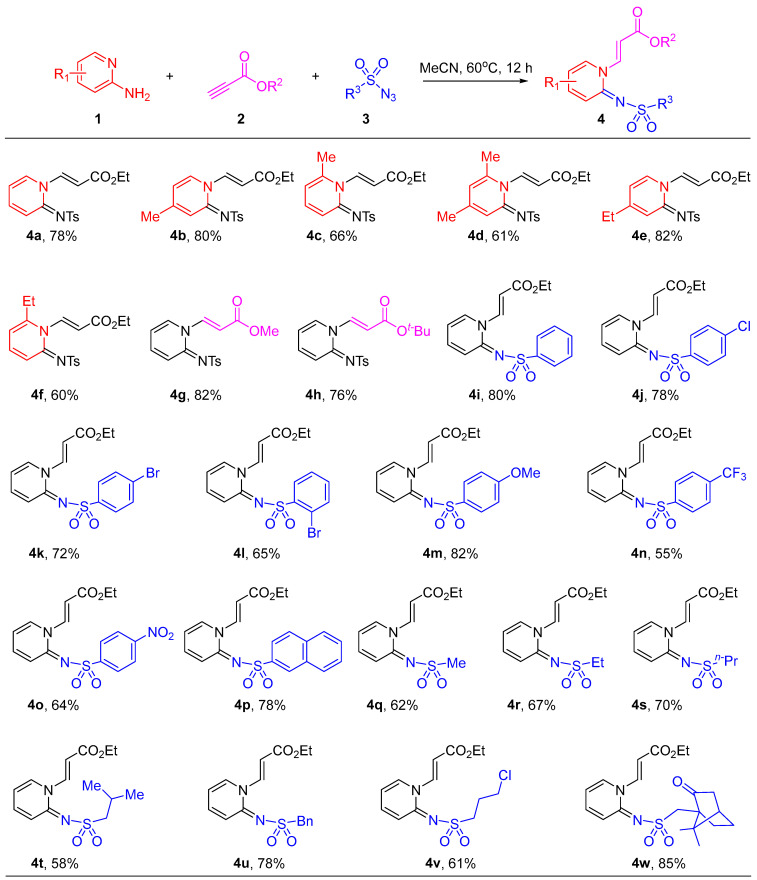
The synthesis of the three-component coupling products **4a**–**4w**.

With optimized reaction conditions defined, substrate variations were next examined by employing a range of sulfonyl 2-aminopyridines (**1**) in the first instance. As shown in Scheme 1, the anticipated products were formed when alkyl groups, including Me or Et, were attached to the pyridine nucleus and so delivered compounds **4a**–**4f** in yields ranging from 60% to 78% and with substituents at C6 resulting in slightly poorer outcomes (see products **4c**, **4d** and **4f**), presumably as a result of adverse steric effects. When either electron-withdrawing (–CN or halogens) or strongly electron-donating (–OH, –NH_2_ or –OMe) groups were present in the substrate, then the desired products were not obtained. The only other propiolates investigated as substrates were the methyl and *tert*-butyl congeners and these gave the anticipated products (e.g., **4g** and **4h**) in good yield. In terms of sulfonyl azides (**3**) serving as substrates, when these carried either aliphatic or aromatic substituents including Me, Et, *^n–^*Pr, benzyl, phenyl or even strongly electron-donating and electron-withdrawing groups (e.g., 4–OMeC_6_H_4_, 4–NO_2_C_6_H_4_ and 4–CF_3_C_6_H_4_), then the anticipated products (**4i**–**4w**) were all obtained in acceptable yields with the camphor sulfonyl azide affording the corresponding product, *viz.* **4w**, in the highest yield (85%).

**Figure 2:**
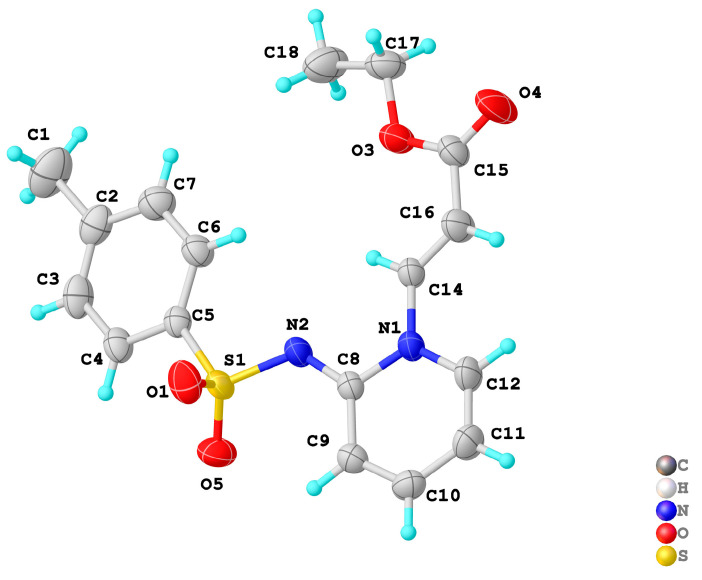
Plot derived from the single-crystal X-ray analysis of product **4a**.

The structure of the “parent” product **4a** was confirmed by single-crystal X-ray analysis (Figure 2, CCDC deposition number 2279385).

### 2.2. Optical Characterization of Pyridin-1(2H)-ylacrylate Derivatives

**Figure 3:**
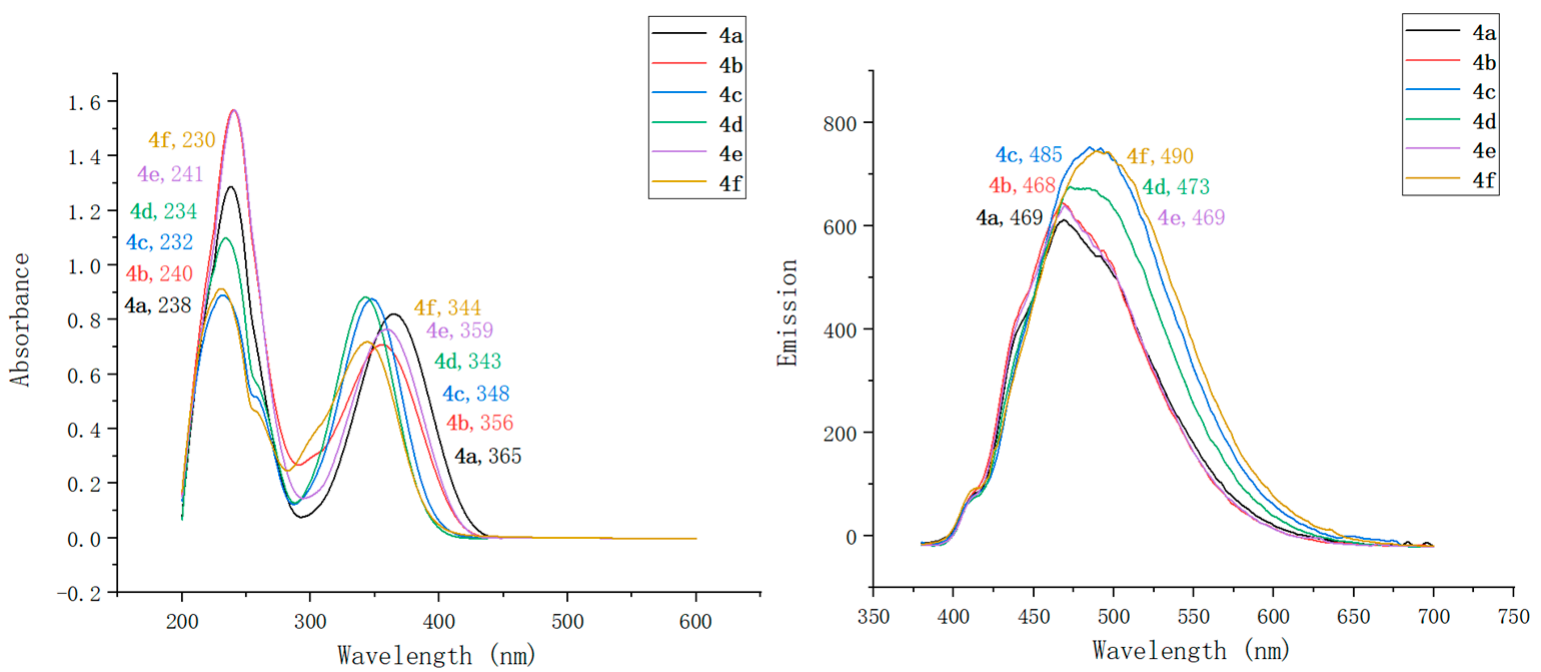
UV–vis absorption (**left**) and fluorescence (**right**) spectra of compounds **4a**–**4f** (spectra recorded in MeOH).

**Figure 4:**
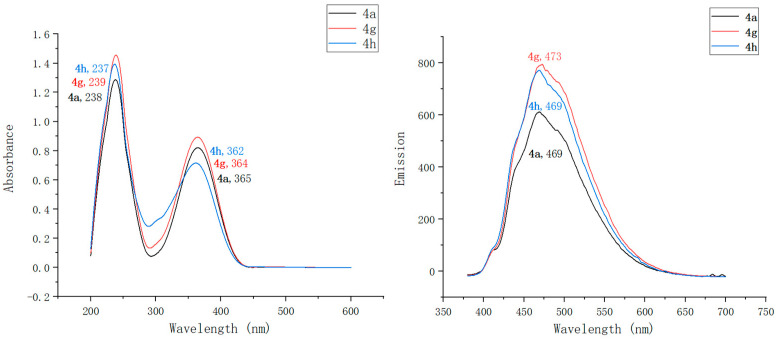
UV–vis absorption (**left**) and fluorescence (**right**) spectra of compounds **4a**, **4g**–**4h** (spectra recorded in MeOH).

**Figure 5:**
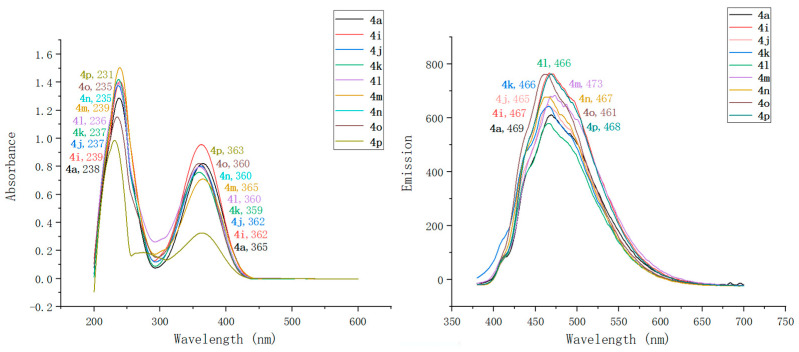
UV–vis absorption (**left**) and fluorescence (**right**) spectra of compounds **4a**, **4i**–**4p** (spectra recorded in MeOH).

**Figure 6:**
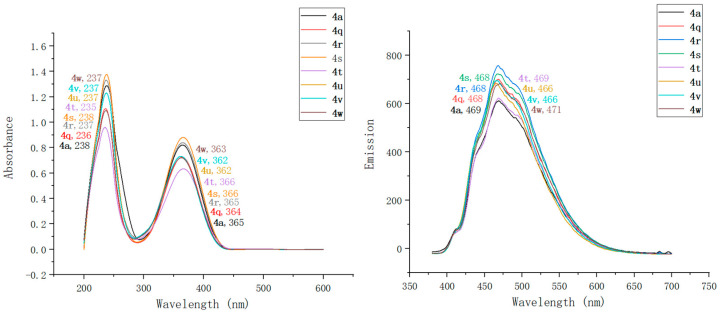
UV–vis absorption (**left**) and fluorescence (**right**) spectra of compounds **4a**, **4q**–**4w** (spectra recorded in MeOH).

Investigations of the photophysical properties of products **4a**–**w** involved the recording of their UV–vis absorption and fluorescence spectra and using methanol as solvent. The outcomes of such studies are shown in Table 2 and Figure 3, Figure 4, Figure 5 and Figure 6.

As solutions in MeOH, most of these derivatives display two conspicuous absorption bands in the region from 231 to 366 nm and fluorescence bands in the range from 466 to 490 nm. Furthermore, and as can be discerned from Figure 3, the fluorescence properties of the parent framework are greatly affected by the positions of substituents, with those located in the “middle” of this framework having the greatest impact (and causing a red shift). Substituents located toward either end of the framework result in much-diminished effects. For example, the 4-methyl-substituted compound **4b** emits at 468 nm, its 6-methyl-substituted counterpart **4c** at 485 nm, and with the 6-ethyl-substituted system **4f** having the longest wavelength emission band (centered at 490 nm).

In contrast to the forgoing, when the alcohol residue associated with the propargyl ester moiety is varied the characteristics of these systems (*viz.* in compounds **4g**–**4h**) do not change (Figure 4), save for the intensities of their fluorescence bands being stronger than those of the “parent” **4a**.

As can be discerned from Figure 5 and Figure 6, the presence of different electron-donating and electron-withdrawing groups attached to the sulfonyl residues of the pyridin-1(2*H*)-ylacrylate framework also has some influence on photophysical properties. So, while the parent framework **4a** displays a fluorescence band at 469 nm, those derivatives bearing electron-withdrawing groups, *viz.*, compounds **4n** and **4o**, show modest blue shifts of 467 and 461 nm, respectively. In contrast, that bearing an electron-donating group (**4m**) shows a red shift to 473 nm. More significantly, in solution, these compounds show (large) Stokes shifts of between 101 and 146 nm (Table 2), features that mean the emission band can be detected without interference from the excitation event and so allowing for the clarity of measurement (*viz*. reabsorption effects are avoided). Furthermore, such an adsorption/emission profile means there should be limited interference with the background fluorescence of any biomolecules under investigation [51] if the compounds are to be used in, for example, bioimaging experiments. The fluorescence efficiency (*Φ*_F_) of the title compounds, as determined by standard methods, is in the range between 0.04 and 0.72 [52]. Given this and the previously mentioned absorption and emission data, compound **4m**, which contains the electron-donating methoxy group, would appear to have a particularly favorable photophysical profile with an absorption wavelength of 365 nm, an emission wavelength of 443 nm and the highest *Φ*_F_ value (0.72).

## 3. Materials and Methods

### 3.1. General Methods

Melting points were determined on a Yanaco melting point apparatus (Kyoto, Japan) and are uncorrected. IR spectra were recorded as KBr pellets on a Nicolet FT-IR 5DX spectrometer (Waltham, MA, USA). All ^1^H NMR (400 MHz) and ^13^C NMR (100 MHz) spectra were recorded on a Bruker AVANCE NEO 400 MHz instrument (Berne, Switzerland) and, unless otherwise indicated, using DMSO-*d*_6_ or CDCl_3_ as solvent. (CH_3_)_4_Si was used as the internal reference while *J* values are given in Hz. HRMS were obtained on a Thermo Scientific Q Exactive Focus Orbitrap LC-MS/MS spectrometer (Waltham, MA, USA). 2-Aminopyridines of the general form **1** and propargyl esters of the general form **2** were obtained commercially while sulfonyl azides were prepared using established methods [53].

### 3.2. General Procedure for the Synthesis of Pyridin-1(2H)-ylacrylates

Solutions of the relevant 2-aminopyridine **1** (1.0 mmol), ester **2** (2.0 mmol) and sulfonyl azide **3** (1.5 mmol) in MeCN (3.0 mL) were added to an oven-dried Schlenk tube equipped with a magnetic stirring bar. Thereafter, the solution was stirred at 60 °C for 12 h before being cooled and then concentrated under reduced pressure. The residue was subjected to flash column chromatography (silica gel, 15% EtOAc in petroleum ether) to afford, after concentration of the relevant fractions, the corresponding pyridin-1(2*H*)-ylacrylate **4**.

*Ethyl (E)-3-((E)-2-(tosylimino)pyridin-1(2H)-yl)acrylate* (**4a**), 135.0 mg (78%), yellow solid, mp 120–121 °C (*R*_f_ = 0.4 in 3:2 *v/v* ethyl acetate/60–90 petroleum ether). IR *ν* 1715, 1638, 1493, 1369, 1267, 1169, 1136, 1080, 961, 758, 733, 683, 660 cm^−1^; ^1^H NMR (400 MHz, CDCl_3_) *δ* 8.47 (d, *J* = 14.4 Hz, 1H), 7.88 (d, *J* = 8.0 Hz, 2H), 7.81 (d, *J* = 9.6 Hz, 1H), 7.58 (d, *J* = 6.0 Hz, 1H), 7.49 (t, *J* = 8.8 Hz, 1H), 7.27 (d, *J* = 4.8 Hz, 2H), 6.53 (t, *J* = 8.2 Hz, 1H), 6.08 (d, *J* = 14.4 Hz, 1H), 4.24–4.29 (m, 2H), 2.40 (s, 3H), 1.32 (t, *J* = 7.4 Hz, 3H); ^13^C NMR (100 MHz, CDCl_3_) *δ* 164.8, 155.1, 142.5, 140.7, 140.5, 140.3, 133.6, 129.4 (2C), 126.5 (2C), 119.3, 114.3, 111.1, 61.4, 21.6, 14.3; HRMS (ESI-TOF) (*m/z*). Calcd for C_17_H_19_N_2_O_4_S^+^, [M+H]^+^ 347.1060; Found 347.1059.

*Ethyl (E)-3-((E)-4-methyl-2-(tosylimino)pyridin-1(2H)-yl)acrylate* (**4b**). 144.0 mg (80%), yellow solid, mp 158–159 °C (*R*_f_ = 0.3 in 1:1 *v/v* ethyl acetate/60–90 petroleum ether). IR *ν* 1717, 1639, 1493, 1335, 1269, 1142, 1082, 870, 712, 683, 664 cm^−1^; ^1^H NMR (400 MHz, CDCl_3_) *δ* 8.46 (d, *J* = 14.4 Hz, 1H), 7.86 (d, *J* = 5.2 Hz, 2H), 7.56 (s, 1H), 7.48 (d, *J* = 6.4 Hz, 1H), 7.25 (d, *J* = 8.4 Hz, 2H), 6.37 (d, *J* = 8.4 Hz, 1H), 6.04 (d, *J* = 15.2 Hz, 1H), 4.21-4.27 (m, 2H), 2.39 (s, 3H), 2.29 (s, 3H), 1.30 (t, *J* = 7.6 Hz, 3H); ^13^C NMR (100 MHz, CDCl_3_) *δ* 164.9, 154.6, 153.8, 142.3, 140.5, 140.2, 132.5, 129.3 (2C), 126.5 (2C), 117.6, 113.8, 113.3, 61.3, 22.2, 21.6, 14.3; HRMS (ESI-TOF) (*m/z*). Calcd for C_18_H_21_N_2_O_4_S^+^, [M+H]^+^ 361.1217; Found 361.1217.

*Ethyl (E)-3-((E)-6-methyl-2-(tosylimino)pyridin-1(2H)-yl)acrylate* (**4c**). 118.8 mg (66%), yellow solid, mp 94–95 °C (*R*_f_ = 0.2 in 1:1 *v/v* ethyl acetate/60–90 petroleum ether). IR *ν* 2922, 1719, 1632, 1487, 1375, 1269, 1136, 1092, 827, 731, 660 cm^−1^; ^1^H NMR (400 MHz, CDCl_3_) *δ* 7.83 (d, *J* = 8.4 Hz, 2H), 7.60 (t, *J* = 9.8 Hz, 2H), 7.40 (t, *J* = 8.6 Hz, 1H), 7.22 (d, *J* = 8.0 Hz, 2H), 6.42 (d, *J* = 6.8 Hz, 1H), 6.06 (d, *J* = 11.2 Hz, 1H), 4.25-4.31 (m, 2H), 2.37 (d, *J* = 4.8 Hz, 6H), 1.33 (t, *J* = 7.0 Hz, 3H); ^13^C NMR (100 MHz, CDCl_3_) *δ* 164.3, 157.4, 146.1, 142.0, 140.8, 140.5, 140.1, 129.2 (2C), 126.4 (2C), 123.8, 115.9, 111.9, 61.5, 22.2, 21.6, 14.3; HRMS (ESI-TOF) (*m/z*). Calcd for C_18_H_21_N_2_O_4_S^+^, [M+H]^+^ 361.1217; Found 361.1214.

*Ethyl (E)-3-((E)-4,6-dimethyl-2-(tosylimino)pyridin-1(2H)-yl)acrylate* (**4d**). 114.1 mg (61%), yellowish solid, mp 167–168 °C [*R*_f_ = 0.2(5) in 1:1 *v/v* ethyl acetate/60–90 petroleum ether]. IR *ν* 2922, 1717, 1636, 1481, 1362, 1279, 1138, 1078, 951, 837, 816, 667 cm^−1^; ^1^H NMR (400 MHz, CDCl_3_) *δ* 7.83 (d, *J* = 8.0 Hz, 2H), 7.57 (d, *J* = 14.0 Hz, 1H), 7.41 (s, 1H), 7.22 (d, *J* = 8.8 Hz, 2H), 6.27 (s, 1H), 6.03 (d, *J* = 14.4 Hz, 1H), 4.24-4.29 (m, 2H), 2.38 (s, 3H), 2.32 (s, 3H), 2.24 (s, 3H), 1.32 (t, *J* = 7.6 Hz, 3H); ^13^C NMR (100 MHz, CDCl_3_) *δ* 164.4, 156.8, 153.2, 144.8, 141.9, 141.01, 140.00, 129.2 (2C), 126.4 (2C), 123.4, 114.7, 114.5, 61.4, 22.0, 21.9, 21.6, 14.3; HRMS (ESI-TOF) (*m/z*). Calcd for C_19_H_23_N_2_O_4_S^+^, [M+H]^+^ 375.1373; Found 375.1372.

*Ethyl (E)-3-((E)-4-ethyl-2-(tosylimino)pyridin-1(2H)-yl)acrylate* (**4e**). 153.4 mg (82%), yellowish solid, mp 144–146 °C (*R*_f_ = 0.3 in 2:3 *v/v* ethyl acetate/60–90 petroleum ether). IR *ν* 2924, 1717, 1639, 1489, 1267, 1142, 1082, 966, 858, 733, 704, 683, 664 cm^−1^; ^1^H NMR (400 MHz, CDCl_3_) *δ* 8.48 (d, *J* = 17.6 Hz, 1H), 7.87 (d, *J* = 6.4 Hz, 2H), 7.57 (s, 1H), 7.48 (d, *J* = 10.0 Hz, 1H), 7.25 (d, *J* = 8.8 Hz, 2H), 6.39 (d, *J* = 5.2 Hz, 1H), 6.04 (d, *J* = 11.6 Hz, 1H), 4.22–4.27 (m, 2H), 2.54–2.60 (m, 2H), 2.39 (s, 3H), 1.30 (t, *J* = 6.0 Hz, 3H), 1.21 (t, *J* = 6.2 Hz, 3H); ^13^C NMR (100 MHz, CDCl_3_) *δ* 165.0, 159.2, 154.9, 142.3, 140.6, 140.2, 132.6, 129.3 (2C), 126.5 (2C), 116.3, 113.3, 112.6, 61.3, 29.1, 21.6, 14.3, 13.1; HRMS (ESI-TOF) (*m/z*). Calcd for C_19_H_23_N_2_O_4_S^+^, [M+H]^+^ 375.1373; Found 375.1370.

*Ethyl (E)-3-((E)-6-ethyl-2-(tosylimino)pyridin-1(2H)-yl)acrylate* (**4f**). 112.2 mg (60%), brown oil (*R*_f_ = 0.3 in 1:1 *v/v* ethyl acetate/60–90 petroleum ether). IR *ν* 2922, 1717, 1630, 1487, 1379, 1275, 1136, 1088, 1034, 810, 733, 662 cm^−1^; ^1^H NMR (400 MHz, CDCl_3_) *δ* 7.81 (d, *J* = 8.4 Hz, 2H), 7.57 (t, *J* = 11.6 Hz, 2H), 7.44 (t, *J* = 6.8 Hz, 1H), 7.21 (d, *J* = 5.2 Hz, 2H), 6.43 (d, *J* = 7.6 Hz, 1H), 6.05 (d, *J* = 16.8 Hz, 1H), 4.24–4.30 (m, 2H), 2.62–2.68 (m, 2H), 2.37 (s, 3H), 1.32 (t, *J* = 7.6 Hz, 3H), 1.20 (t, *J* = 5.6 Hz, 3H); ^13^C NMR (100 MHz, CDCl_3_) *δ* 164.2, 157.3, 151.6, 142.0, 140.8, 140.7, 139.8, 129.2 (2C), 126.4 (2C), 123.9, 115.6, 109.7, 61.5, 27.2, 21.5, 14.2, 12.2; HRMS (ESI-TOF) (*m/z*). Calcd for C_19_H_23_N_2_O_4_S^+^, [M+H]^+^ 375.1373; Found 375.1372.

*Methyl (E)-3-((E)-2-(tosylimino)pyridin-1(2H)-yl)acrylate* (**4g**). 136.2 mg (82%), brown solid, mp 128–130 °C [*R*_f_ = 0.4(5) in 2:1 *v/v* ethyl acetate/60–90 petroleum ether]. IR *ν* 2922, 1717, 1636, 1493, 1375, 1269, 1167, 1136, 1080, 961, 758, 683, 662 cm^−1^; ^1^H NMR (400 MHz, CDCl_3_) *δ* 8.49 (d, *J* = 17.2 Hz, 1H), 7.87 (d, *J* = 10.8 Hz, 2H), 7.80 (d, *J* = 9.2 Hz,1H), 7.57 (d, *J* = 9.2 Hz, 1H), 7.49 (t, *J* = 7.8 Hz, 1H), 7.27 (d, *J* = 7.2 Hz, 2H), 6.53 (t, *J* = 7.6 Hz, 1H), 6.10 (d, *J* = 11.6 Hz, 1H), 3.81 (s, 3H), 2.40 (s, 3H); ^13^C NMR (100 MHz, CDCl_3_) *δ* 165.2, 155.2, 142.6, 140.74, 140.69, 140.2, 133.6, 129.4 (2C), 126.6 (2C), 119.3, 113.9, 111.1, 52.4, 21.6; HRMS (ESI-TOF) (*m/z*). Calcd for C_16_H_17_N_2_O_4_S^+^, [M+H]^+^ 333.0904; Found 333.0903.

*Tert-butyl (E)-3-((E)-2-(tosylimino)pyridin-1(2H)-yl)acrylate* (**4h**). 142.2 mg (76%), brown oil [*R*_f_ = 0.4(5) in 6:5 *v/v* ethyl acetate/60–90 petroleum ether]. IR *ν* 2924, 1709, 1638, 1495, 1369, 1271, 1134, 1080, 961, 754, 685, 662 cm^−1^; ^1^H NMR (400 MHz, CDCl_3_) *δ* 8.36 (d, *J* = 11.6 Hz, 1H), 7.87 (d, *J* = 5.6 Hz, 2H), 7.80 (d, *J* = 9.2 Hz, 1H), 7.56 (d, *J* = 7.2 Hz, 1H), 7.48 (t, *J* = 9.2 Hz, 1H), 7.25 (d, *J* = 6.8 Hz, 2H), 6.52 (t, *J* = 7.4 Hz, 1H), 6.00 (d, *J* = 14.4 Hz, 1H), 2.39 (s, 3H), 1.49 (s, 9H); ^13^C NMR (100 MHz, CDCl_3_) *δ* 163.9, 155.0, 142.4, 140.6, 140.5, 139.7, 133.7, 129.4 (2C), 126.5 (2C), 119.4, 116.1, 111.0, 82.1, 28.2 (3C), 21.6; HRMS (ESI-TOF) (*m/z*). Calcd for C_19_H_23_N_2_O_4_S^+^, [M+H]^+^ 375.1373; Found 375.1372.

*Ethyl (E)-3-((Z)-2-((phenylsulfonyl)imino)pyridin-1(2H)-yl)acrylate* (**4i**). 132.8 mg (80%), brown oil [*R*_f_ = 0.2(5) in 1:1 *v/v* ethyl acetate/60–90 petroleum ether]. IR *ν* 1717, 1636, 1493, 1369, 1269, 1171, 1138, 1082, 1038, 961, 754, 691 cm^−1^; ^1^H NMR (400 MHz, CDCl_3_) *δ* 8.47 (d, *J* = 14.8 Hz, 1H), 7.99 (d, *J* = 6.0 Hz, 2H), 7.80 (d, *J* = 9.6 Hz, 1H), 7.59 (d, *J* = 7.68 Hz, 1H), 7.45–7.52 (m, 4H), 6.55 (t, *J* = 6.8 Hz, 1H), 6.10 (d, *J* = 14.8 Hz, 1H), 4.23–4.28 (m, 2H), 1.31 (t, *J* = 6.6 Hz, 3H); ^13^C NMR (100 MHz, CDCl_3_) *δ* 164.7, 155.2, 143.1, 140.9, 140.4, 133.8, 131.9, 128.8 (2C), 126.5 (2C), 119.3, 114.5, 111.2, 61.4, 14.3; HRMS (ESI-TOF) (*m/z*). Calcd for C_16_H_17_N_2_O_4_S^+^, [M+H]^+^ 333.0904; Found 333.0903.

*Ethyl (E)-3-((Z)-2-(((4-chlorophenyl)sulfonyl)imino)pyridin-1(2H)-yl)acrylate* (**4j**). 142.8 mg (78%), brown solid, 143–144 °C [*R*_f_ = 0.2(5) in 1:1 *v/v* ethyl acetate/60–90 petroleum ether]. IR *ν* 2922, 1717, 1636, 1493, 1371, 1269, 1171, 1138, 1082, 1040, 962, 762, 735, 660 cm^−1^; ^1^H NMR (400 MHz, CDCl_3_) *δ* 8.42 (d, *J* = 14.4 Hz, 1H), 7.91 (d, *J* = 9.2 Hz, 2H), 7.77 (d, *J* = 9.6 Hz, 1H), 7.62 (d, *J* = 6.4 Hz, 1H), 7.54 (t, *J* = 7.8 Hz, 1H), 7.43 (d, *J* = 9.2 Hz, 2H), 6.59 (t, *J* = 6.4 Hz, 1H), 6.11 (d, *J* = 14.4 Hz, 1H), 4.23–4.28 (m, 2H), 1.31 (t, *J* = 7.4 Hz, 3H); ^13^C NMR (100 MHz, CDCl_3_) *δ* 164.6, 155.1, 141.7, 141.2, 140.3, 138.2, 133.9, 129.0 (2C), 128.0 (2C), 119.2, 114.8, 111.5, 61.5, 14.3; HRMS (ESI-TOF) (*m/z*). Calcd for C_16_H_16_ClN_2_O_4_S^+^, [M+H]^+^ 367.0514; Found 367.0513.

*Ethyl (E)-3-((Z)-2-(((4-bromophenyl)sulfonyl)imino)pyridin-1(2H)-yl)acrylate* (**4k**). 147.6 mg (72%), yellow solid, 162–163 °C (*R*_f_ = 0.3 in 1:1 *v/v* ethyl acetate/60–90 petroleum ether). IR *ν* 2924, 1717, 1638, 1493, 1371, 1267, 1136, 1082, 962, 760, 731, 702 cm^−1^; ^1^H NMR (400 MHz, CDCl_3_) *δ* 8.43 (d, *J* = 14.8 Hz, 1H), 7.85 (d, *J* = 6.0 Hz, 2H), 7.79 (d, *J* = 9.6 Hz, 1H), 7.60 (d, *J* = 5.2 Hz, 3H), 7.54 (t, *J* = 8.4 Hz, 1H), 6.59 (t, *J* = 7.6 Hz, 1H), 6.10 (d, *J* = 12.0 Hz, 1H), 4.24–4.29 (m, 2H), 1.32 (t, *J* = 7.2 Hz, 3H); ^13^C NMR (100 MHz, CDCl_3_) *δ* 164.6, 155.1, 142.3, 141.2, 140.3, 133.9, 132.0 (2C), 128.2 (2C), 126.7, 119.2, 114.8, 111.5, 61.5, 14.3; HRMS (ESI-TOF) (*m/z*). Calcd for C_16_H_16_BrN_2_O_4_S^+^, [M+H]^+^ 411.0009; Found 411.0008.

*Ethyl (E)-3-((Z)-2-(((2-bromophenyl)sulfonyl)imino)pyridin-1(2H)-yl)acrylate* (**4l**). 133.2 mg (65%), yellow solid, mp 122–124 °C (*R*_f_ = 0.3 in 1:1 *v/v* ethyl acetate/60–90 petroleum ether). IR *ν* 1717, 1636, 1506, 1493, 1371, 1269, 1142, 1123, 1094, 1026, 964, 756, 731, 656 cm^−1^; ^1^H NMR (400 MHz, CDCl_3_) *δ* 8.58 (d, *J* = 12.0 Hz, 1H), 8.26 (d, *J* = 8.0 Hz, 1H), 7.89 (d, *J* = 9.6 Hz, 1H), 7.71 (d, *J* = 5.2 Hz, 1H), 7.61 (d, *J* = 7.2 Hz, 1H), 7.54 (t, *J* = 7.2 Hz, 1H), 7.43 (t, *J* = 7.6 Hz, 1H), 7.33 (t, *J* = 7.2 Hz, 1H), 6.58 (t, *J* = 6.8 Hz, 1H), 6.09 (t, *J* = 14.4 Hz, 1H), 4.22–4.27 (m, 2H), 1.30 (t, *J* = 6.4 Hz, 3H); ^13^C NMR (100 MHz, CDCl_3_) *δ* 164.7, 155.1, 142.1, 140.9, 140.8, 135.3, 133.6, 132.9, 129.9, 127.4, 120.8, 119.9, 114.5, 111.5, 61.4, 14.3; HRMS (ESI-TOF) (*m/z*). Calcd for C_16_H_16_BrN_2_O_4_S^+^, [M+H]^+^ 411.0009; Found 411.0009.

*Ethyl (E)-3-((Z)-2-(((4-methoxyphenyl)sulfonyl)imino)pyridin-1(2H)-yl)acrylate* (**4m**). 148.5 mg (82%), brown oil (*R*_f_ = 0.3 in 3:2 *v/v* ethyl acetate/60–90 petroleum ether). IR *ν* 2922, 1715, 1638, 1493, 1371, 1254, 1136, 1082, 1026, 961, 804, 758, 685 cm^−1^; ^1^H NMR (400 MHz, CDCl_3_) *δ* 8.45 (d, *J* = 11.6 Hz, 1H), 7.90 (d, *J* = 8.8 Hz, 2H), 7.77 (d, *J* = 9.2 Hz, 1H), 7.58 (d, *J* = 7.2 Hz, 1H), 7.48 (t, *J* = 9.0 Hz, 1H), 6.93 (d, *J* = 6.0 Hz, 2H), 6.53 (t, *J* = 6.8 Hz, 1H), 6.09 (d, *J* = 14.4 Hz, 1H), 4.22-4.27 (m, 2H), 3.83 (s, 3H), 1.30 (t, *J* = 6.2 Hz, 3H); ^13^C NMR (100 MHz, CDCl_3_) *δ* 164.8, 162.3, 155.0, 140.7, 140.4, 135.2, 133.7, 128.5 (2C), 119.1, 114.3, 113.9 (2C), 111.0, 61.4, 55.6, 14.3; HRMS (ESI-TOF) (*m/z*). Calcd for C_17_H_19_N_2_O_5_S^+^, [M+H]^+^ 363.1009; Found 363.1007.

*Ethyl (E)-3-((Z)-2-(((4-(trifluoromethyl)phenyl)sulfonyl)imino)pyridin-1(2H)-yl)acrylate* (**4n**). 110.0 mg (55%), yellow solid, mp 156–158 °C (*R*_f_ = 0.3 in 1:1 *v/v* ethyl acetate/60–90 petroleum ether). IR *ν* 2922, 1717, 1638, 1504, 1493, 1321, 1271, 1134, 1084, 1061, 964, 762, 735, 710 cm^−1^; ^1^H NMR (400 MHz, CDCl_3_) *δ* 8.43 (d, *J* = 14.0 Hz, 1H), 8.12 (d, *J* = 8.0 Hz, 2H), 7.83 (d, *J* = 9.6 Hz, 1H), 7.74 (d, *J* = 8.0 Hz, 2H), 7.63 (d, *J* = 7.2 Hz, 1H), 7.58 (t, *J* = 8.2 Hz, 1H), 6.62 (t, *J* = 6.4 Hz, 1H), 6.12 (d, *J* = 12.4 Hz, 1H), 4.24-4.29 (m, 2H), 1.31 (t, *J* = 7.0 Hz, 3H); ^13^C NMR (100 MHz, CDCl_3_) *δ* 164.6, 155.2, 146.6, 141.4, 140.3, 134.0, 133.7 (q, *J* = 32.5 Hz, 1C), 127.1 (2C), 126.0 (q, *J* = 3.7 Hz, 2C), 123.6 (q, *J* = 271.0 Hz, 1C), 119.4, 115.1, 111.7, 61.6, 14.3; HRMS (ESI-TOF) (*m/z*). Calcd for C_17_H_16_F_3_N_2_O_4_S^+^, [M+H]^+^ 401.0778; Found 401.0778.

*Ethyl (E)-3-((Z)-2-(((4-nitrophenyl)sulfonyl)imino)pyridin-1(2H)-yl)acrylate* (**4o**). 120.7 mg (64%), yellow solid, mp 118–120 °C (*R*_f_ = 0.3 in 1:1 *v/v* ethyl acetate/60–90 petroleum ether). IR *ν* 2926, 1717, 1638, 1506, 1493, 1348, 1267, 1140, 1084, 966, 854, 731, 687 cm^−1^; ^1^H NMR (400 MHz, CDCl_3_) *δ* 8.38 (d, *J* = 14.4 Hz, 1H), 8.29 (d, *J* = 5.6 Hz, 2H), 8.14 (d, *J* = 5.6 Hz, 2H), 7.79 (d, *J* = 9.2 Hz, 1H), 7.68 (d, *J* = 7.2 Hz, 1H), 7.61 (t, *J* = 8.2 Hz, 1H), 6.66 (t, *J* = 7.6 Hz, 1H), 6.14 (d, *J* = 14.4 Hz, 1H), 4.22-4.27 (m, 2H), 1.28 (t, *J* = 8.8 Hz, 3H); ^13^C NMR (100 MHz, CDCl_3_) *δ* 164.4, 155.0, 149.6, 148.8, 141.8, 140.2, 134.3, 127.7 (2C), 124.1 (2C), 119.0, 115.4, 112.1, 61.6, 14.2; HRMS (ESI-TOF) (*m/z*). Calcd for C_16_H_16_N_3_O_6_S^+^, [M+H]^+^ 378.0755; Found 378.0753.

*Ethyl (E)-3-((Z)-2-((naphthalen-2-ylsulfonyl)imino)pyridin-1(2H)-yl)acrylate* (**4p**). 149.0 mg (78%), brown oil [*R*_f_ = 0.3(5) in 3:2 *v/v* ethyl acetate/60–90 petroleum ether]. IR *ν* 2924, 1715, 1638, 1493, 1371, 1269, 1124, 1070, 966, 856, 756, 733, 677, 658 cm^−1^; ^1^H NMR (400 MHz, CDCl_3_) *δ* 8.54 (s, 1H), 8.49 (d, *J* = 14.4 Hz, 1H), 7.91-8.00 (m, 3H), 7.86 (t, *J* = 8.2 Hz, 2H), 7.58 (s, 3H), 7.50 (t, *J* = 7.8 Hz, 1H), 6.54 (t, *J* = 7.0 Hz, 1H), 6.10 (d, *J* = 14.0 Hz, 1H), 4.22-4.28 (m, 2H), 1.28 (t, *J* = 7.0 Hz, 3H); ^13^C NMR (100 MHz, CDCl_3_) *δ* 164.7, 155.1, 141.0, 140.4, 140.1, 134.6, 133.8, 132.2, 129.3, 129.1, 128.3, 127.9, 127.2, 127.0, 122.7, 119.2, 114.6, 111.3, 61.4, 14.2; HRMS (ESI-TOF) (*m/z*). Calcd for C_20_H_19_N_2_O_4_S^+^, [M+H]^+^ 383.1060; Found 383.1058.

*Ethyl (E)-3-((Z)-2-((methylsulfonyl)imino)pyridin-1(2H)-yl)acrylate* (**4q**). 83.7 mg (62%), yellow solid, mp 161–162 °C [*R*_f_ = 0.2(5) in 3:2 *v/v* ethyl acetate/60–90 petroleum ether]. IR *ν* 1715, 1638, 1506, 1371, 1265, 1173, 1121, 1099, 1036, 968, 781, 758 cm^−1^; ^1^H NMR (400 MHz, CDCl_3_) *δ* 8.45 (d, *J* = 14.4 Hz, 1H), 7.70 (d, *J* = 9.6 Hz, 1H), 7.58 (d, *J* = 6.4 Hz, 1H), 7.48 (t, *J* = 7.8 Hz, 1H), 6.52 (t, *J* = 8.0 Hz,1H), 6.11 (d, *J* = 14.4 Hz, 1H), 4.25-4.31 (m, 2H), 3.10 (s, 3H), 1.33 (t, *J* = 6.4 Hz, 3H); ^13^C NMR (100 MHz, CDCl_3_) *δ* 164.9, 155.0, 140.5, 140.2, 133.4, 119.7, 114.0, 110.8, 61.5, 43.2, 14.3; HRMS (ESI-TOF) (*m/z*). Calcd for C_11_H_15_N_2_O_4_S^+^, [M+H]^+^ 271.0747; Found 271.0746.

*Ethyl (E)-3-((Z)-2-((ethylsulfonyl)imino)pyridin-1(2H)-yl)acrylate* (**4r**). 95.2 mg (67%), yellow solid, mp 107–108 °C (*R*_f_ = 0.3 in 3:2 *v/v* ethyl acetate/60–90 petroleum ether). IR *ν* 2924, 1717, 1639, 1520, 1508, 1265, 1173, 1121, 1099, 1038, 961, 756, 733 cm^−1^; ^1^H NMR (400 MHz, CDCl_3_) *δ* 8.48 (d, *J* = 14.4 Hz, 1H), 7.73 (d, *J* = 8.8 Hz,1H), 7.56 (d, *J* = 6.8 Hz, 1H), 7.46 (t, *J* = 8.0 Hz, 1H), 6.50 (t, *J* = 6.0 Hz, 1H), 6.10 (d, *J* = 14.8 Hz, 1H), 4.25–4.30 (m, 2H), 3.13–3.18 (m, 2H), 1.45 (t, *J* = 6.0 Hz, 3H), 1.33 (t, *J* = 5.8 Hz, 3H); ^13^C NMR (100 MHz, CDCl_3_) *δ* 164.9, 155.2, 140.3, 140.2, 133.2, 120.0, 113.6, 110.7, 61.4, 49.6, 14.3, 8.7; HRMS (ESI-TOF) (*m/z*). Calcd for C_12_H_17_N_2_O_4_S^+^, [M+H]^+^ 285.0904; Found 285.0902.

*Ethyl (E)-3-((Z)-2-((propylsulfonyl)imino)pyridin-1(2H)-yl)acrylate* (**4s**). 104.3 mg (70%), yellow solid, mp 125–126 °C (*R*_f_ = 0.3 in 1:1 *v/v* ethyl acetate/60–90 petroleum ether). IR *ν* 1715, 1639, 1518, 1508, 1383, 1371, 1265, 1167, 1119, 1098, 1036, 959, 754 cm^−1^; ^1^H NMR (400 MHz, CDCl_3_) *δ* 8.47 (d, *J* = 14.4 Hz, 1H), 7.73 (d, *J* = 9.2 Hz, 1H), 7.56 (d, *J* = 7.2 Hz, 1H), 7.45 (t, *J* = 7.8 Hz, 1H), 6.49 (t, *J* = 7.2 Hz, 1H), 6.09 (d, *J* = 14.4 Hz, 1H), 4.24-4.30 (m, 2H), 3.12 (t, *J* = 7.8 Hz, 2H), 1.93-1.98 (m, 2H), 1.33 (t, *J* = 6.2 Hz, 3H), 1.08 (t, *J* = 6.4 Hz, 3H); ^13^C NMR (100 MHz, CDCl_3_) *δ* 164.9, 155.1, 140.23, 140.15, 133.1, 119.9, 113.5, 110.6, 61.4, 57.0, 17.7, 14.3, 13.2; HRMS (ESI-TOF) (*m/z*). Calcd for C_13_H_19_N_2_O_4_S^+^, [M+H]^+^ 299.1060; Found 299.1059.

*Ethyl (E)-3-((Z)-2-((isobutylsulfonyl)imino)pyridin-1(2H)-yl)acrylate* (**4t**). 90.5 mg (58%), brown solid, mp 77–78 °C (*R*_f_ = 0.4 in 1:1 *v/v* ethyl acetate/60–90 petroleum ether). IR *ν* 1717, 1641, 1520, 1510, 1385, 1373, 1269, 1167, 1121, 1099, 1038, 962, 733 cm^−1^; ^1^H NMR (400 MHz, CDCl_3_) *δ* 8.48 (d, *J* = 14.4 Hz, 1H), 7.73 (d, *J* = 9.2 Hz, 1H), 7.56 (d, *J* = 6.8 Hz, 1H), 7.45 (t, *J* = 8.0 Hz, 1H), 6.49 (t, *J* = 6.4 Hz, 1H), 6.09 (d, *J* = 14.4 Hz, 1H), 4.25–4.31 (m, 2H), 3.07 (d, *J* = 7.2 Hz, 2H), 2.35–2.44 (m, 1H), 1.33 (t, *J* = 14.4 Hz, 3H), 1.14 (d, *J* = 7.2 Hz, 6H); ^13^C NMR (100 MHz, CDCl_3_) *δ* 164.9, 155.0, 140.22, 140.19, 133.1, 120.0, 113.5, 110.6, 62.9, 61.4, 25.2, 22.9 (2C), 14.3; HRMS (ESI-TOF) (*m/z*). Calcd for C_14_H_21_N_2_O_4_S^+^, [M+H]^+^ 313.1217; Found 313.1216.

*Ethyl (E)-3-((Z)-2-((benzylsulfonyl)imino)pyridin-1(2H)-yl)acrylate* (**4u**). 135.0 mg (78%), yellow solid, mp 110–112 °C (*R*_f_ = 0.3 in 1:1 *v/v* ethyl acetate/60–90 petroleum ether). IR *ν* 2920, 1717, 1639, 1520, 1510, 1275, 1099, 1036, 964, 798, 762, 700, 683 cm^−1^; ^1^H NMR (400 MHz, CDCl_3_) *δ* 8.38 (d, *J* = 14.4 Hz, 1H), 7.53 (d, *J* = 8.8 Hz, 2H), 7.37-7.43 (m, 3H), 7.26 (s, 2H), 6.47 (t, *J* = 7.2 Hz, 1H), 6.08 (d, *J* = 14.4 Hz, 1H), 4.31-4.36 (m, 4H), 1.37 (t, *J* = 8.0 Hz, 3H); ^13^C NMR (100 MHz, CDCl_3_) *δ* 164.7, 155.2, 140.6, 140.3, 134.2, 133.5, 132.6, 131.0, 129.8, 129.5, 128.5, 119.5, 114.5, 111.0, 61.5, 60.4, 14.4; HRMS (ESI-TOF) (*m/z*). Calcd for C_17_H_19_N_2_O_4_S^+^, [M+H]^+^ 347.1060; Found 347.1059.

*Ethyl (E)-3-((Z)-2-(((3-chloropropyl)sulfonyl)imino)pyridin-1(2H)-yl)acrylate* (**4v**). 101.3 mg (61%), yellow solid, mp 108–110 °C (*R*_f_ = 0.3 in 3:2 *v/v* ethyl acetate/60–90 petroleum ether). IR *ν* 1715, 1638, 1508, 1267, 1169, 1119, 1096, 1038, 961, 907, 756, 735 cm^−1^; ^1^H NMR (400 MHz, CDCl_3_) *δ* 8.43 (d, *J* = 14.4 Hz, 1H), 7.72 (d, *J* = 11.6 Hz, 1H), 7.58 (d, *J* = 7.2 Hz, 1H), 7.50 (t, *J* = 7.0 Hz, 1H), 6.54 (t, *J* = 8.2 Hz, 1H), 6.10 (d, *J* = 14.4 Hz, 1H), 4.26–4.31 (m, 2H), 3.72 (d, *J* = 4.0 Hz, 2H), 3.31 (t, *J* = 6.4 Hz, 2H), 2.39 (t, *J* = 6.8 Hz, 2H), 1.33 (t, *J* = 6.4 Hz, 3H); ^13^C NMR (100 MHz, CDCl_3_) *δ* 164.8, 155.2, 140.6, 140.1, 133.4, 119.8, 114.3, 111.0, 61.5, 52.2, 43.3, 27.5, 14.3; HRMS (ESI-TOF) (*m/z*). Calcd for C_13_H_18_ClN_2_O_4_S^+^, [M+H]^+^ 333.0671; Found 333.0669.

*Ethyl (E)-3-((Z)-2-((((7,7-dimethyl-2-oxobicyclo [2.2.1] heptan-1-yl) methyl) sulfonyl) imino) pyridin-1(2H)-yl)acrylate* (**4w**). 172.6 mg (85%), yellow oil (*R*_f_ = 0.3 in 1:1 *v/v* ethyl acetate/60–90 petroleum ether). IR *ν* 2961, 2920, 1744, 1717, 1640, 1520, 1508, 1373, 1267, 1173, 1121, 1101, 1038, 962, 760 cm^−1^; ^1^H NMR (400 MHz, CDCl_3_) *δ* 8.43 (d, *J* = 15.2 Hz, 1H), 7.83 (d, *J* = 12.0 Hz, 1H), 7.58 (d, *J* = 7.2 Hz, 1H), 7.49 (t, *J* = 8.0 Hz, 1H), 6.53 (t, *J* = 7.4 Hz, 1H), 6.07 (d, *J* = 14.4 Hz, 1H), 4.25–4.30 (m, 2H), 3.67 (d, *J* = 14.8 Hz, 1H), 3.06 (d, *J* = 14.8 Hz, 1H), 2.67 (t, *J* = 12.6 Hz, 1H), 2.34 (d, *J* = 18.4 Hz, 1H), 2.05 (d, *J* = 14.0 Hz, 2H), 1.91 (d, *J* = 18.8 Hz, 1H), 1.74–1.81 (m, 1H), 1.42 (t, *J* = 10.8 Hz, 1H), 1.32 (t, *J* = 6.8 Hz, 3H), 1.12 (s. 3H), 0.87 (s, 3H); ^13^C NMR (100 MHz, CDCl_3_) *δ* 215.5, 164.8, 155.3, 140.7, 140.5, 133.4, 119.6, 114.1, 110.9, 61.4, 58.5, 50.8, 48.2, 42.8, 42.7, 27.2, 24.6, 20.1, 19.9, 14.3; HRMS (ESI-TOF) (*m/z*). Calcd for C_20_H_27_N_2_O_5_S^+^, [M+H]^+^ 407.1635; Found 407.1634.

### 3.3. UV–Vis and Fluorescence Measurements

The reported UV–vis spectra (Figure 5 and Figure 6) were recorded on a Persee TU-1901 double-beam spectrophotometer (VWR International) in a quartz cuvette of 1.00 cm optical length. A 3.00 cm^3^ solution was prepared from each sample and the reference solution was the equivalent volume of the pure solvent (MeOH). Steady-state fluorescence measurements were carried out using an F-280 fluorescence spectrophotometer (Tianjin Gangdong Sci. and Tech. Development. Co., Ltd., Tianjin, China) equipped with a Xe lamp light source. The excitation and emission spectra were recorded at 20 °C using 2.5 nm excitation, 2.5 nm emission bandwidth and a 200 nm/min scan rate. For UV–vis and fluorescence measurements, the compounds under study were dissolved in MeOH at a concentration of 1.0 mM and then diluted to (3.0–4.0) × 10^−6^ M. Fluorescence quantum yields were determined using spectroscopic-grade MeOH at room temperature and using quinine sulfate solution (*Φ*_F_ = 0.55 in 0.5 mol/L H_2_SO_4_) as the reference; values were calculated using Equation (1) given in reference [51,52].

## 4. Conclusions

The present study establishes that pyridin-1(2*H*)-ylacrylates, which are readily prepared compounds, have significant potential as a new class of small-molecule fluorophores. Furthermore, variations in the substitution patterns about the parent framework seem to allow for the rational tuning of their photophysical properties, suggesting they could be usefully applied in the study of, *inter alia,* luminescent materials.

## 5. Patents

There have been no patent filings associated with this work.

## Figures and Tables

**Table 1:** Optimization of reaction conditions ^a^.

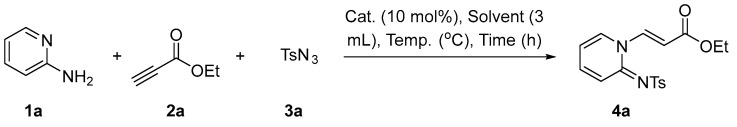					
Entry	Cat. (10 mol%)	Solvent (10 mol%)	Temp. (°C)	Time (h)	Yield (%) ^b^
1	Fe(C_5_H_5_)_2_	DCE	60	12	68
2	FeCl_3_	DCE	60	12	18
3	Fe(OAc)_2_	DCE	60	12	42
4	Fe(acac)_3_	DCE	60	12	54
5	[CpRhCl_2_]_2_	DCE	60	12	10
6	Pd(OAc)_2_	DCE	60	12	23
7	CuI	DCE	60	12	0
8	–	DCE	60	12	70
9	–	Toluene	60	12	62
10	–	CH_2_Cl_2_	60	12	45
11	–	THF	60	12	66
12	–	MeCN	60	12	78
13	–	1,4-Dioxane	60	12	60
14	–	DMF	60	12	32
15	−	DMSO	60	12	30
16	–	MeCN	80	12	78
17	–	MeCN	100	12	70
18	–	MeCN	40	12	64
19	–	MeCN	rt	12	26
20	–	MeCN	60	16	78
21	–	MeCN	60	24	72
22	–	MeCN	60	10	76
23	–	MeCN	60	8	70

^a^ Reaction conditions: A solution of **1a** (0.5 mmol) and cat. 10 mol% in the relevant solvent (3 mL) was treated with **2a** (2.0.) and **3a** (1.2 eq.) and then stirred under the specified conditions. ^b^ Yield of isolated product.

**Table 2:** Key photophysical properties of the pyridin-1(2*H*)-ylacrylates **4a**–**w** ^a^.

Compound	λ_max_^Abs^ (nm)	ε ^b^ (mM^−1^ cm^−1^)	λ_max_^Em^ (nm)	Stokes Shift (nm)	*Φ*_F_ ^c^	Brightness ^d^ (mM^−1^ cm^−1^)
**4a**	238, 365	91	469	104	0.13	11.83
**4b**	240, 356	95	468	112	0.17	16.15
**4c**	232, 348	103	485	137	0.55	56.65
**4d**	234, 343	114	473	130	0.31	35.34
**4e**	241, 359	94	469	110	0.34	31.96
**4f**	230, 344	97	490	146	0.61	59.17
**4g**	239, 364	108	473	109	0.17	18.36
**4h**	237, 362	95	469	107	0.15	14.25
**4i**	239, 362	97	467	105	0.21	20.37
**4j**	237, 362	149	465	103	0.27	40.23
**4k**	237, 359	184	466	107	0.14	25.76
**4l**	236, 360	151	466	106	0.08	12.08
**4m**	239, 365	114	473	108	0.72	82.08
**4n**	235, 360	119	467	107	0.04	4.76
**4o**	235, 360	209	461	101	0.12	25.08
**4p**	231, 363	235	468	105	0.31	72.85
**4q**	236, 364	91	468	104	0.11	10.01
**4r**	237, 365	95	468	103	0.17	16.15
**4s**	238, 366	95	468	102	0.21	19.95
**4t**	235, 366	119	469	103	0.18	21.42
**4u**	237, 362	126	466	104	0.27	34.02
**4v**	237, 362	131	466	104	0.43	56.33
**4w**	237, 363	151	471	108	0.25	37.75

^a^ Reported data obtained as solutions in MeOH; ^b^ The molar extinction coefficient (ε) of the more red-shifted absorption band; ^c^ Cited values determined using spectroscopic-grade MeOH at room temperature and using a quinine sulfate solution as reference (*Φ*_F_ = 0.55 in 0.5 mol/L H_2_SO_4_). ^d^ Calculated as the product of ε and *Φ*_F_.

## Data Availability

All data generated or analyzed during this study are included in this published article and the associated Supplementary Information files or are available from the corresponding author W.Y. on reasonable request.

## Supplementary Materials

The following supporting information can be downloaded at https://www.mdpi.com/article/10.3390/molecules28186511/s1. ^1^H and ^13^C NMR spectra.

## Sample Availability

Samples of the compounds **4a**–**4w** are available (contact W.Y.).
