# Supplementary material for: Synthesis of Pyridin-1(2H)-ylacrylates and the Effects of Different Functional Groups on Their Fluorescence
Source: Molecules. 2023 Sep 8;28(18):6511. doi: 10.3390/molecules28186511 (PMC10536652; doi:10.3390/molecules28186511)

Supporting Information for

Synthesis of Pyridin-1(2*H*)-ylacrylates and the Effects of Different Functional Groups on their Fluorescence

Weiguang Yang [1,*], Danyang Luo [1], Guanrong Li [1], QiaoLi Luo [2], Martin G. Banwell [1,3,*] and Lanmei Chen [1,*]

[1] Key Laboratory of Big Data Mining and Precision Drug Design of Guangdong Medical University, The Marine Biomedical Research Institute, Guangdong Medical University, Zhanjiang, 524023, China;

[2] School of Chemistry and Chemical Engineering, Lingnan Normal University, Zhanjiang 524048, P. R. China;

[3] Institute for Advanced and Applied Chemical Synthesis (IAACS), Jinan University, Guangzhou 510632/Zhuhai 519070, China

*****Correspondence: 09ywg@163.com (W.Y.); martin.banwell@anu.edu.au (M.B.); lanmeichen@126.com (L.C.).

Table of Contents

1. ^{1}H and $^{13}C\{^{1}H\}$NMR Spectra……………………………………………….S2

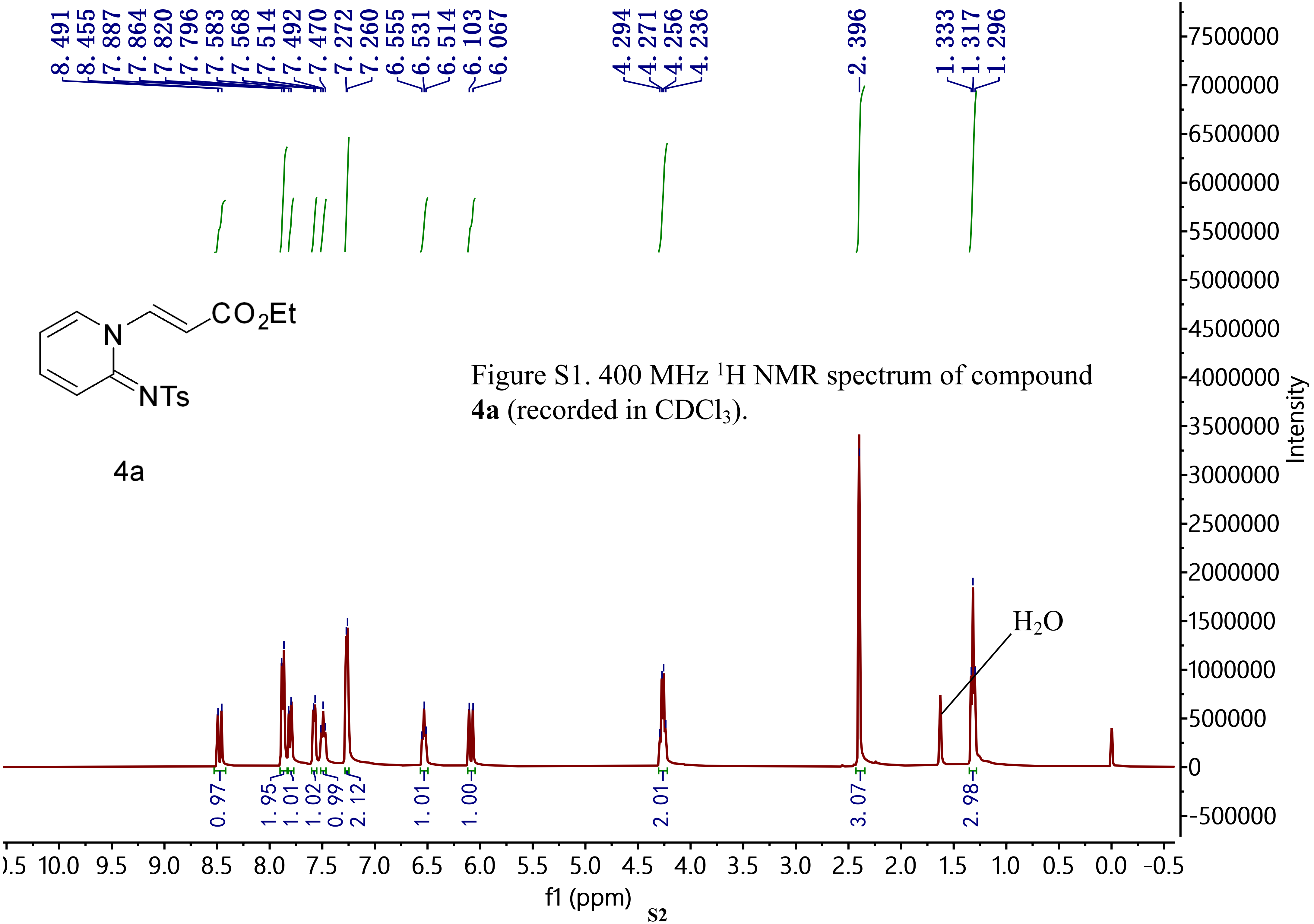

Figure S1. 400 MHz ^{1}H NMR spectrum of compound **4a** (recorded in $CDCl_3$).

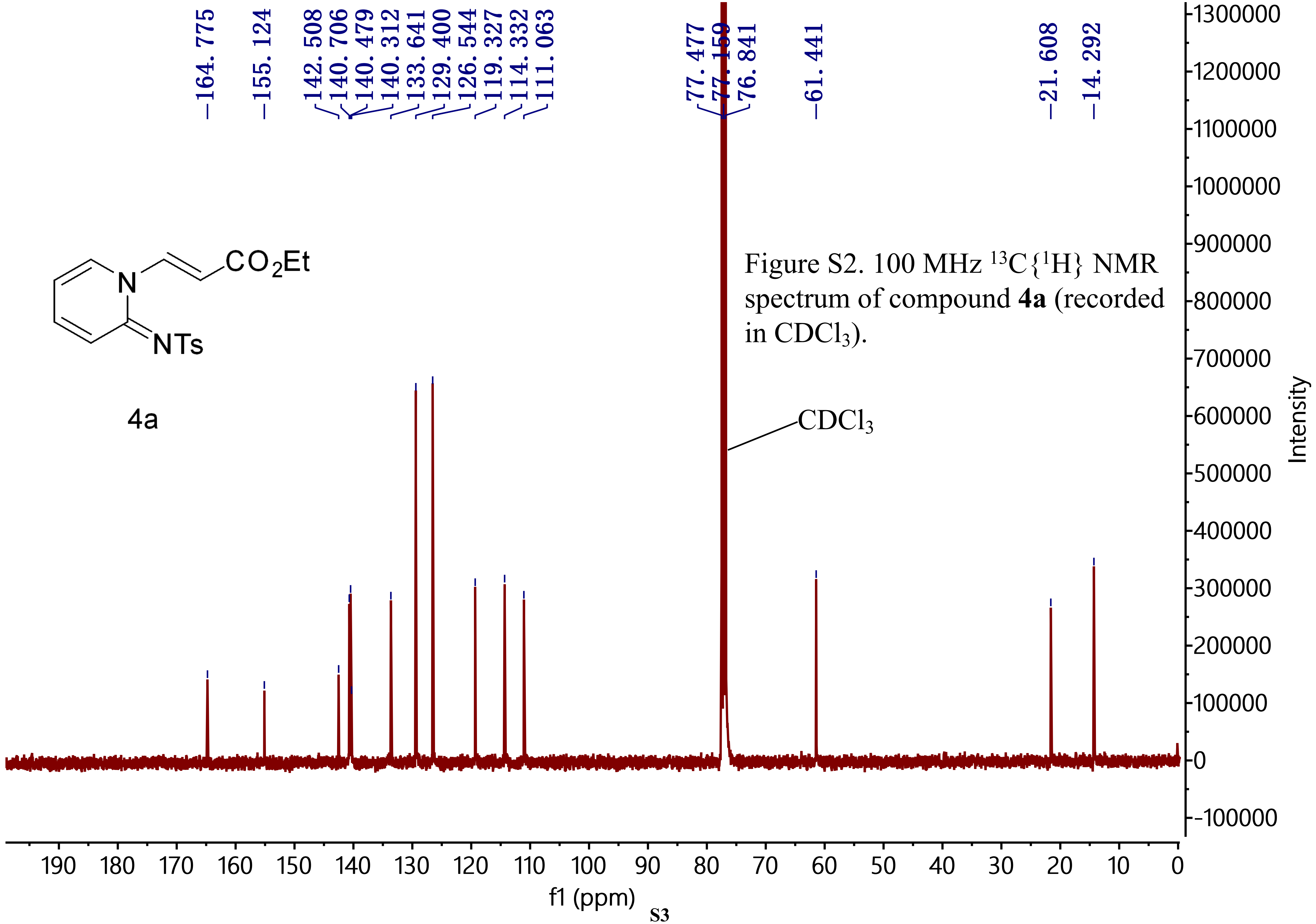

Figure S2. 100 MHz $^{13}C\{^{1}H\}$ NMR spectrum of compound **4a** (recorded in $CDCl_3$).

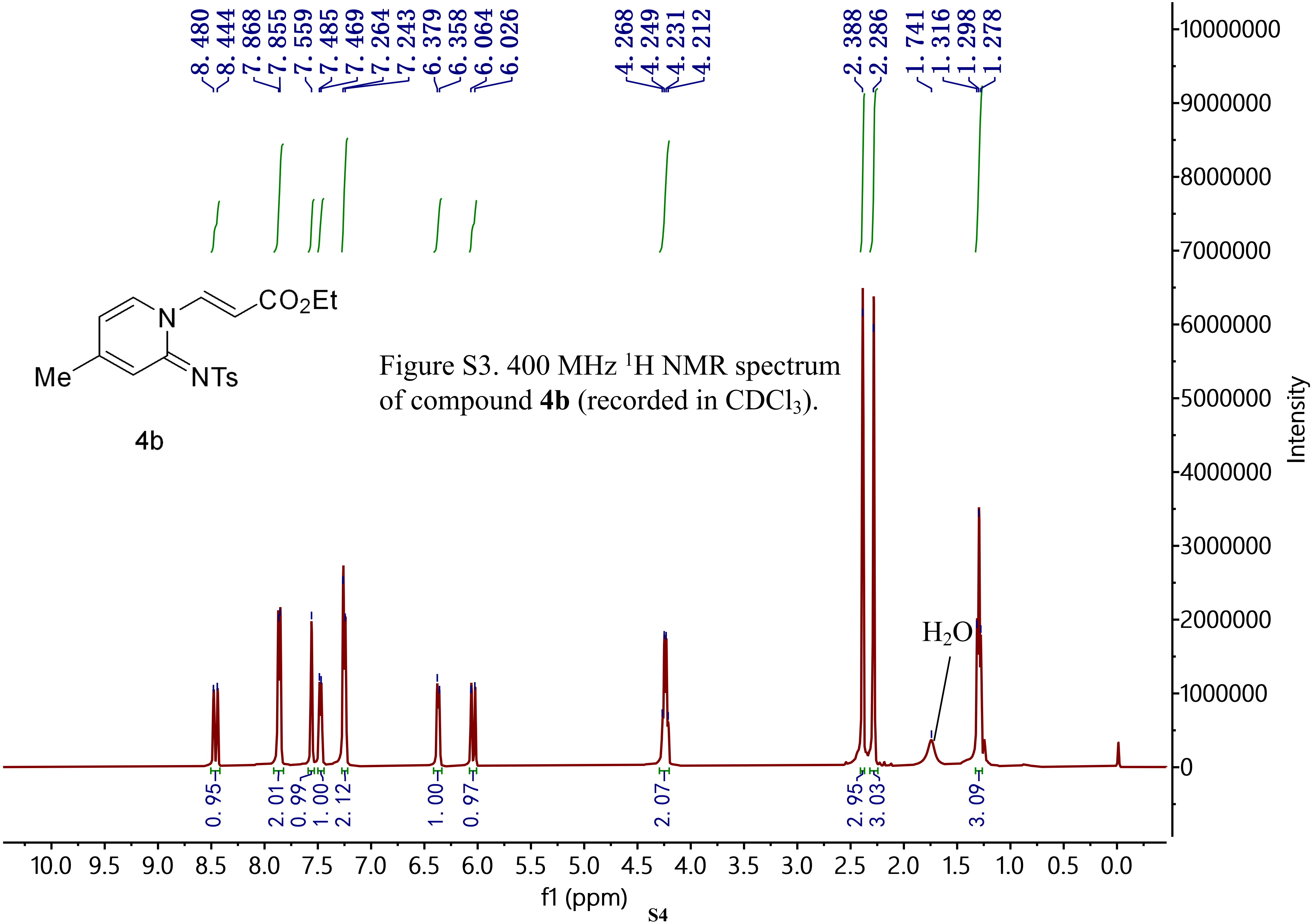

Figure S3. 400 MHz ^{1}H NMR spectrum of compound **4b** (recorded in $CDCl_3$).

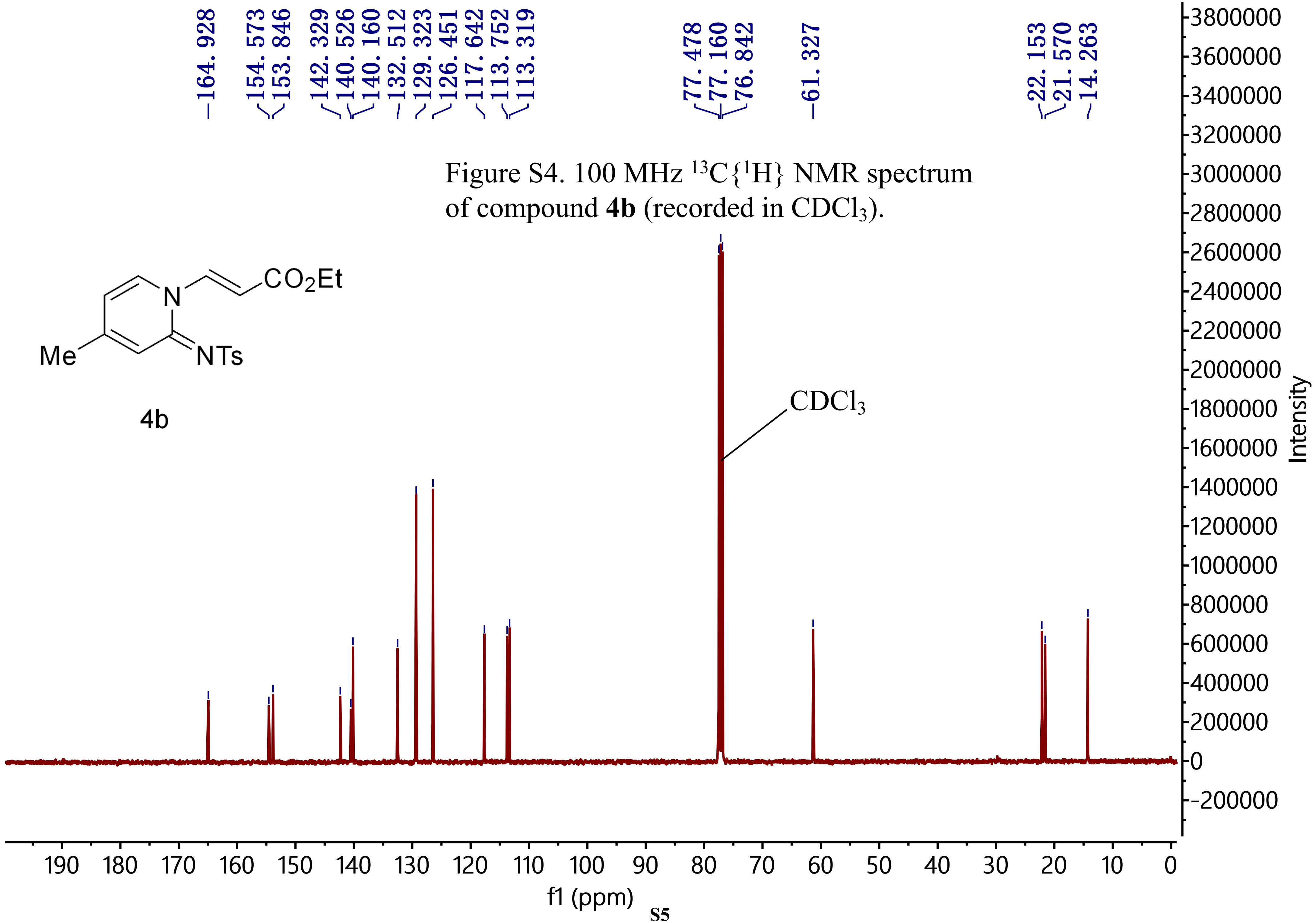

Figure S4. 100 MHz $^{13}C\{^{1}H\}$ NMR spectrum of compound **4b** (recorded in $CDCl_3$).

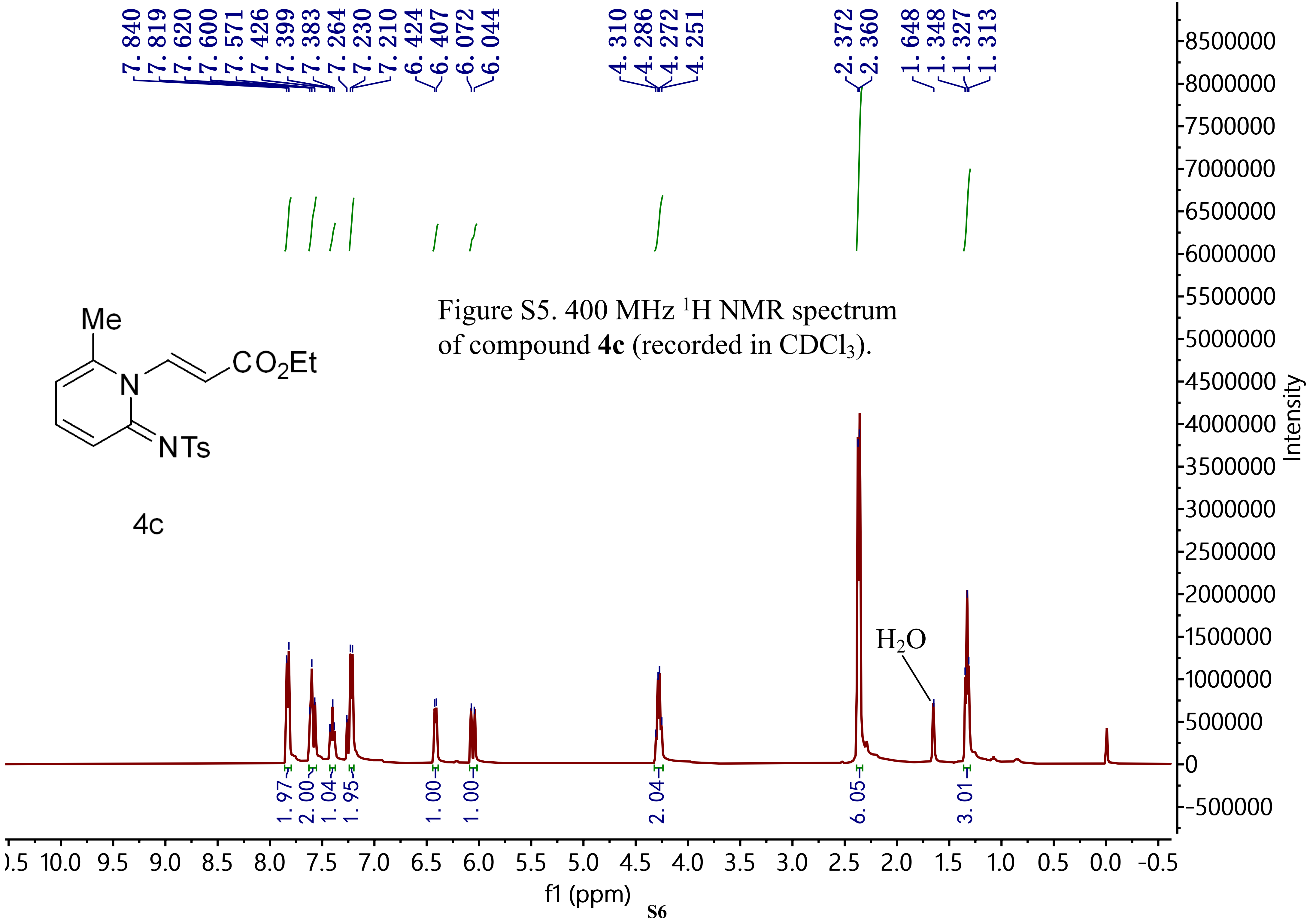

4c

Figure S5. 400 MHz ^{1}H NMR spectrum of compound **4c** (recorded in $CDCl_3$).

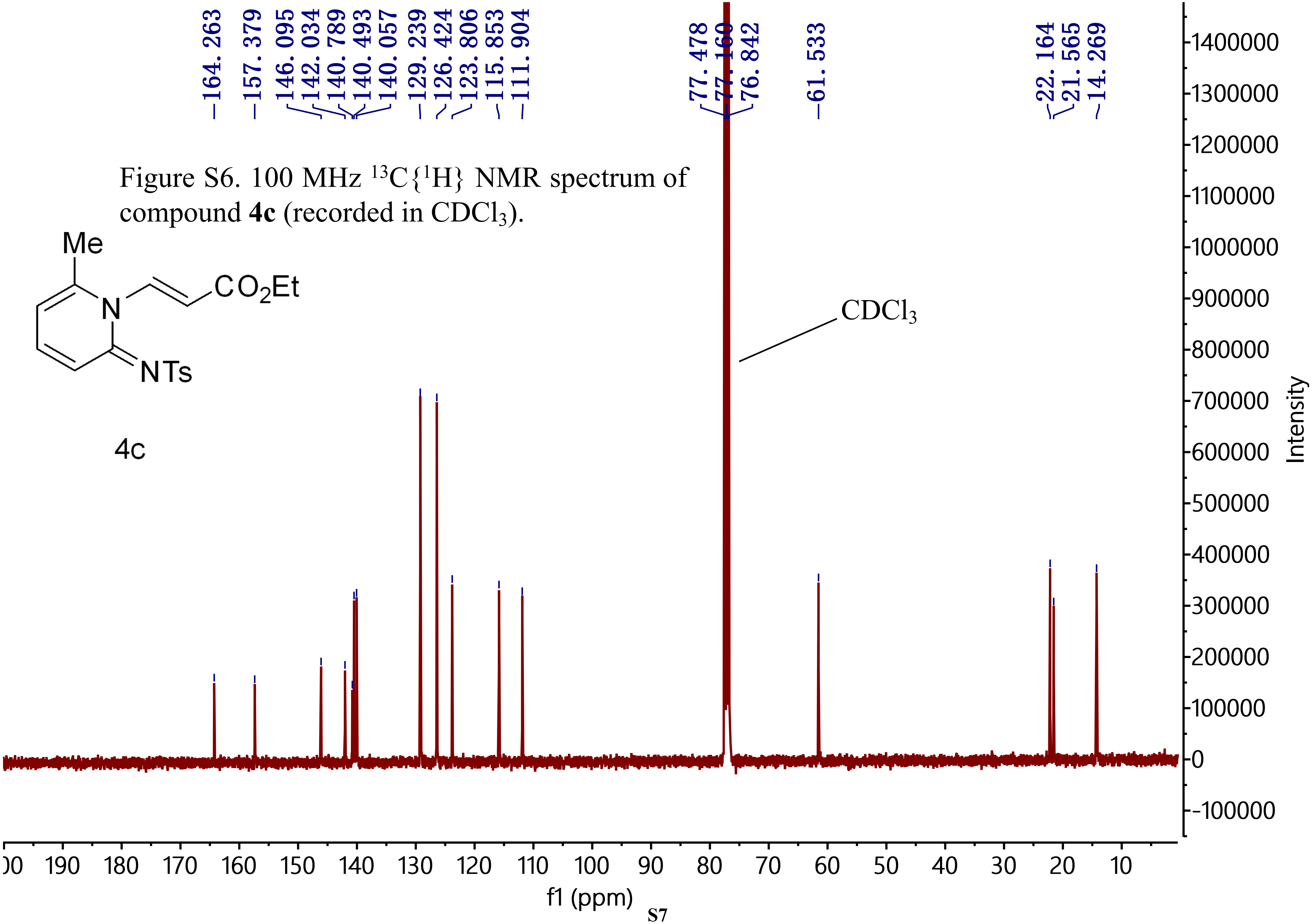

Figure S6. 100 MHz $^{13}C\{^{1}H\}$ NMR spectrum of compound **4c** (recorded in $CDCl_3$).

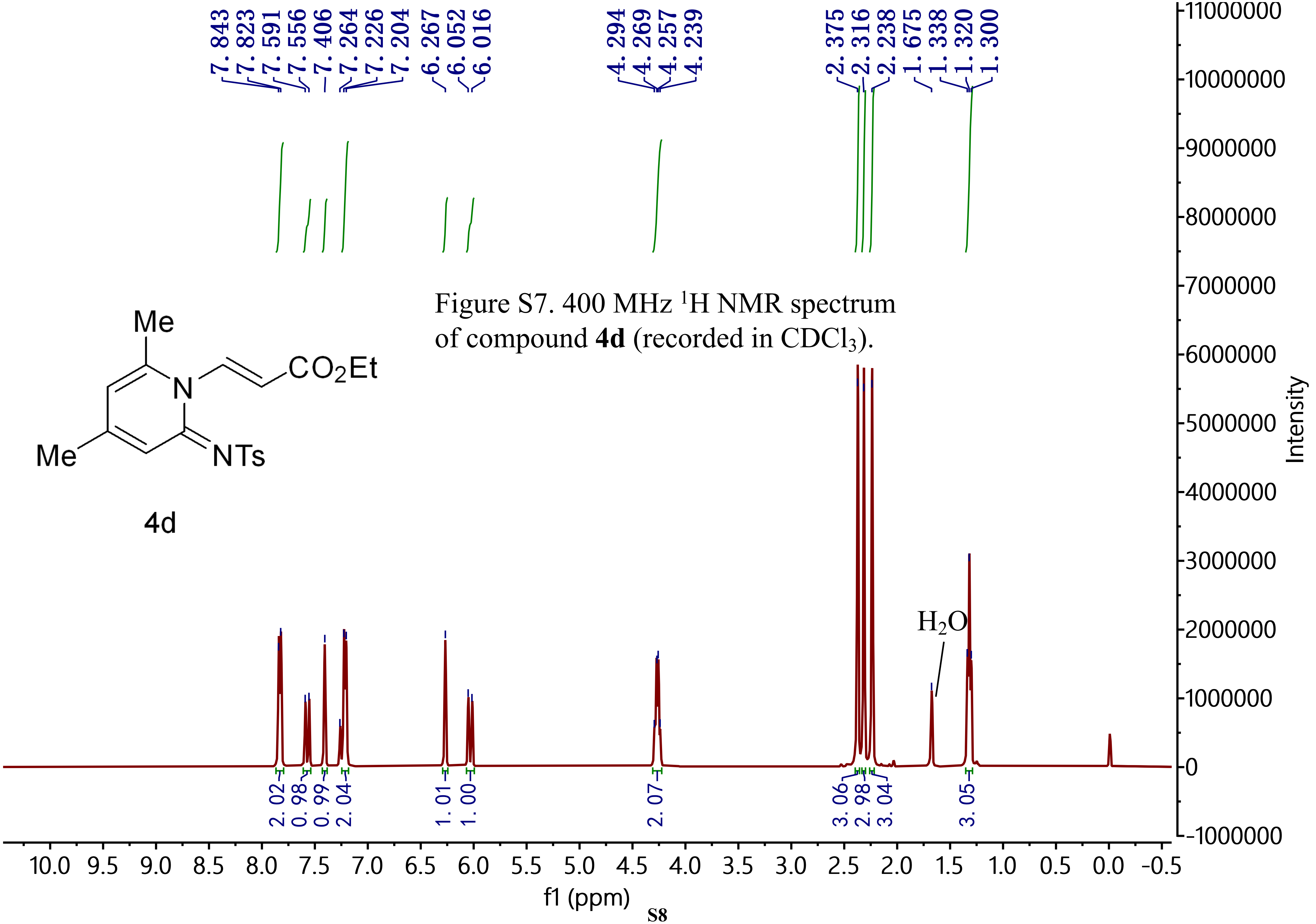

Figure S7. 400 MHz ^{1}H NMR spectrum of compound **4d** (recorded in $CDCl_3$).

4d

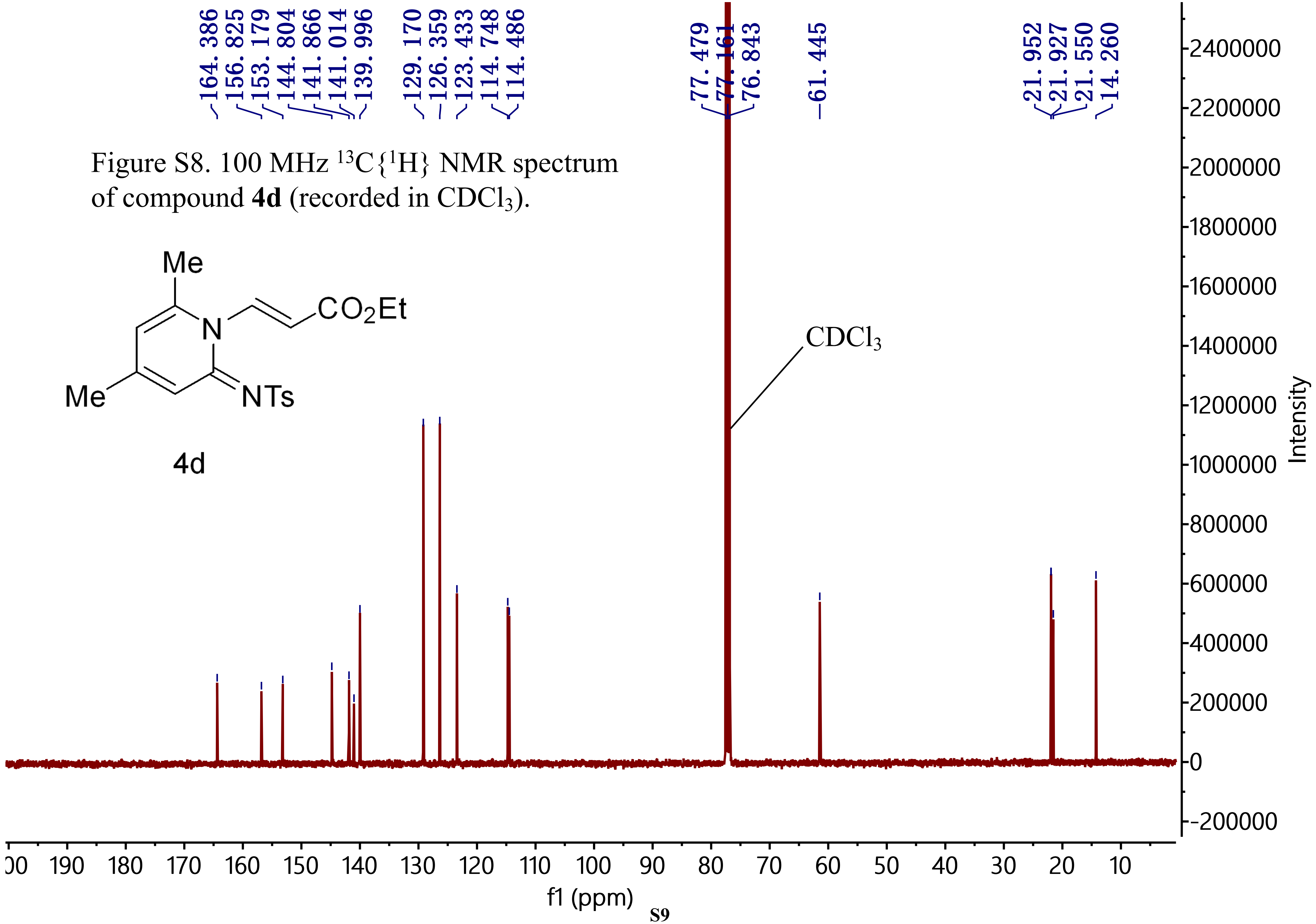

Figure S8. 100 MHz $^{13}C\{^1H\}$ NMR spectrum of compound **4d** (recorded in $CDCl_3$).

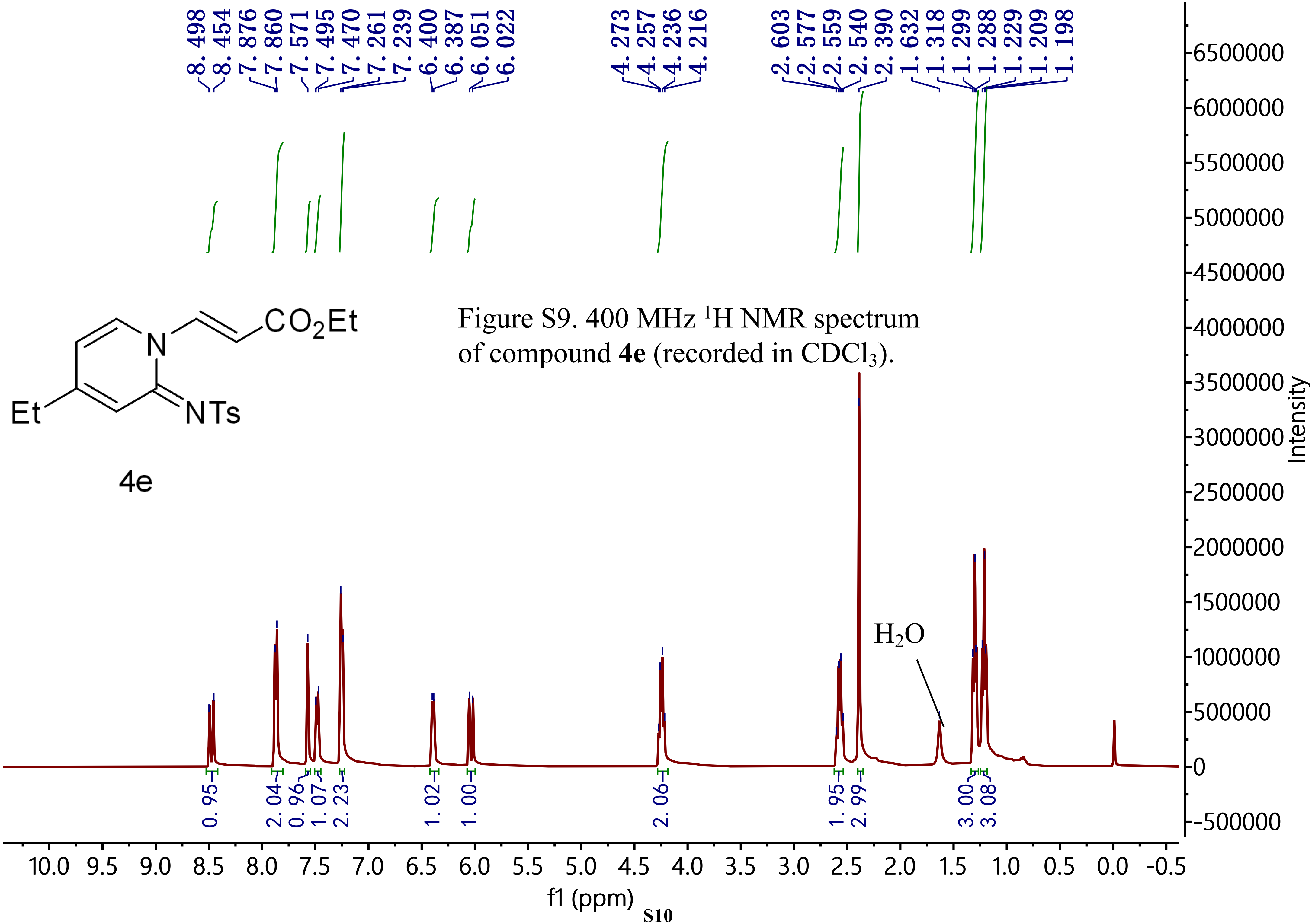

Figure S9. 400 MHz ^{1}H NMR spectrum of compound **4e** (recorded in $CDCl_3$).

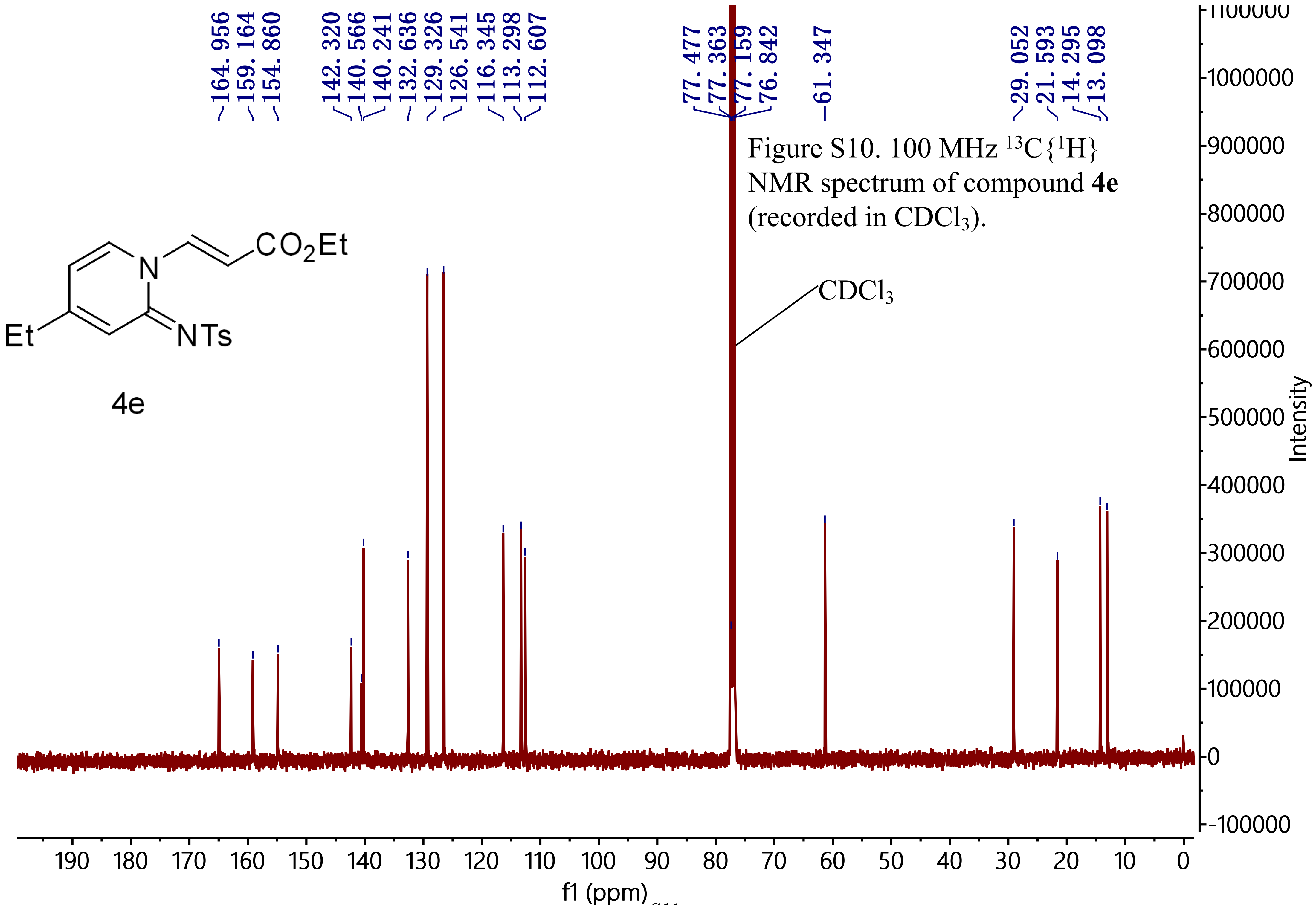

Figure S10. 100 MHz $^{13}C\{^1H\}$ NMR spectrum of compound **4e** (recorded in $CDCl_3$).

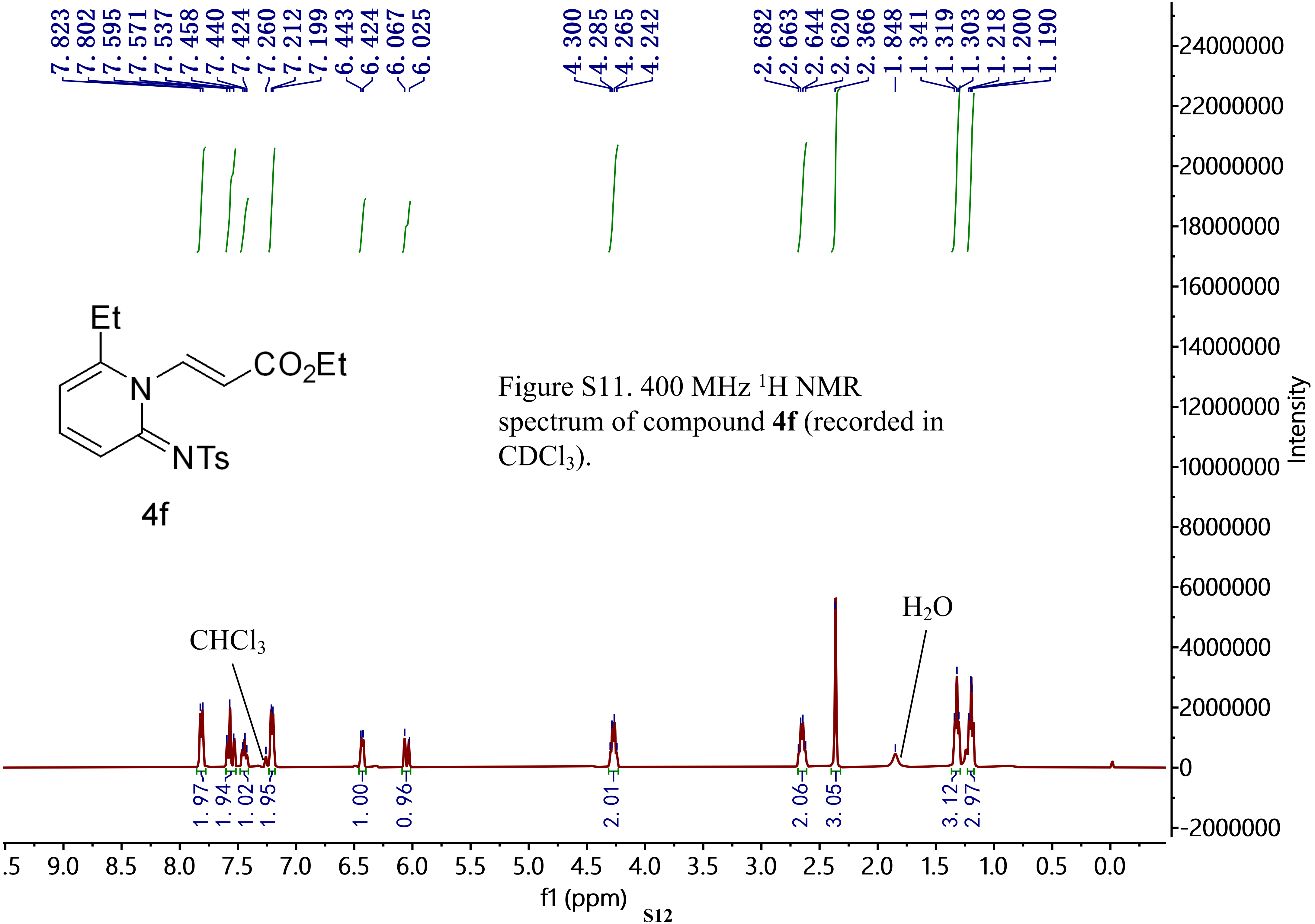

Figure S11. 400 MHz ^{1}H NMR spectrum of compound **4f** (recorded in $CDCl_3$).

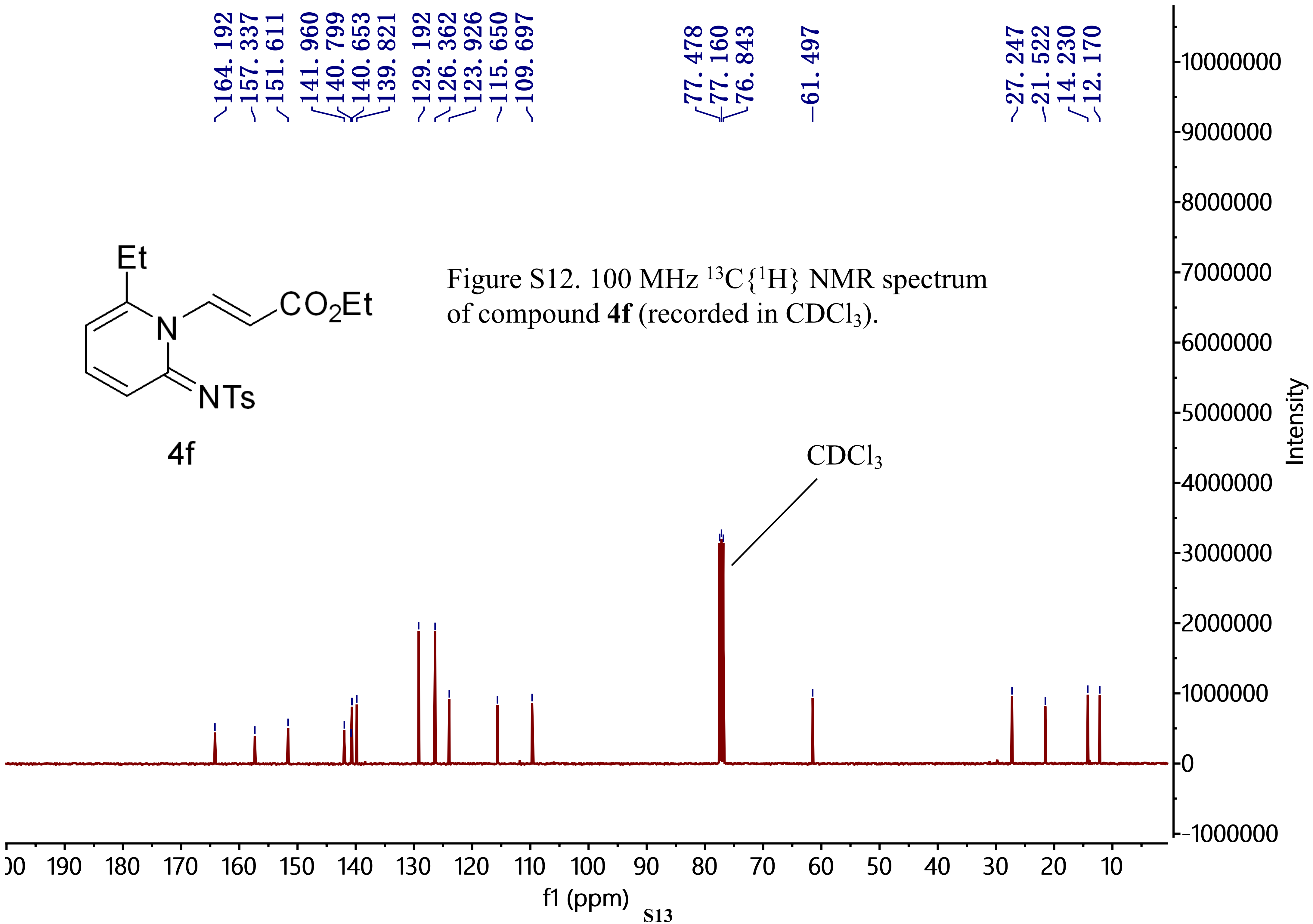

Figure S12. 100 MHz $^{13}C\{^{1}H\}$ NMR spectrum of compound **4f** (recorded in $CDCl_3$).

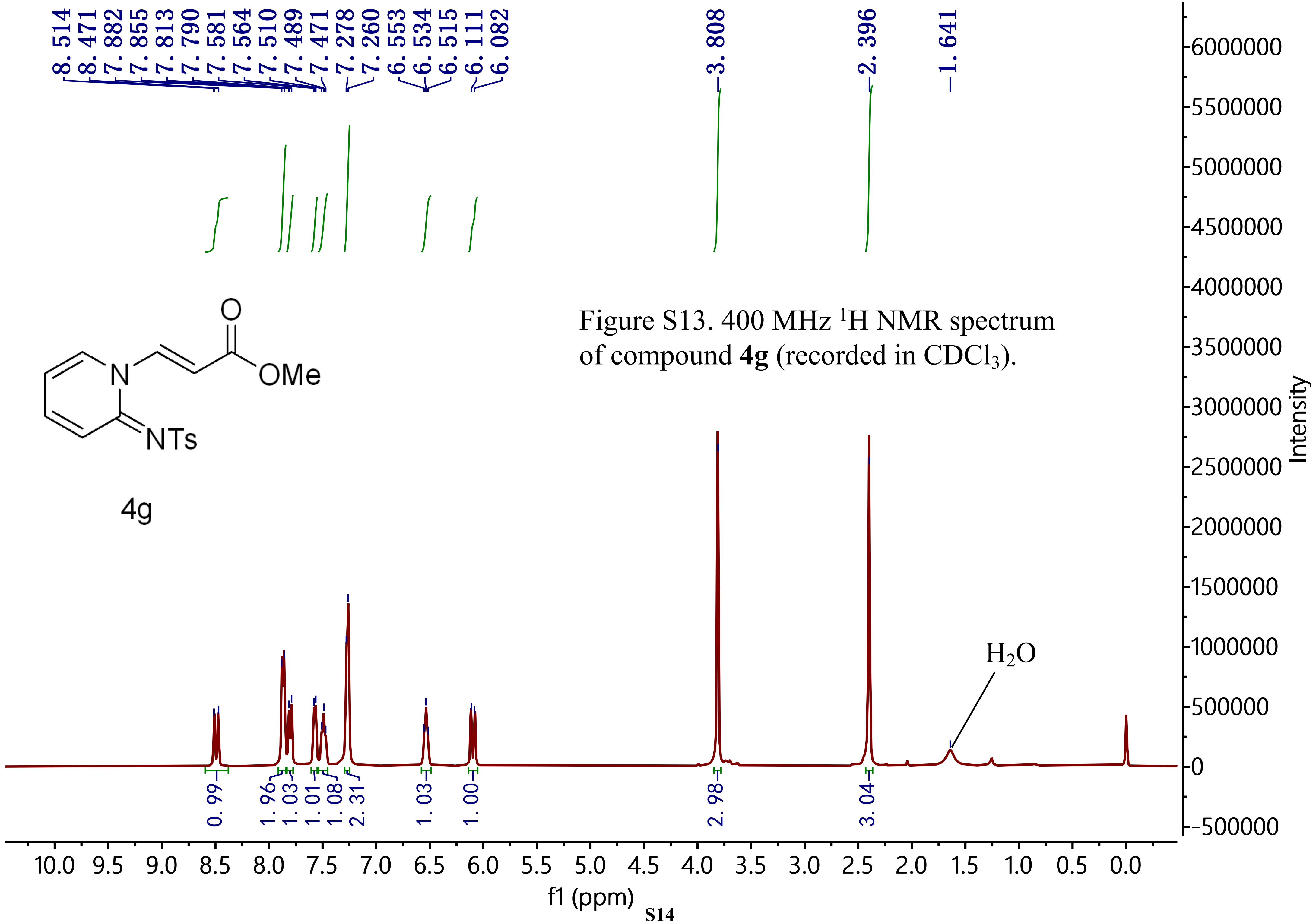

Figure S13. 400 MHz ^{1}H NMR spectrum of compound **4g** (recorded in $CDCl_3$).

Figure S14. 100 MHz $^{13}C\{^{1}H\}$ NMR spectrum of compound **4g** (recorded in $CDCl_3$).

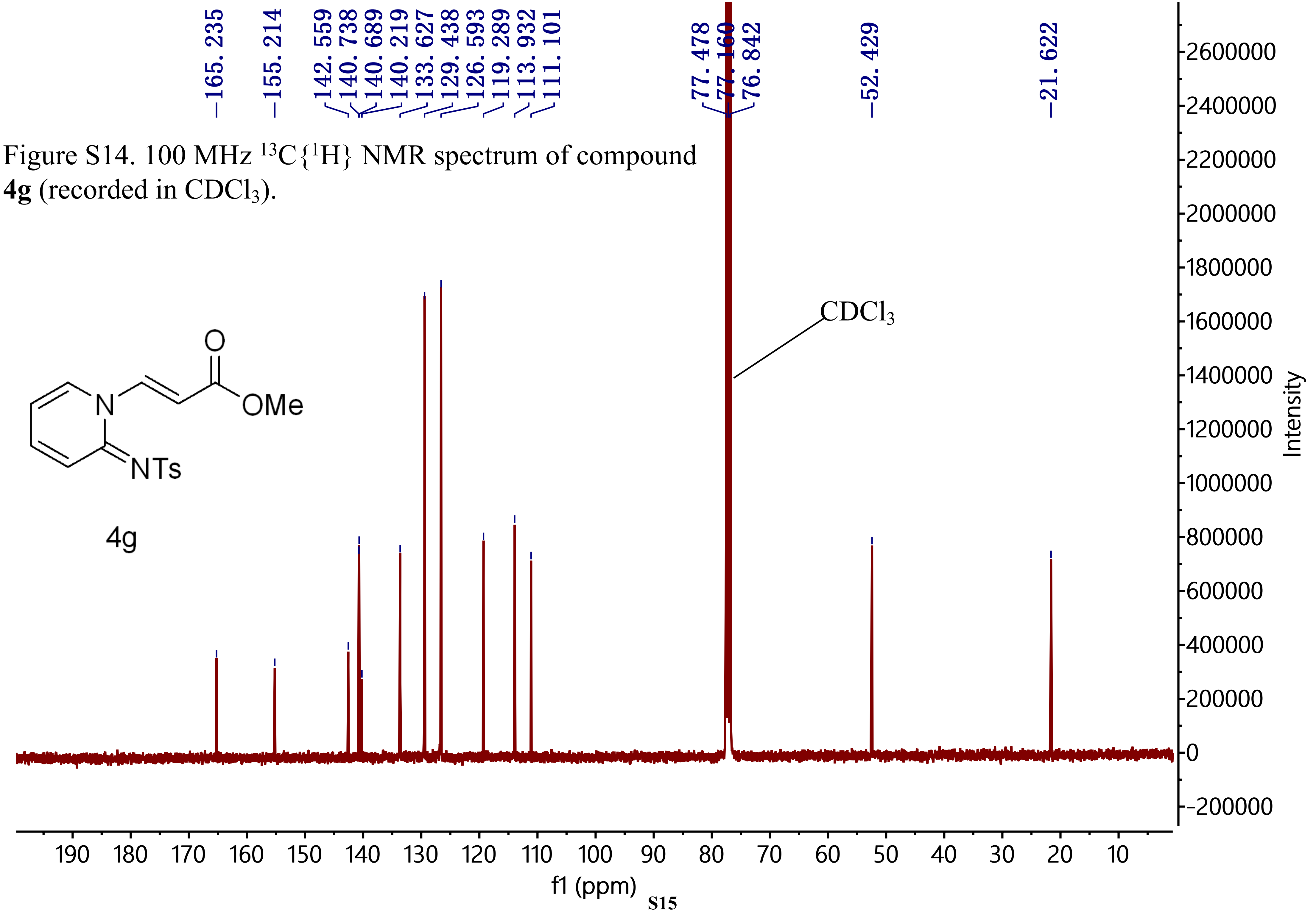

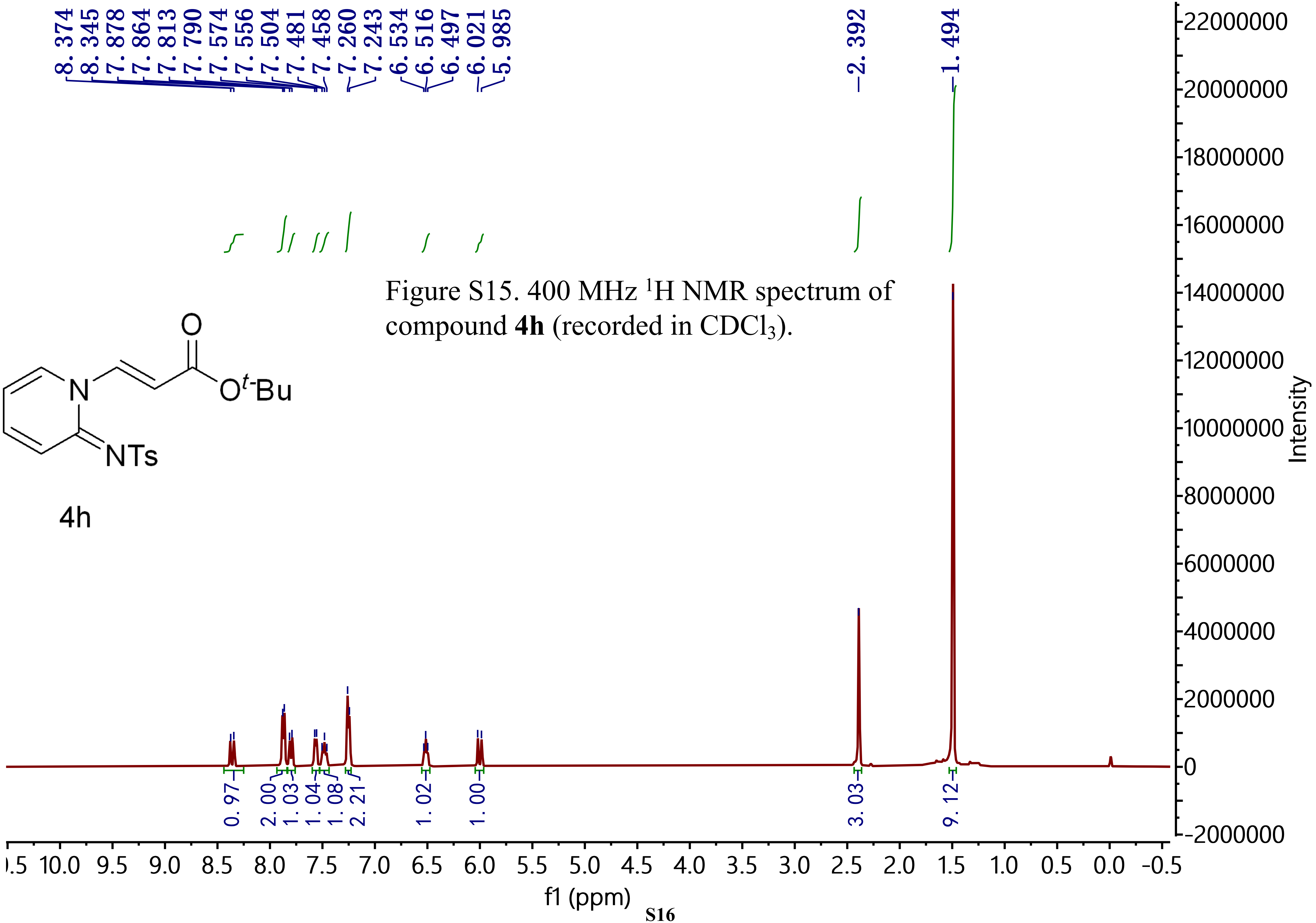

Figure S15. 400 MHz ^{1}H NMR spectrum of compound **4h** (recorded in $CDCl_3$).

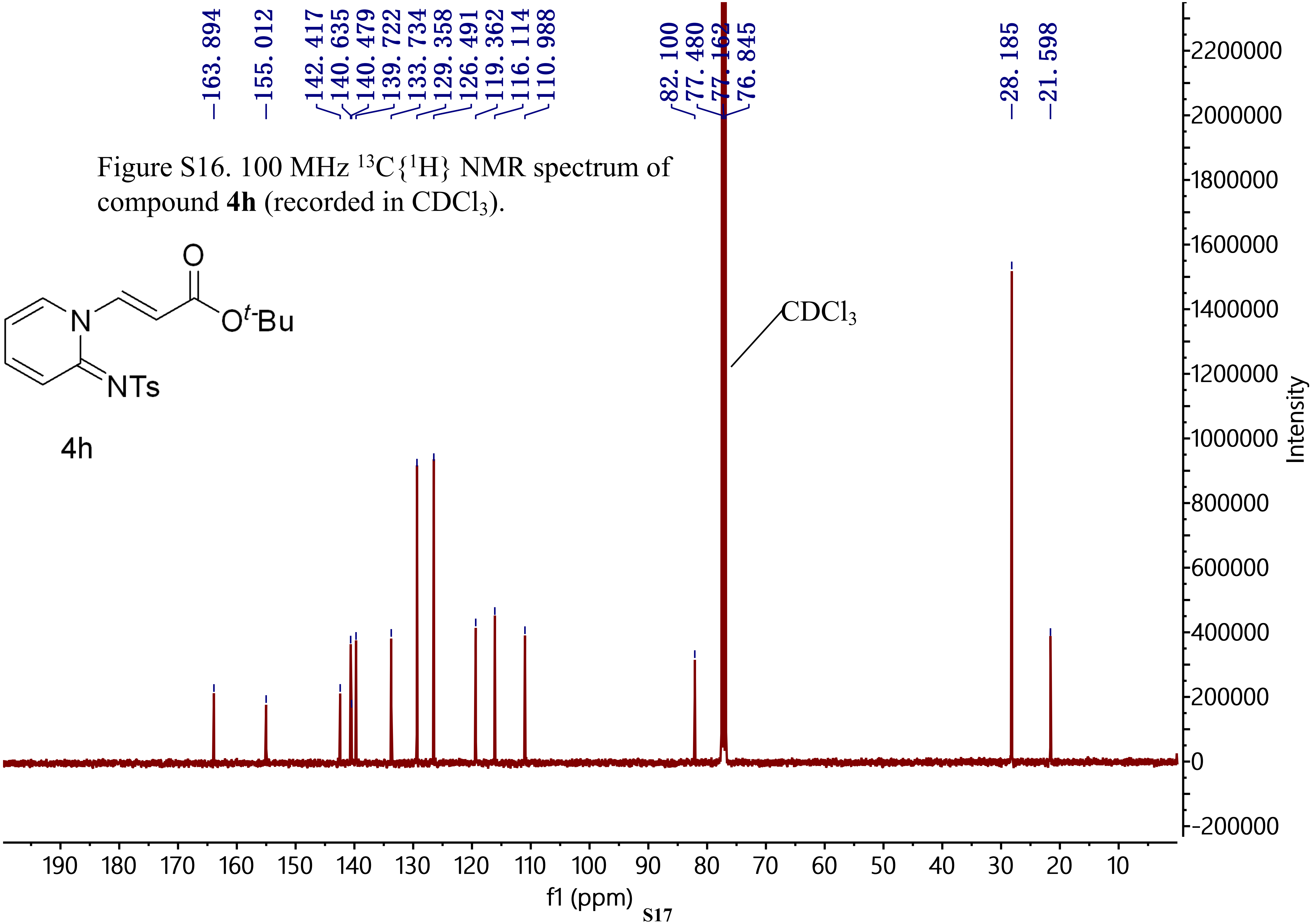

4h

Figure S16. 100 MHz $^{13}C\{^1H\}$ NMR spectrum of compound **4h** (recorded in $CDCl_3$).

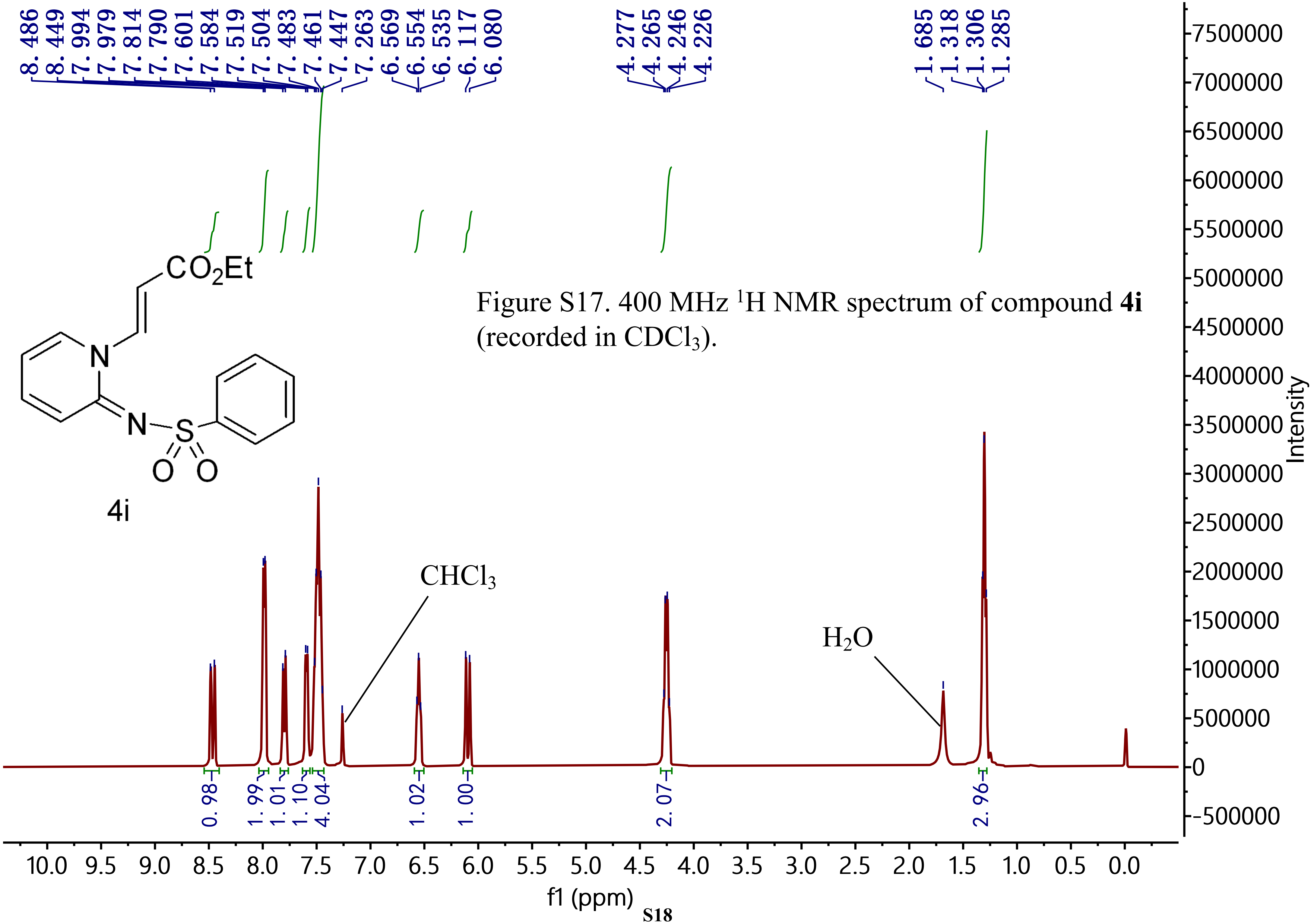

Figure S17. 400 MHz ^{1}H NMR spectrum of compound **4i** (recorded in $CDCl_3$).

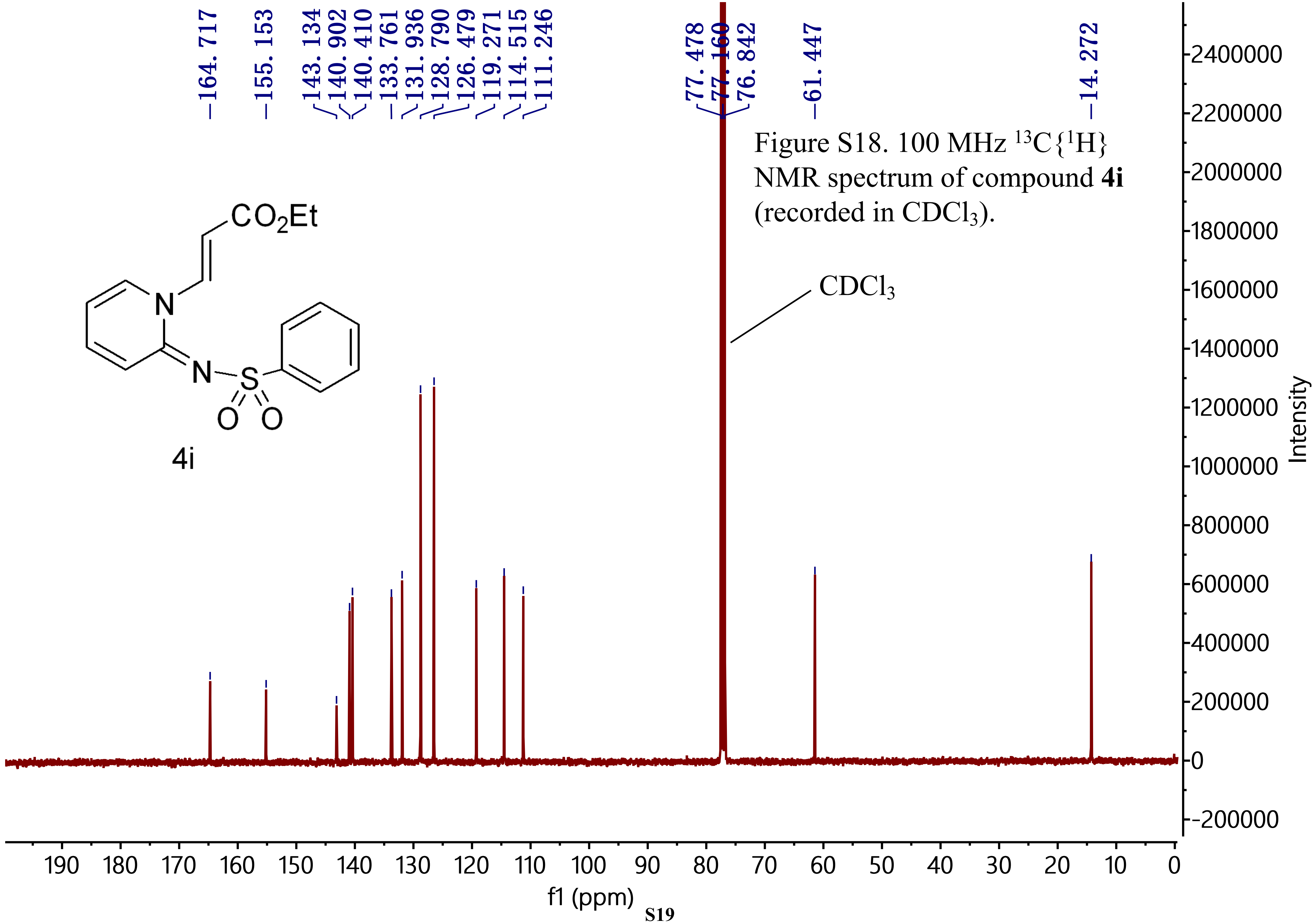

Figure S18. 100 MHz $^{13}C\{^{1}H\}$ NMR spectrum of compound **4i** (recorded in $CDCl_3$).

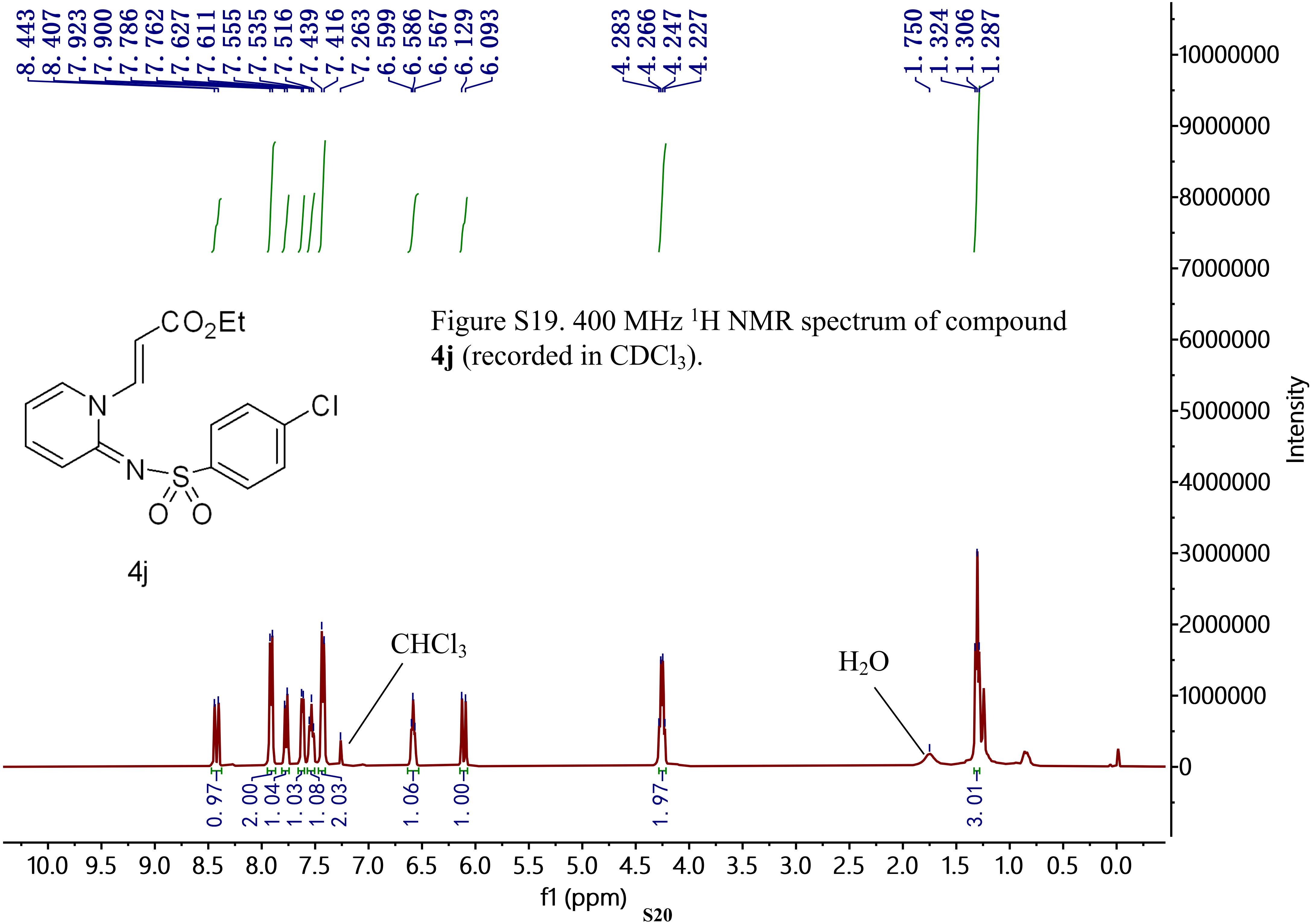

4j

Figure S19. 400 MHz ^{1}H NMR spectrum of compound **4j** (recorded in $CDCl_3$).

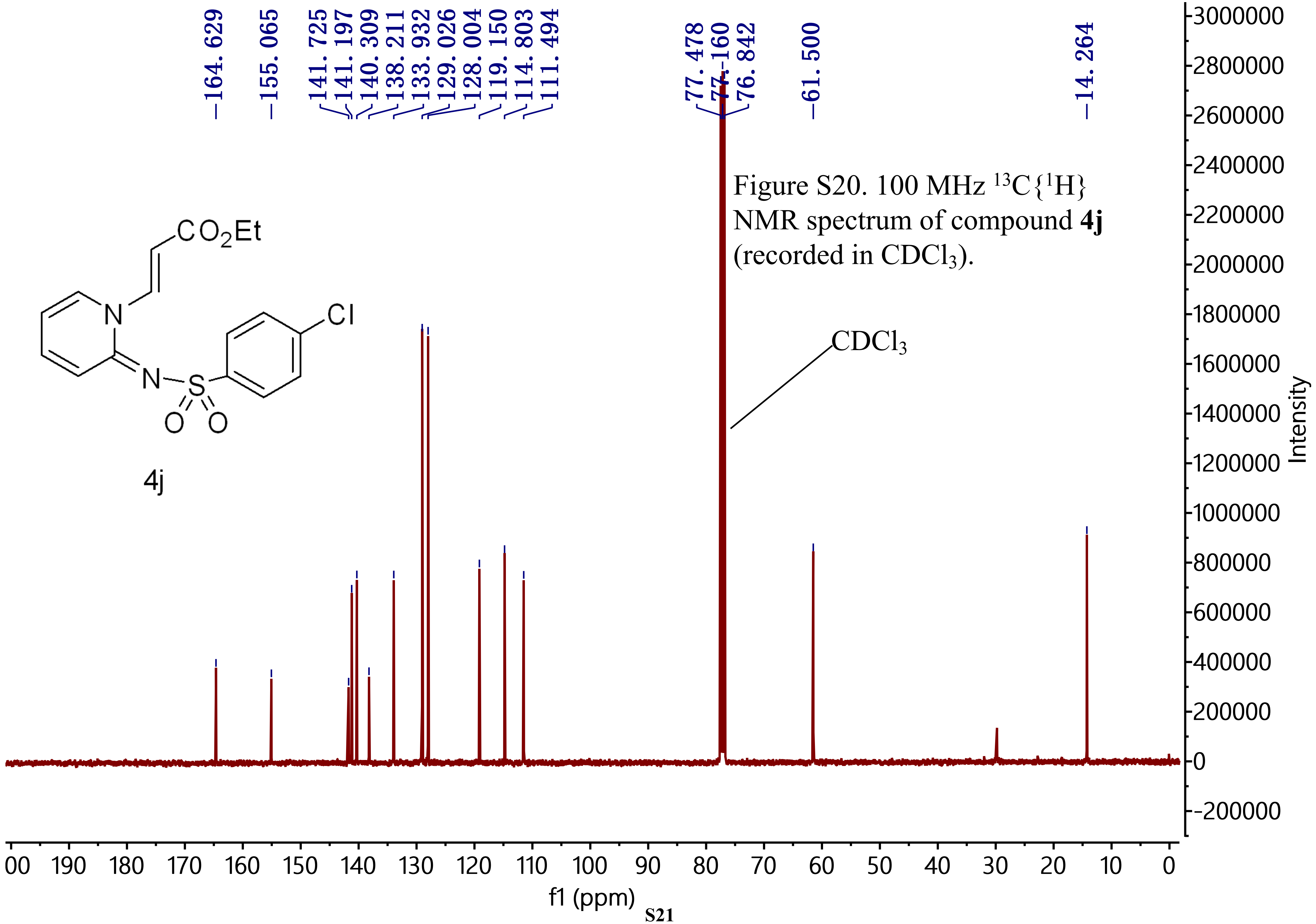

Figure S20. 100 MHz $^{13}C\{^{1}H\}$ NMR spectrum of compound **4j** (recorded in $CDCl_3$).

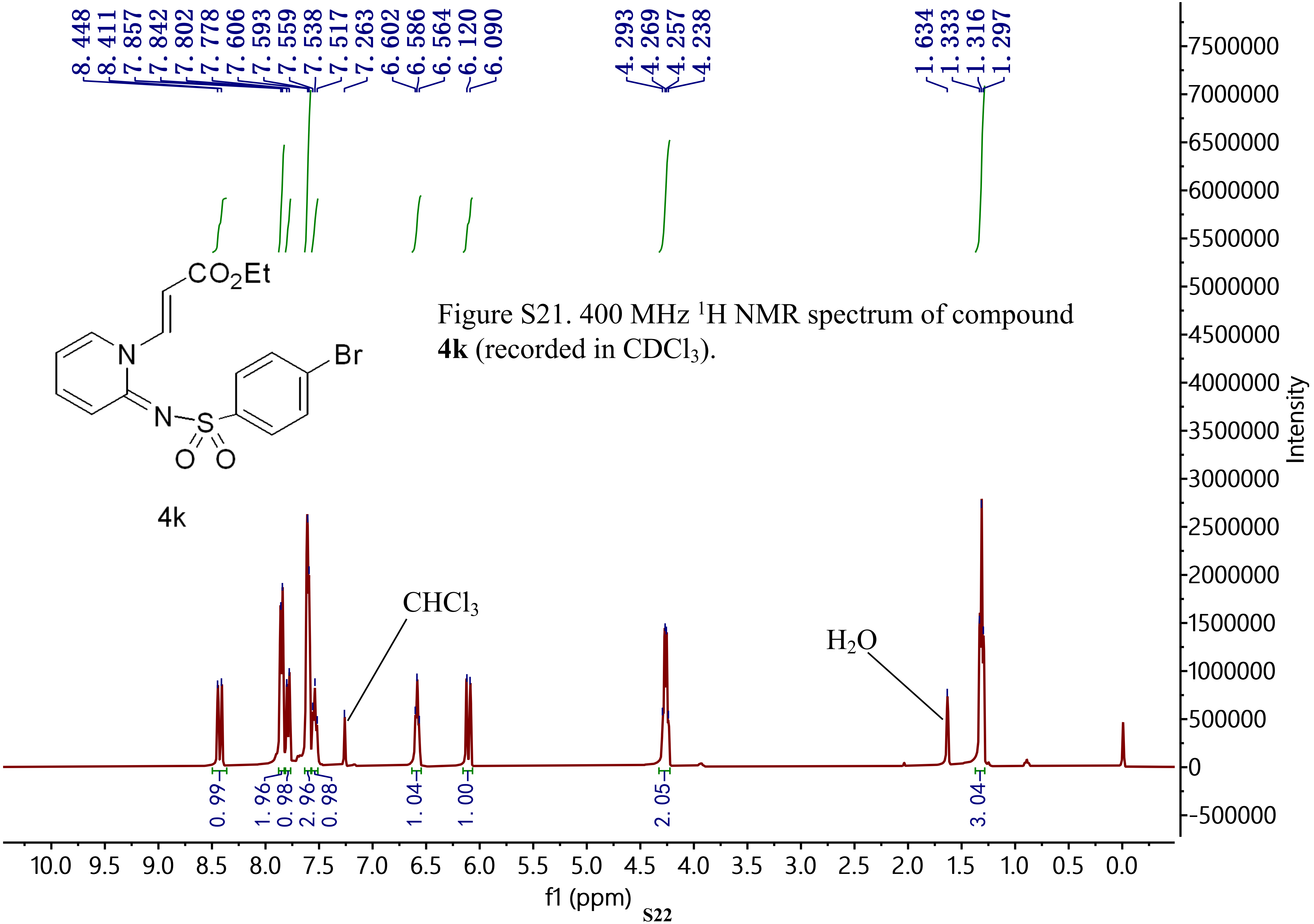

Figure S21. 400 MHz ^{1}H NMR spectrum of compound **4k** (recorded in $CDCl_3$).

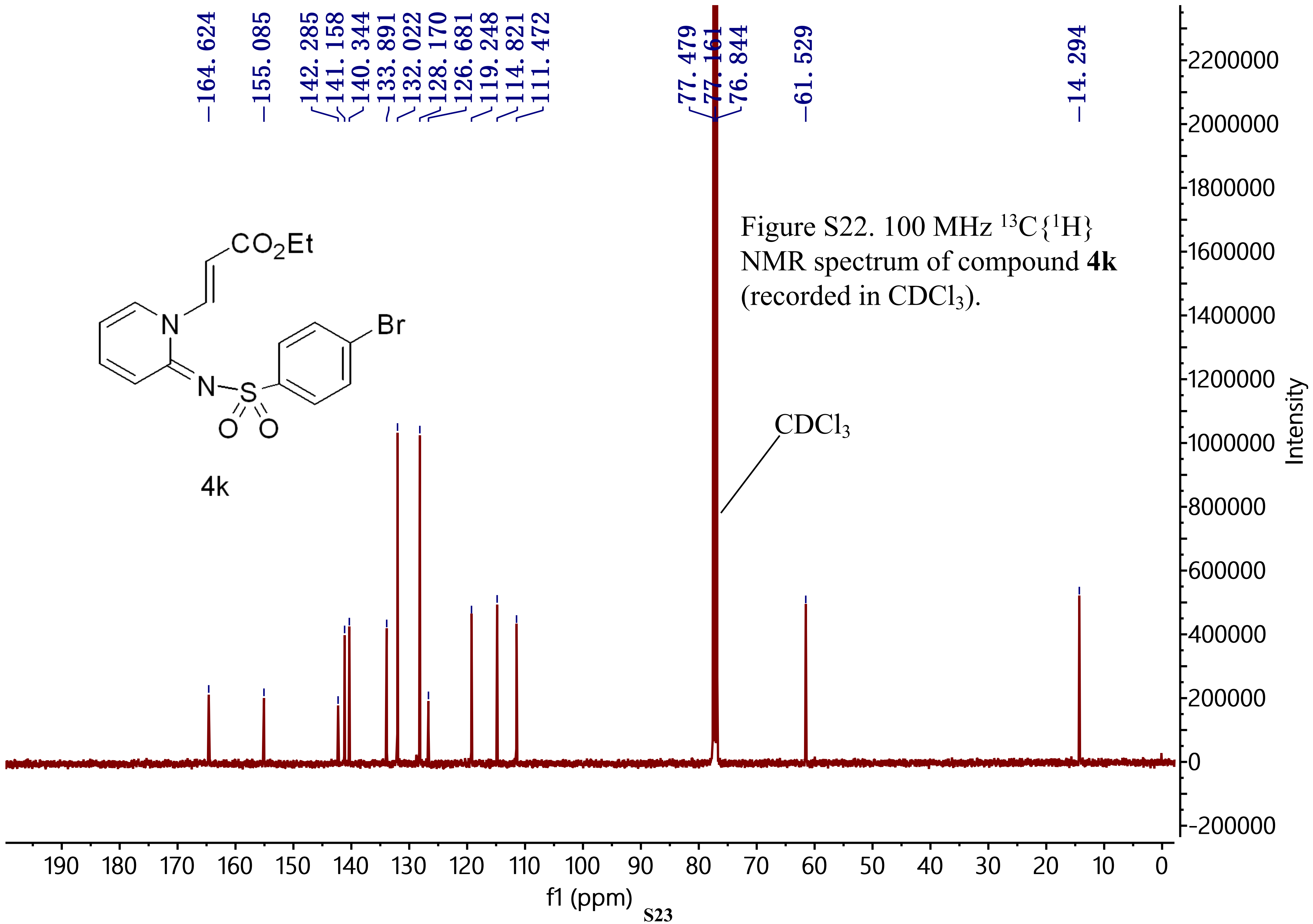

Figure S22. 100 MHz $^{13}C\{^{1}H\}$ NMR spectrum of compound **4k** (recorded in $CDCl_3$).

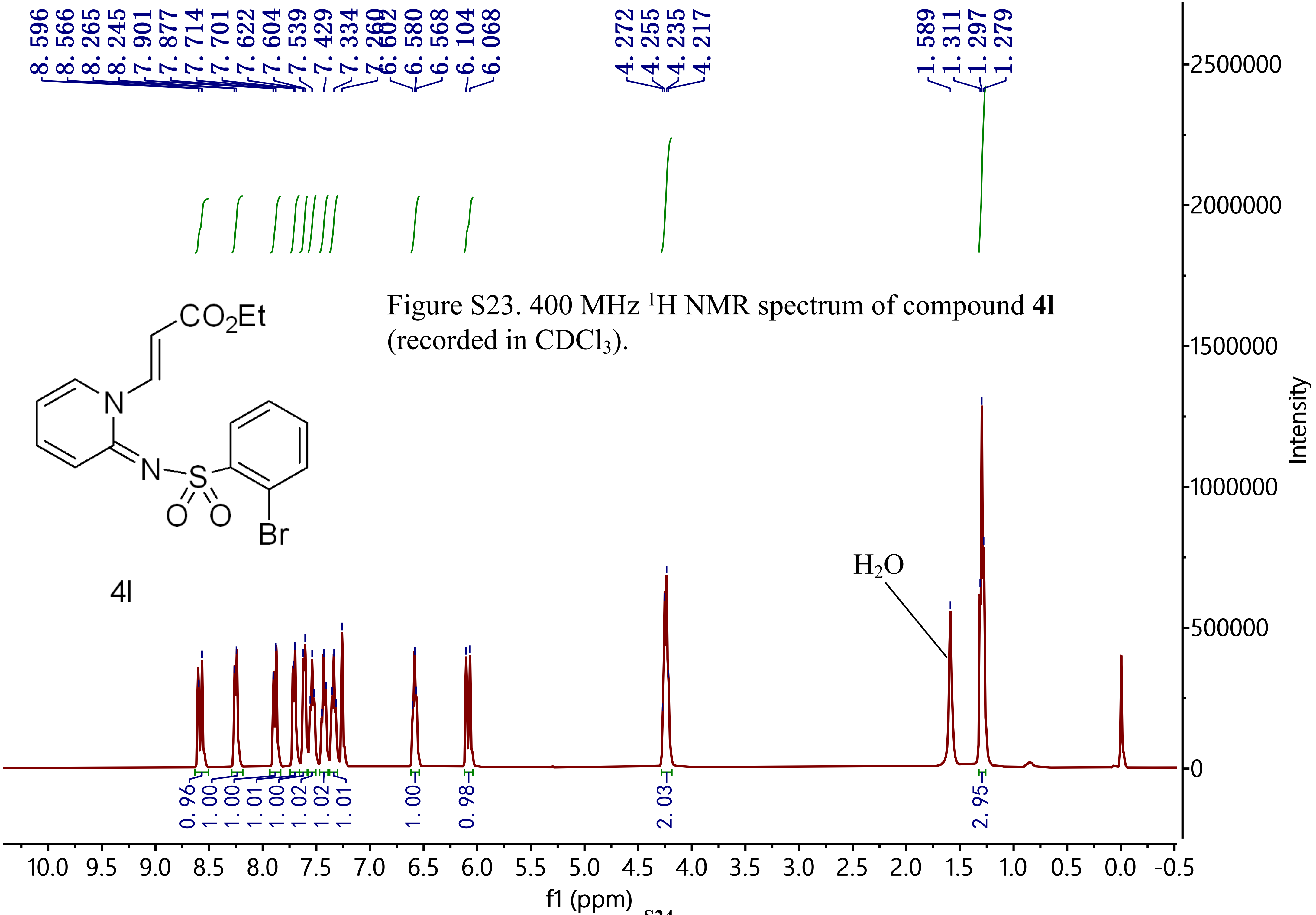

4l

Figure S23. 400 MHz ^{1}H NMR spectrum of compound **4l** (recorded in $CDCl_3$).

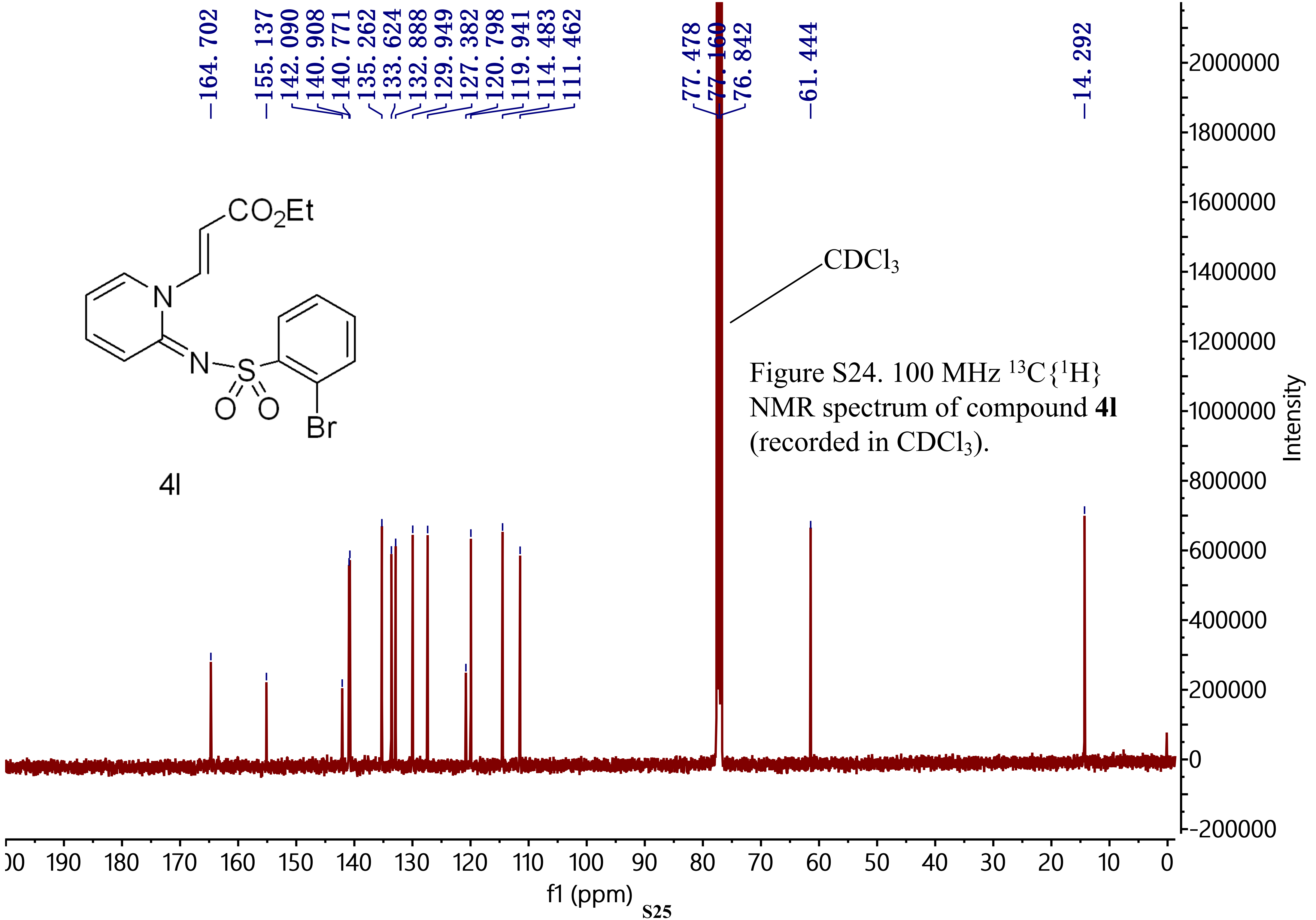

Figure S24. 100 MHz $^{13}C\{^{1}H\}$ NMR spectrum of compound **4l** (recorded in $CDCl_3$).

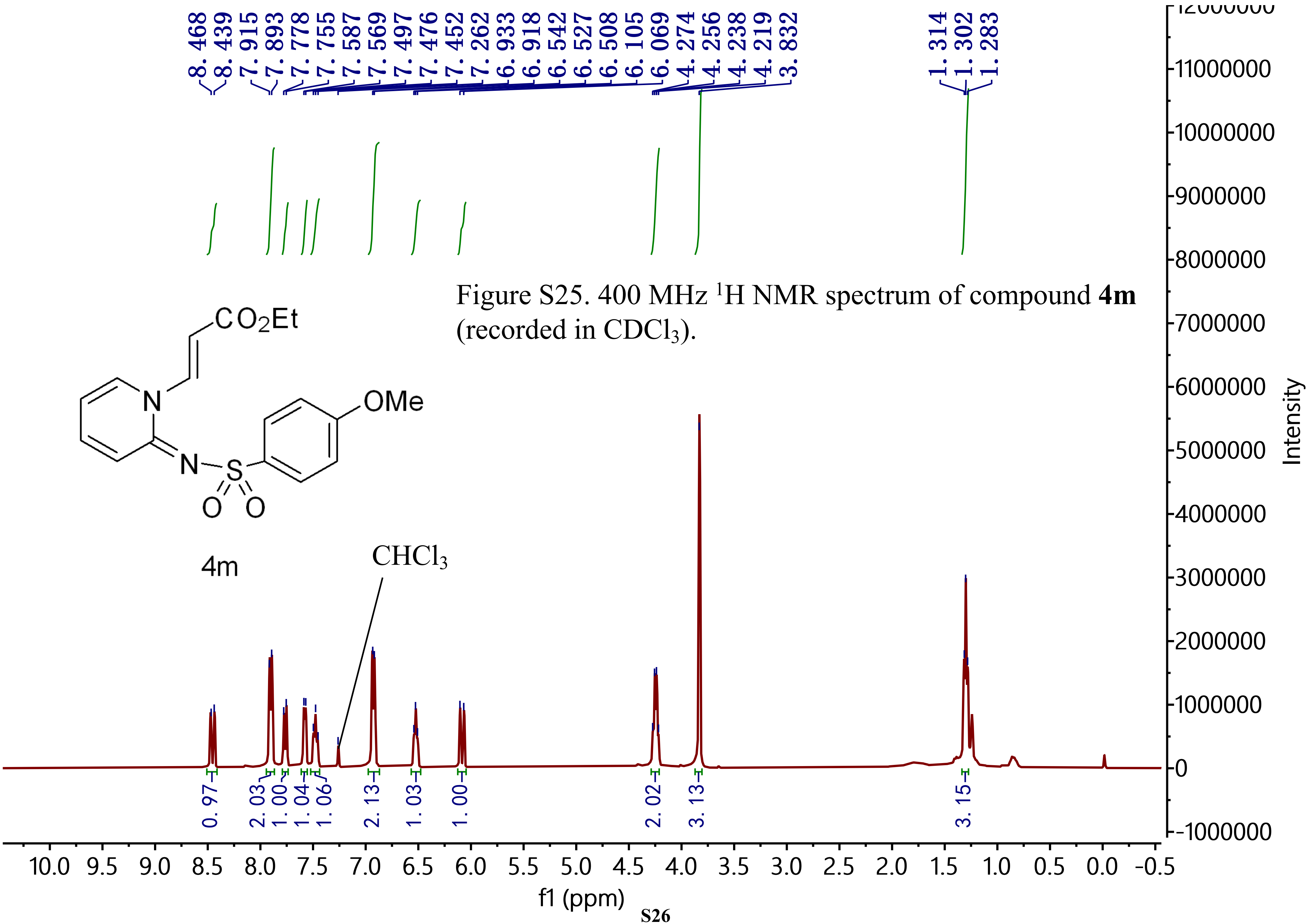

Figure S25. 400 MHz ^{1}H NMR spectrum of compound **4m** (recorded in $CDCl_3$).

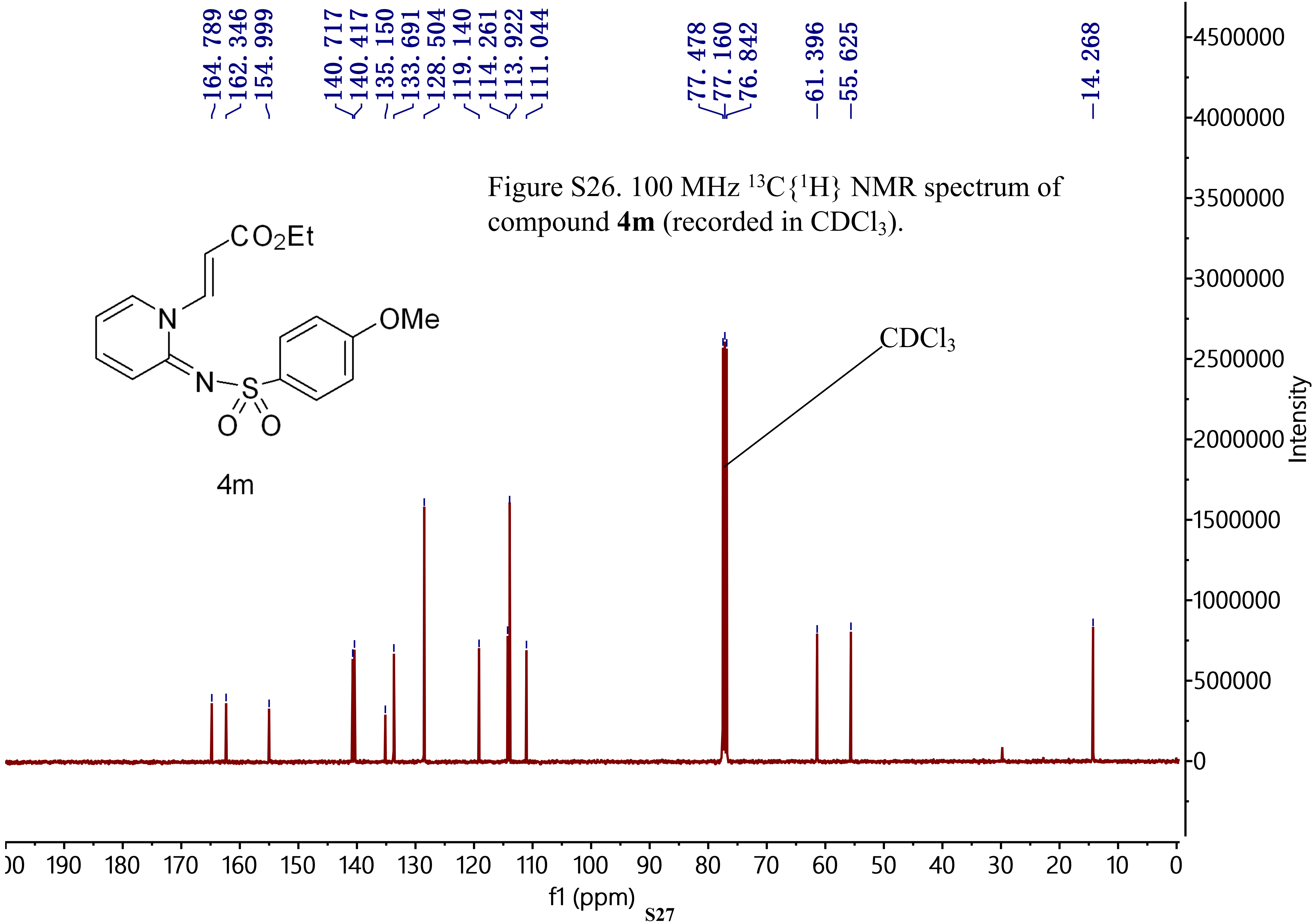

Figure S26. 100 MHz $^{13}C\{^{1}H\}$ NMR spectrum of compound **4m** (recorded in $CDCl_3$).

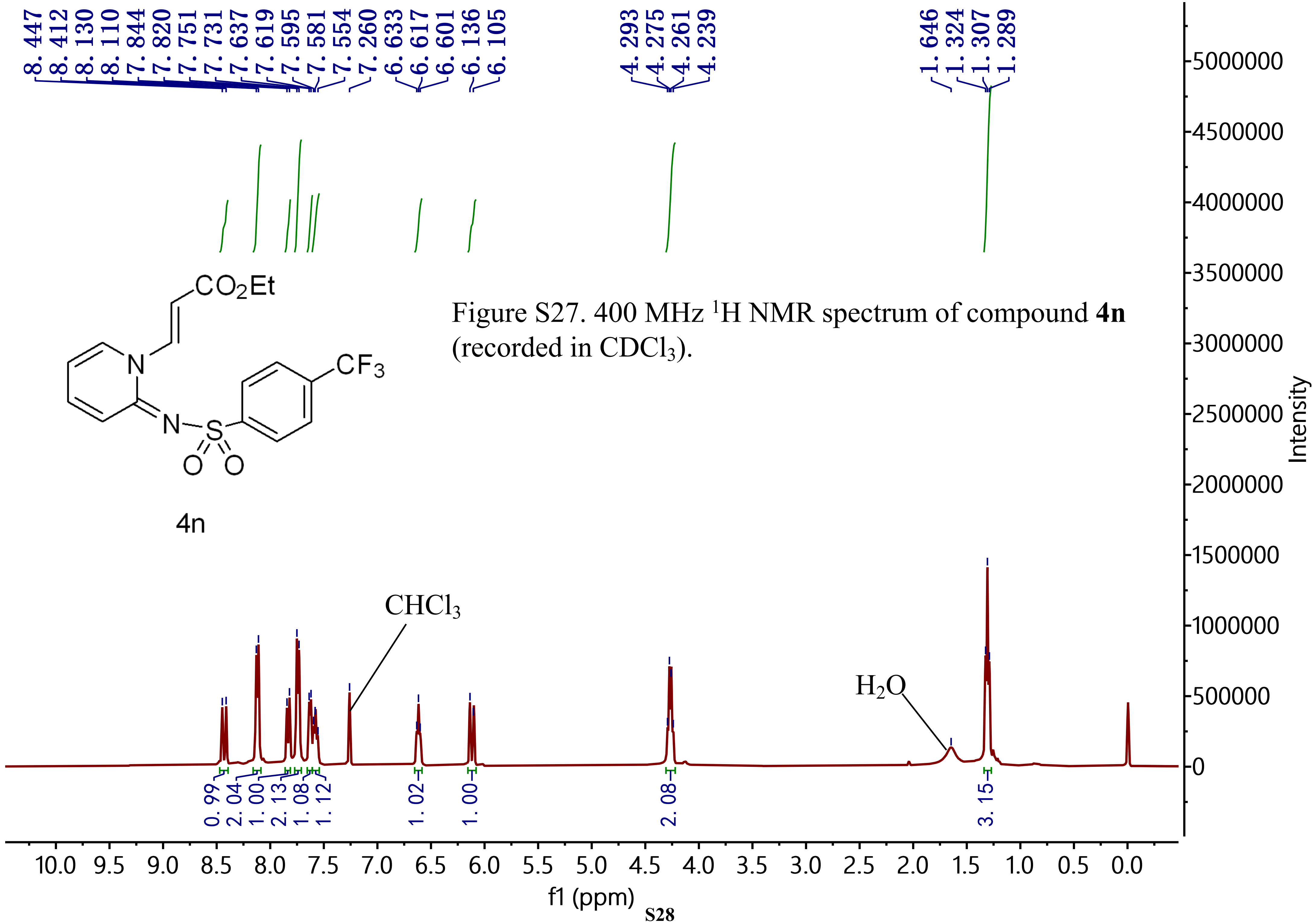

Figure S27. 400 MHz ^{1}H NMR spectrum of compound **4n** (recorded in $CDCl_3$).

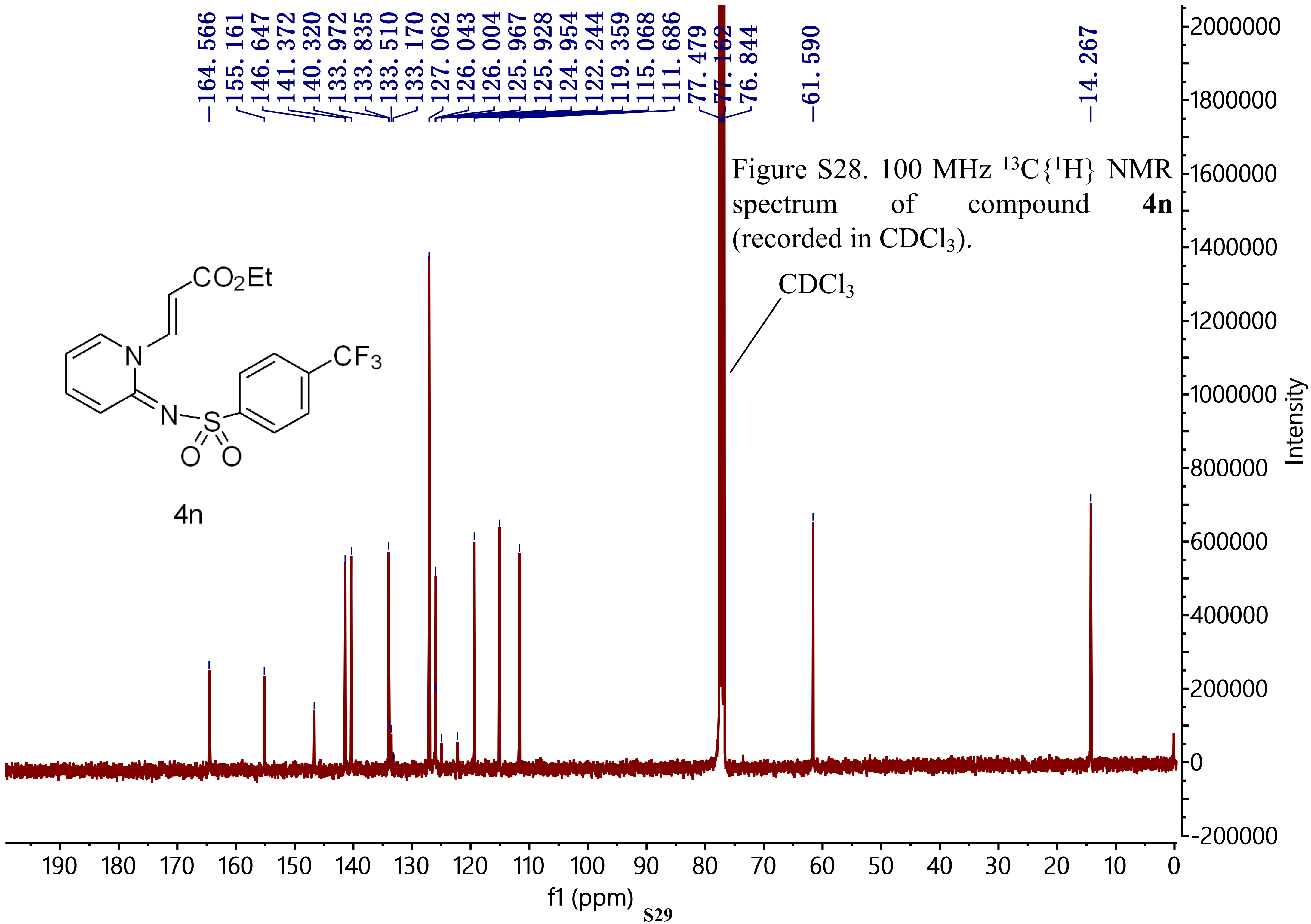

Figure S28. 100 MHz $^{13}C\{^1H\}$ NMR spectrum of compound **4n** (recorded in $CDCl_3$).

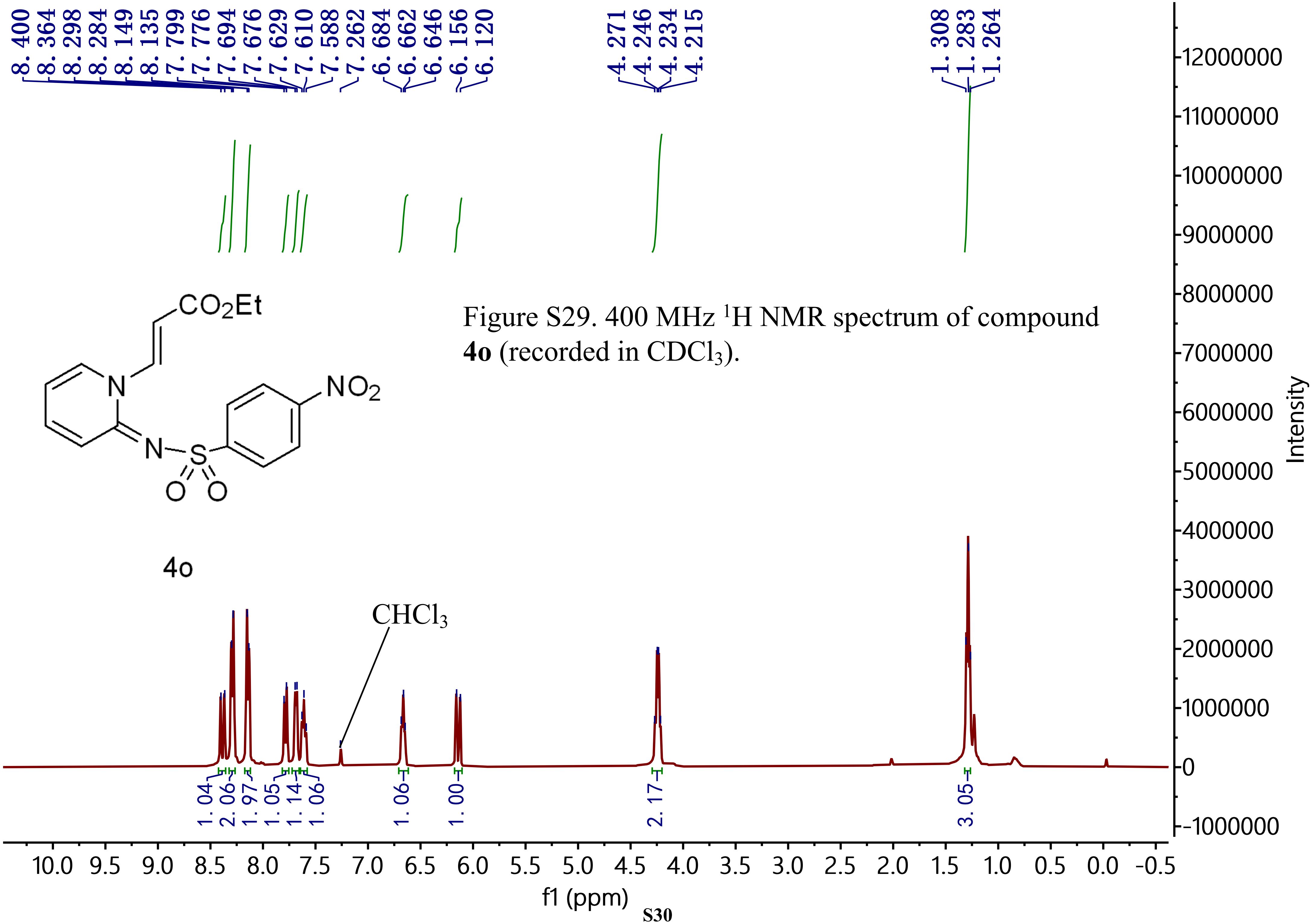

Figure S29. 400 MHz ^{1}H NMR spectrum of compound **4o** (recorded in $CDCl_3$).

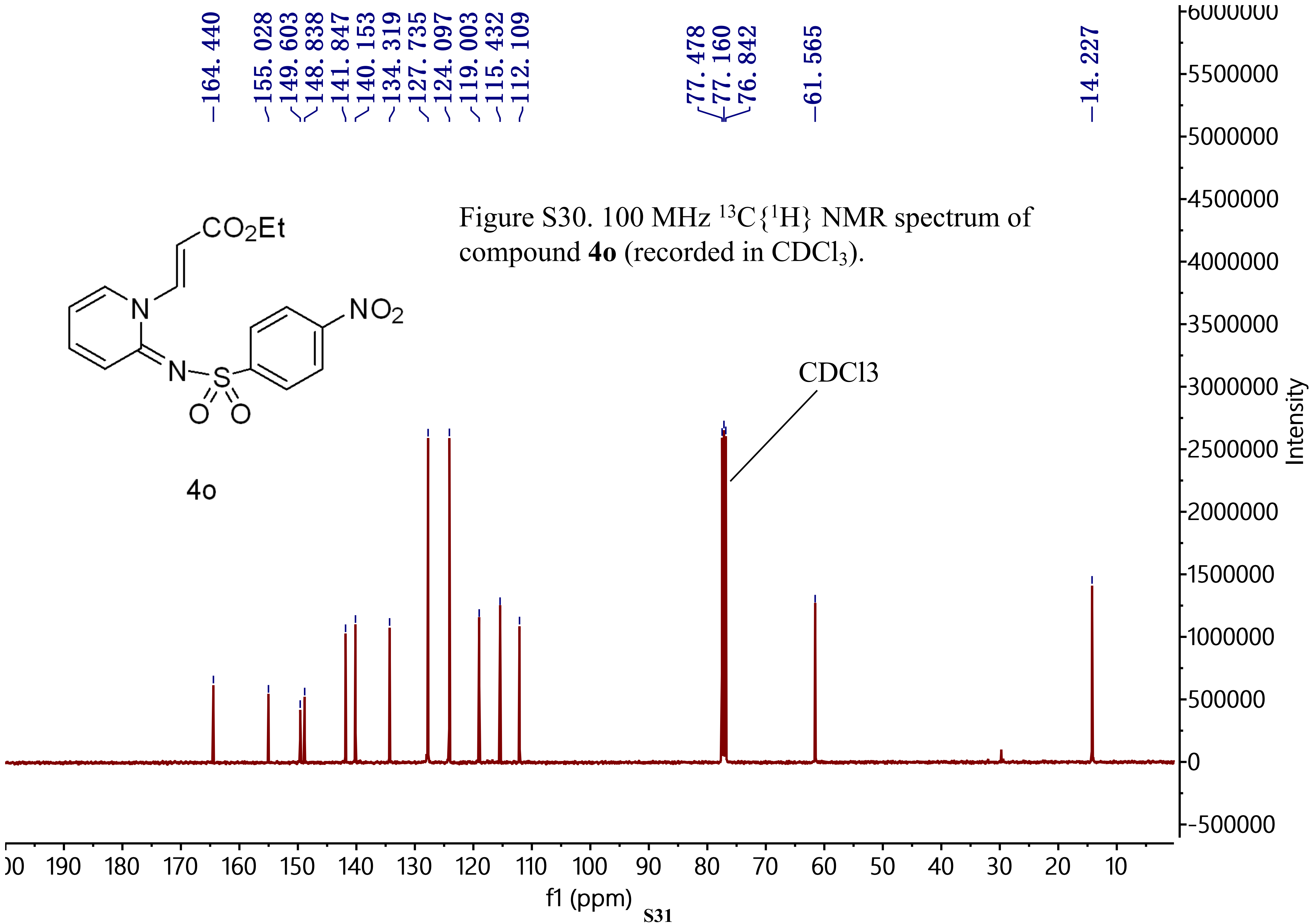

Figure S30. 100 MHz $^{13}C\{^{1}H\}$ NMR spectrum of compound **4o** (recorded in $CDCl_3$).

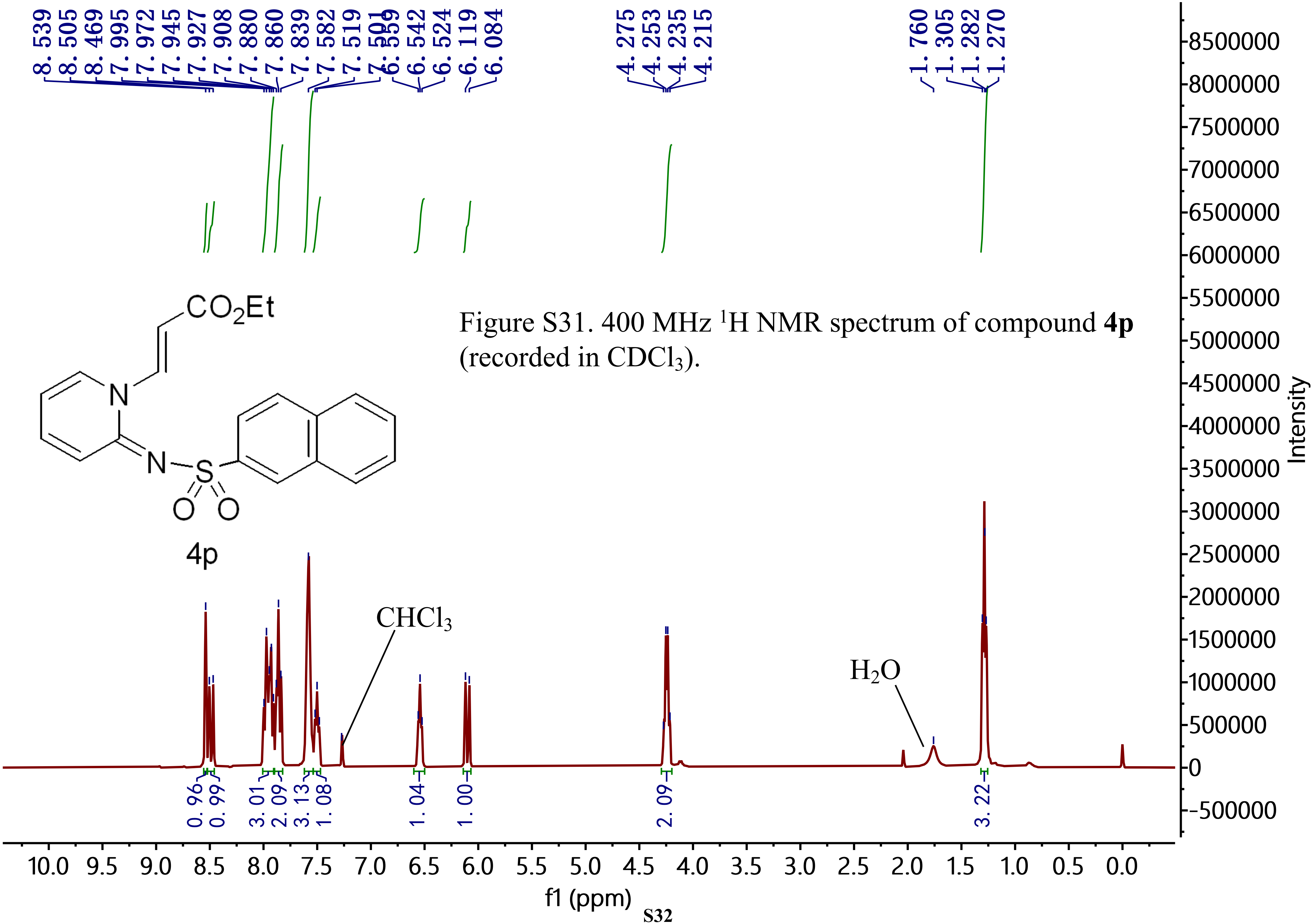

Figure S31. 400 MHz ^{1}H NMR spectrum of compound **4p** (recorded in $CDCl_3$).

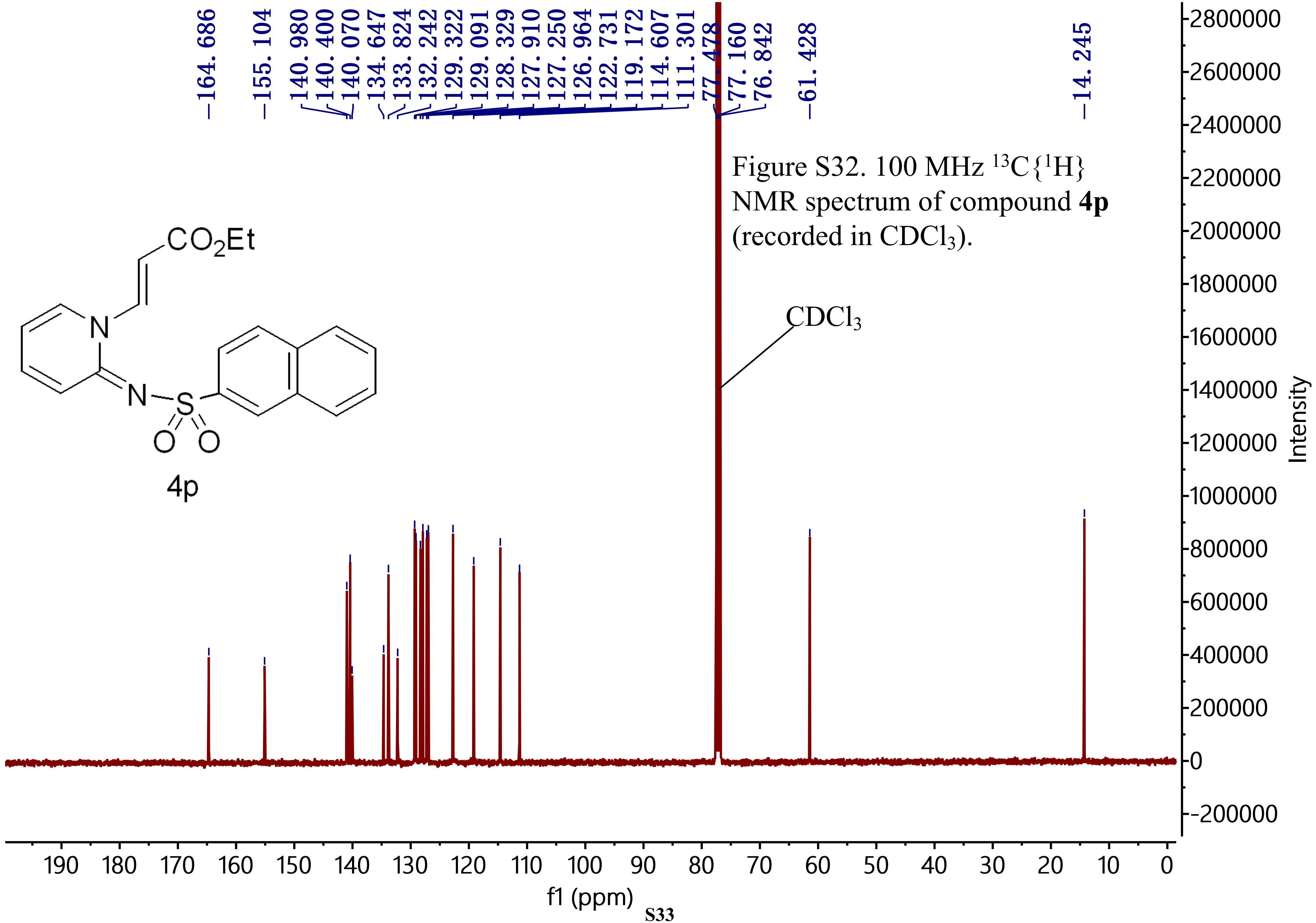

Figure S32. 100 MHz $^{13}C\{^{1}H\}$ NMR spectrum of compound **4p** (recorded in $CDCl_3$).

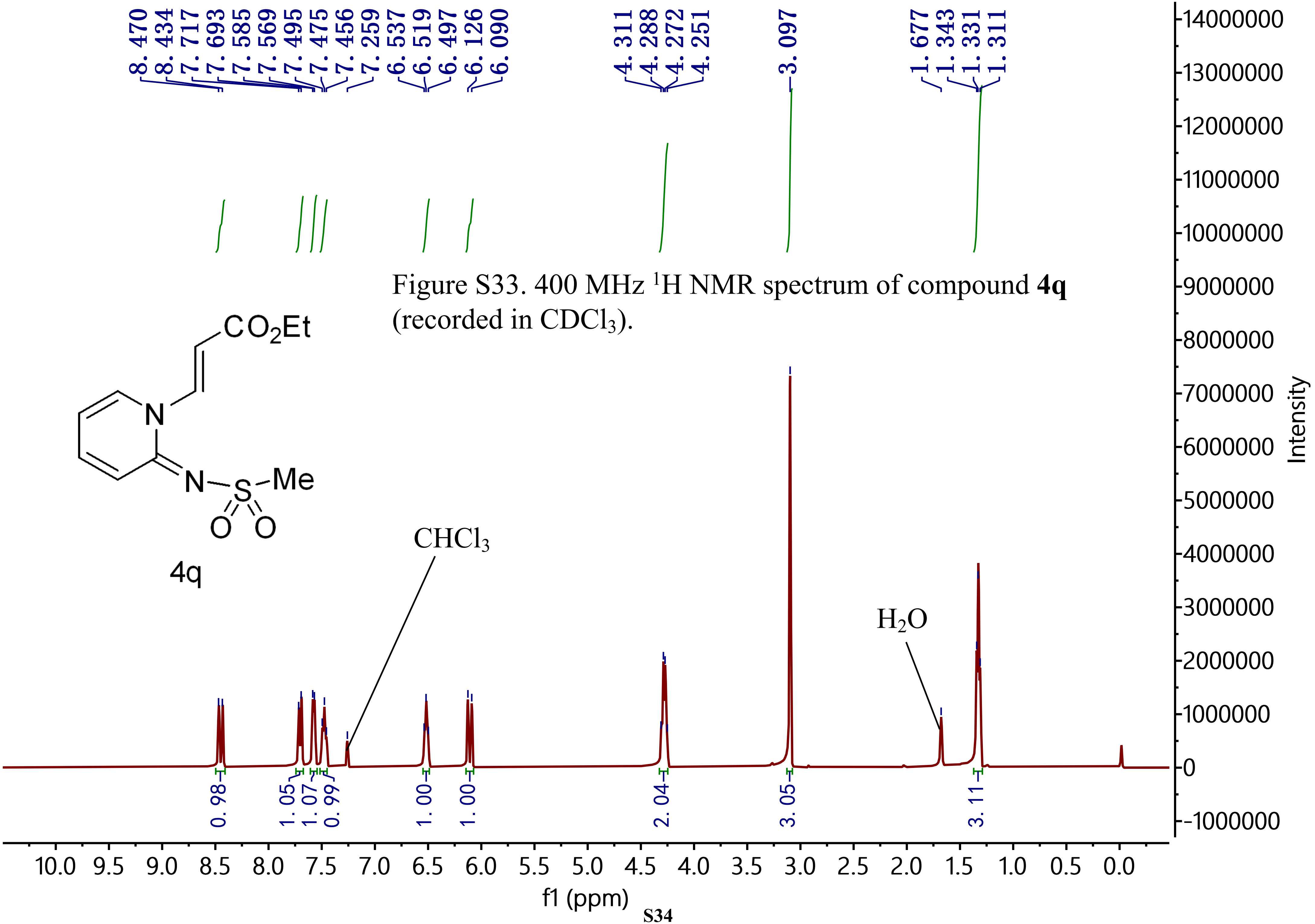

Figure S33. 400 MHz ^1H NMR spectrum of compound **4q** (recorded in $CDCl_3$).

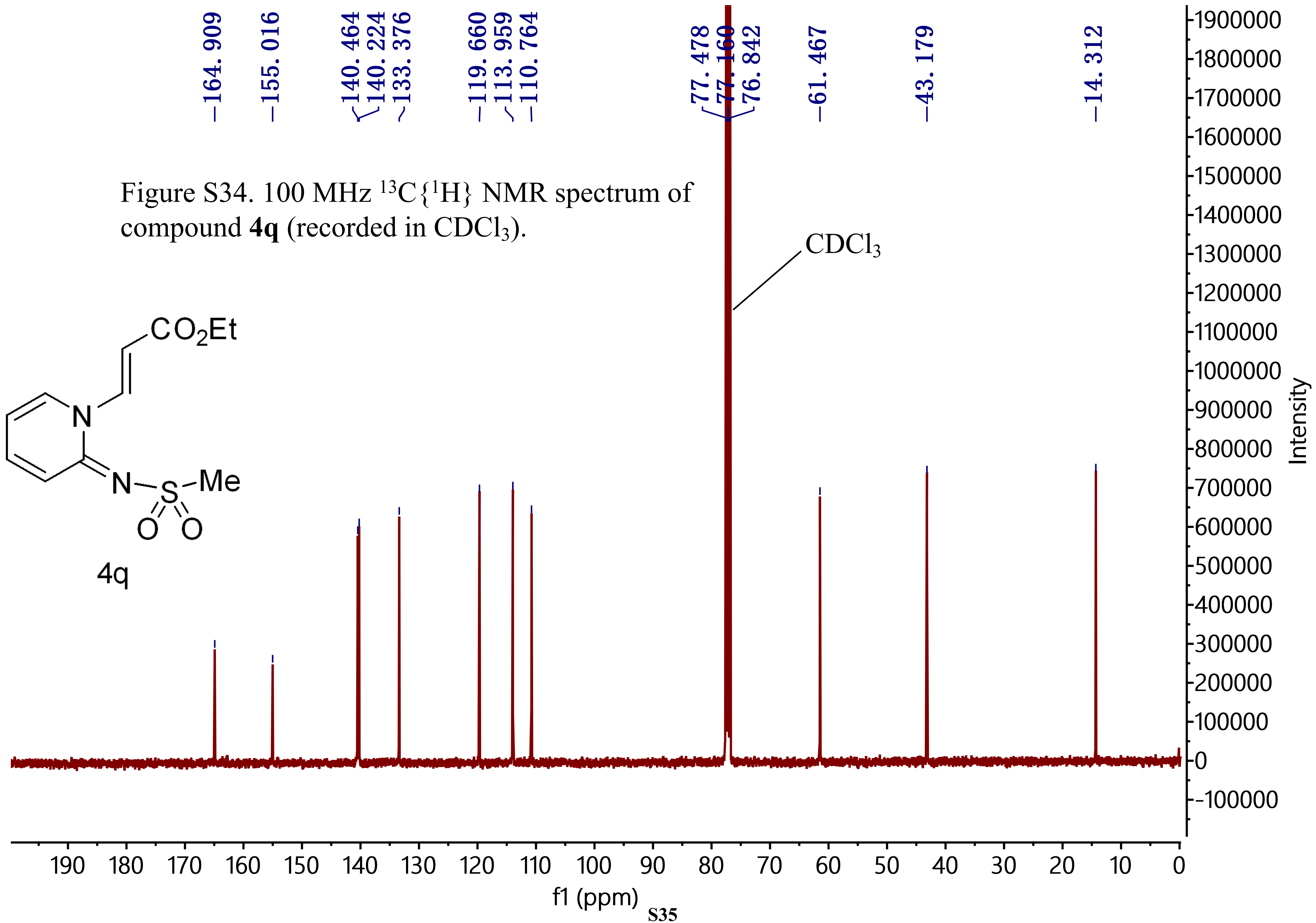

4q

Figure S34. 100 MHz $^{13}C\{^1H\}$ NMR spectrum of compound **4q** (recorded in $CDCl_3$).

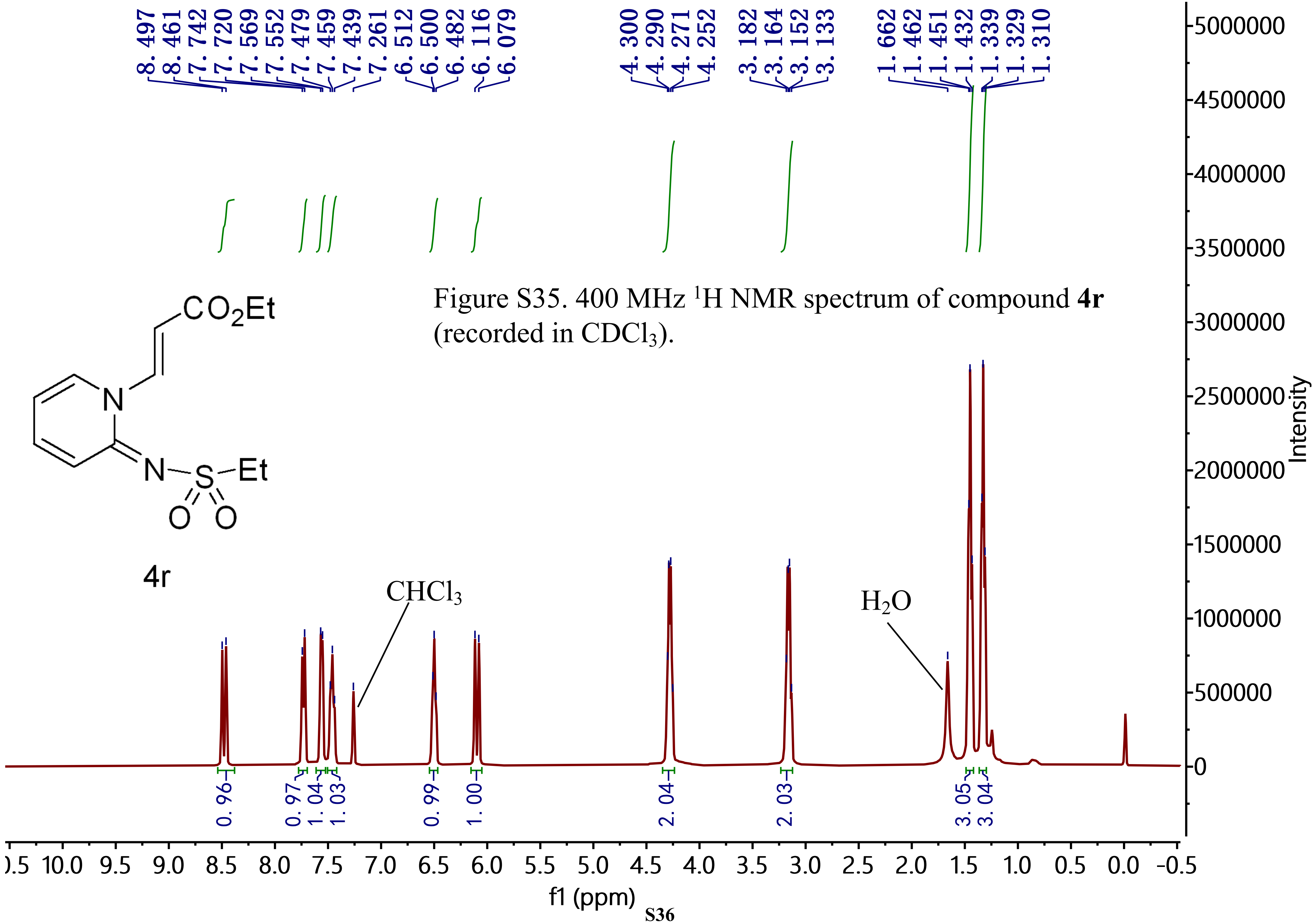

Figure S35. 400 MHz ^{1}H NMR spectrum of compound **4r** (recorded in $CDCl_3$).

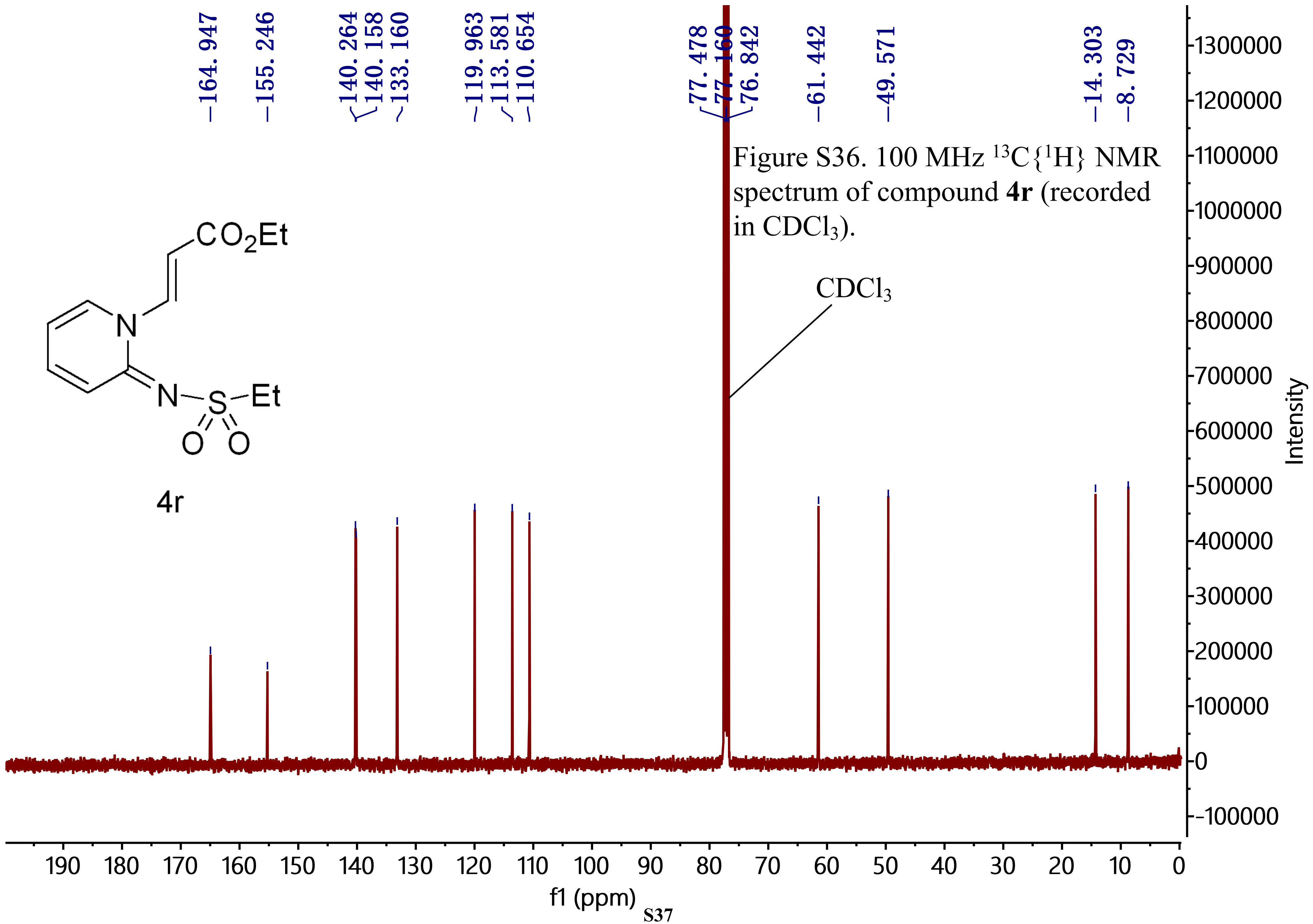

Figure S36. 100 MHz $^{13}C\{^{1}H\}$ NMR spectrum of compound **4r** (recorded in $CDCl_3$).

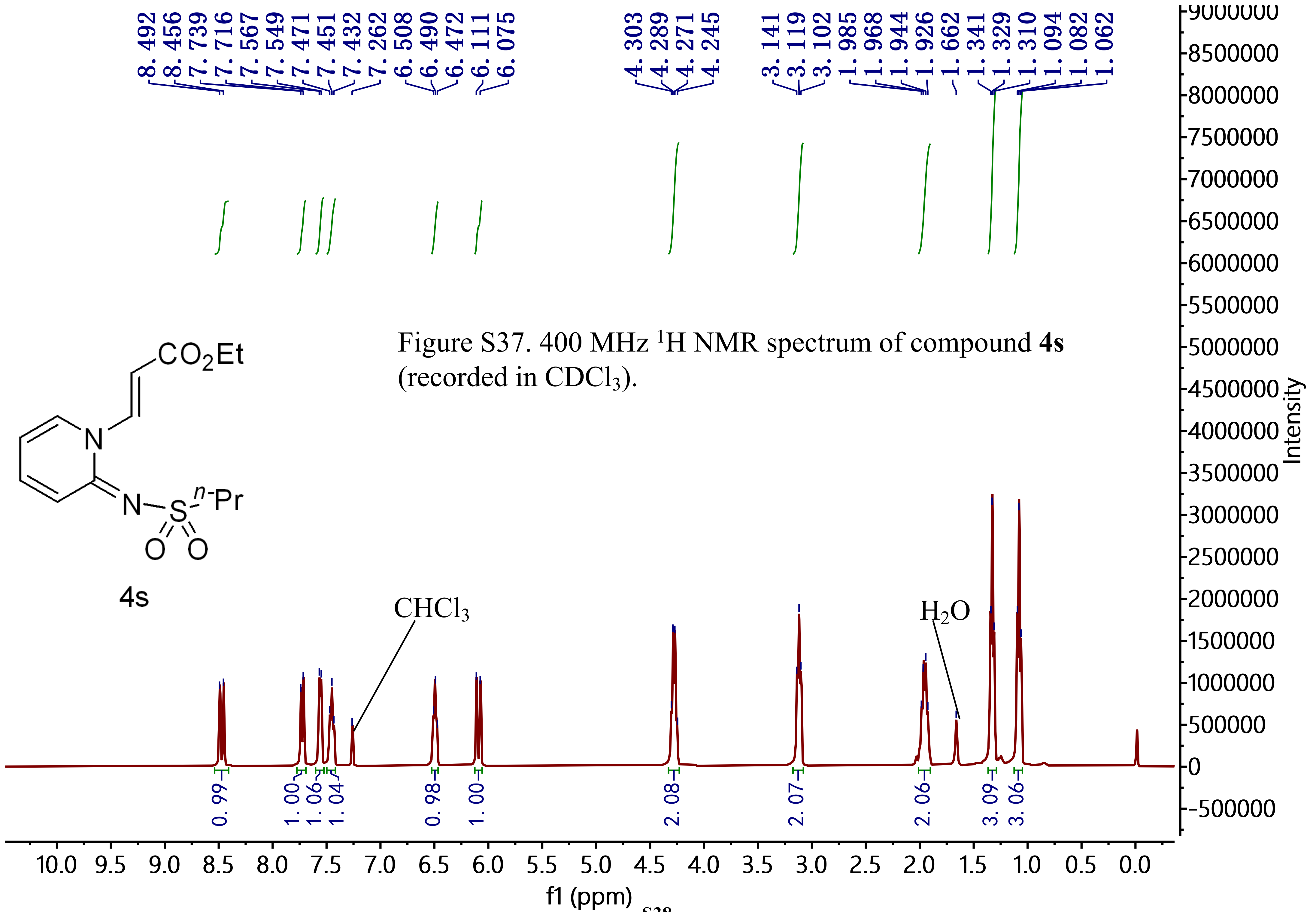

Figure S37. 400 MHz ¹H NMR spectrum of compound **4s** (recorded in $CDCl_3$).

Figure S38. 100 MHz $^{13}C\{^{1}H\}$ NMR spectrum of compound **4s** (recorded in $CDCl_3$).

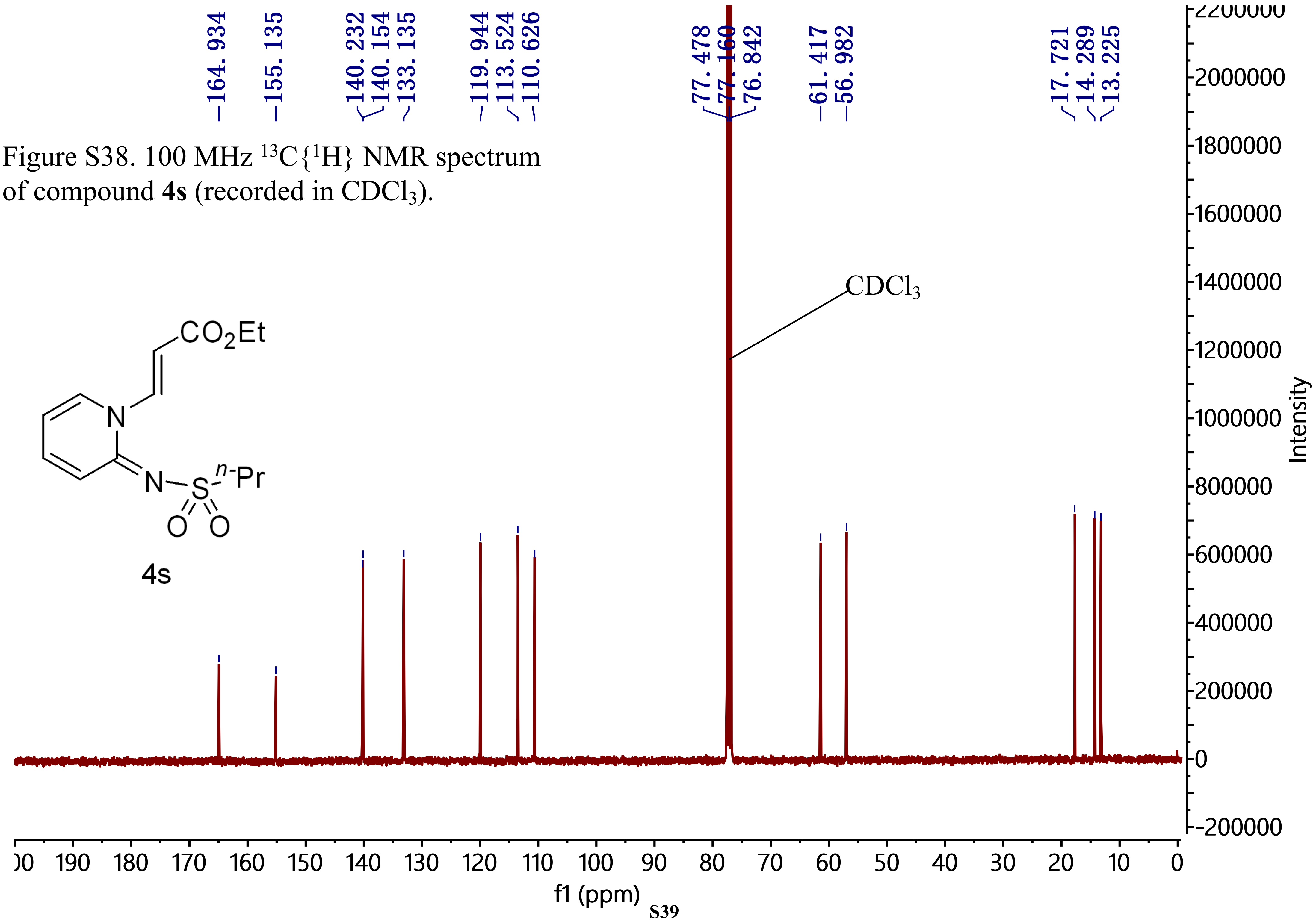

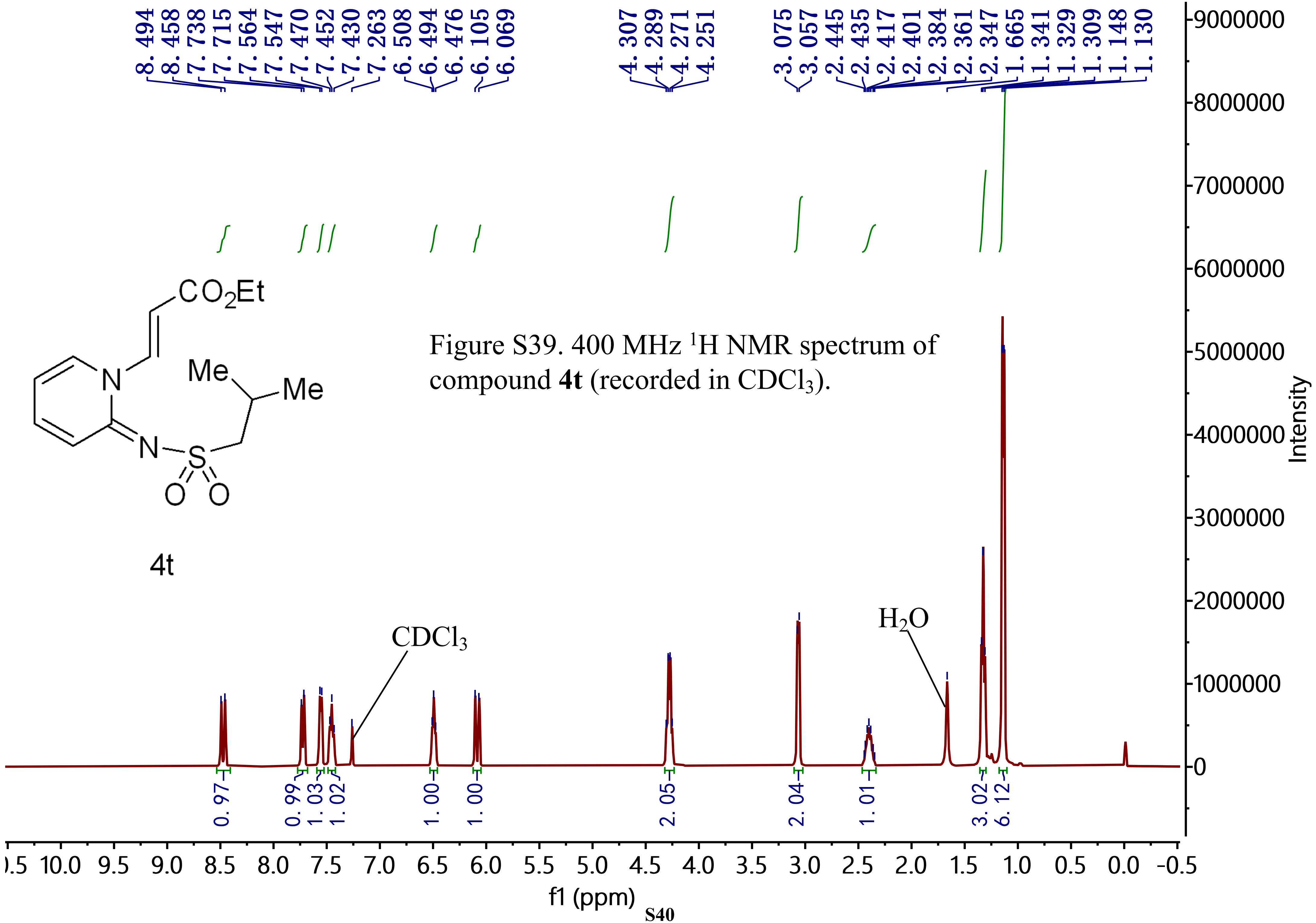

Figure S39. 400 MHz ^{1}H NMR spectrum of compound **4t** (recorded in $CDCl_3$).

Figure S40. 100 MHz $^{13}C\{^1H\}$ NMR spectrum of compound **4t** (recorded in $CDCl_3$).

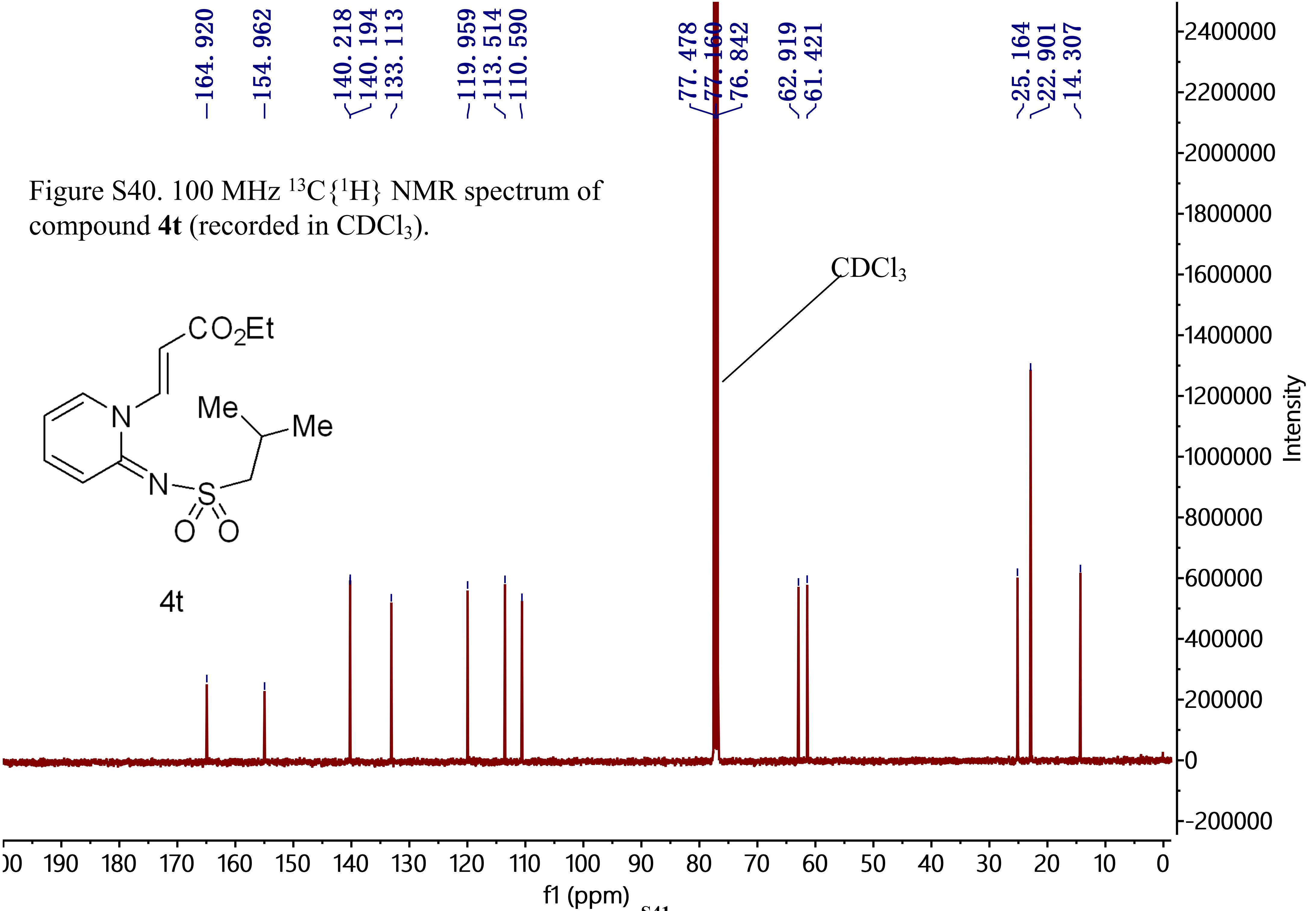

4t

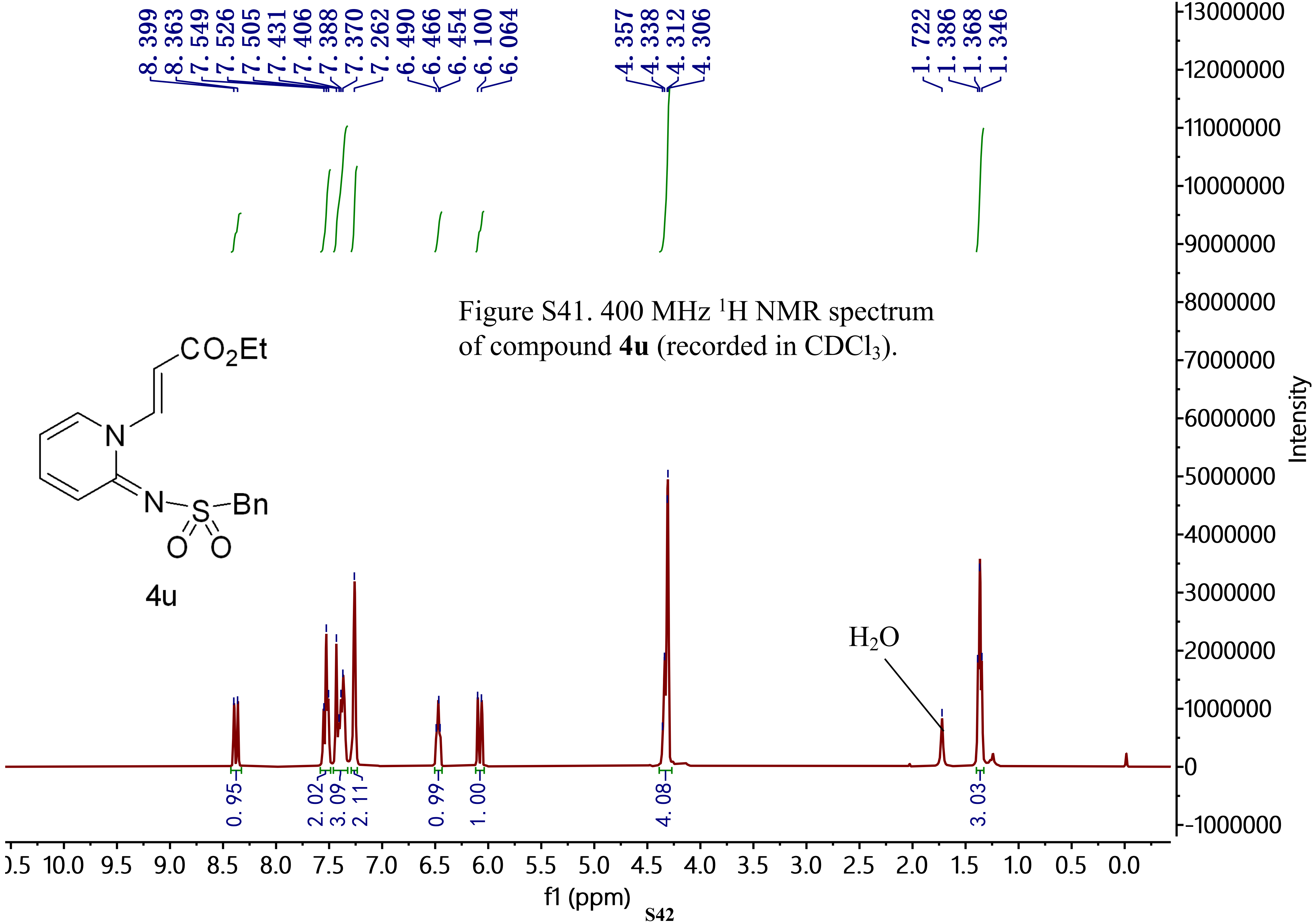

Figure S41. 400 MHz ^{1}H NMR spectrum of compound **4u** (recorded in $CDCl_3$).

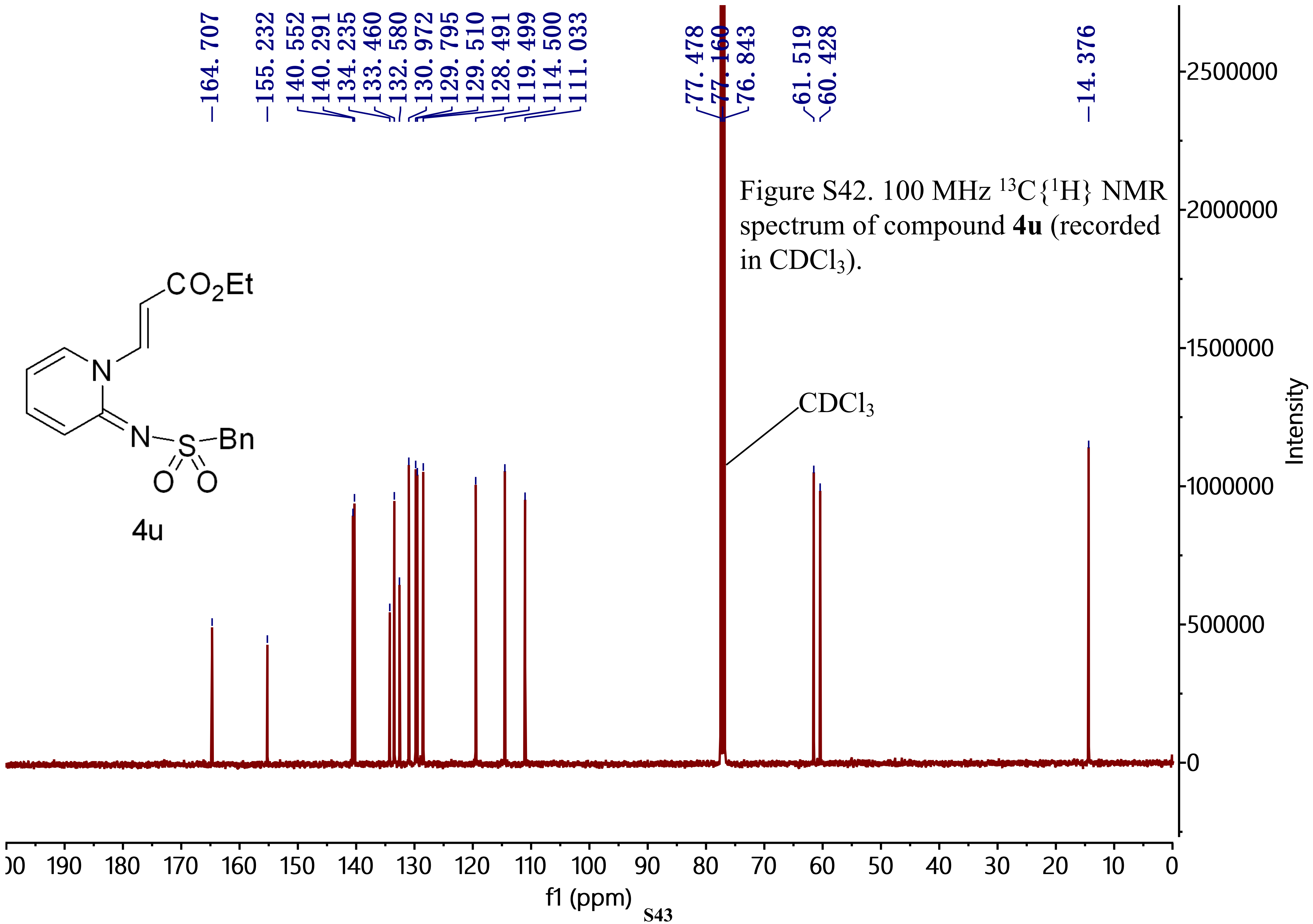

Figure S42. 100 MHz $^{13}C\{^{1}H\}$ NMR spectrum of compound **4u** (recorded in $CDCl_3$).

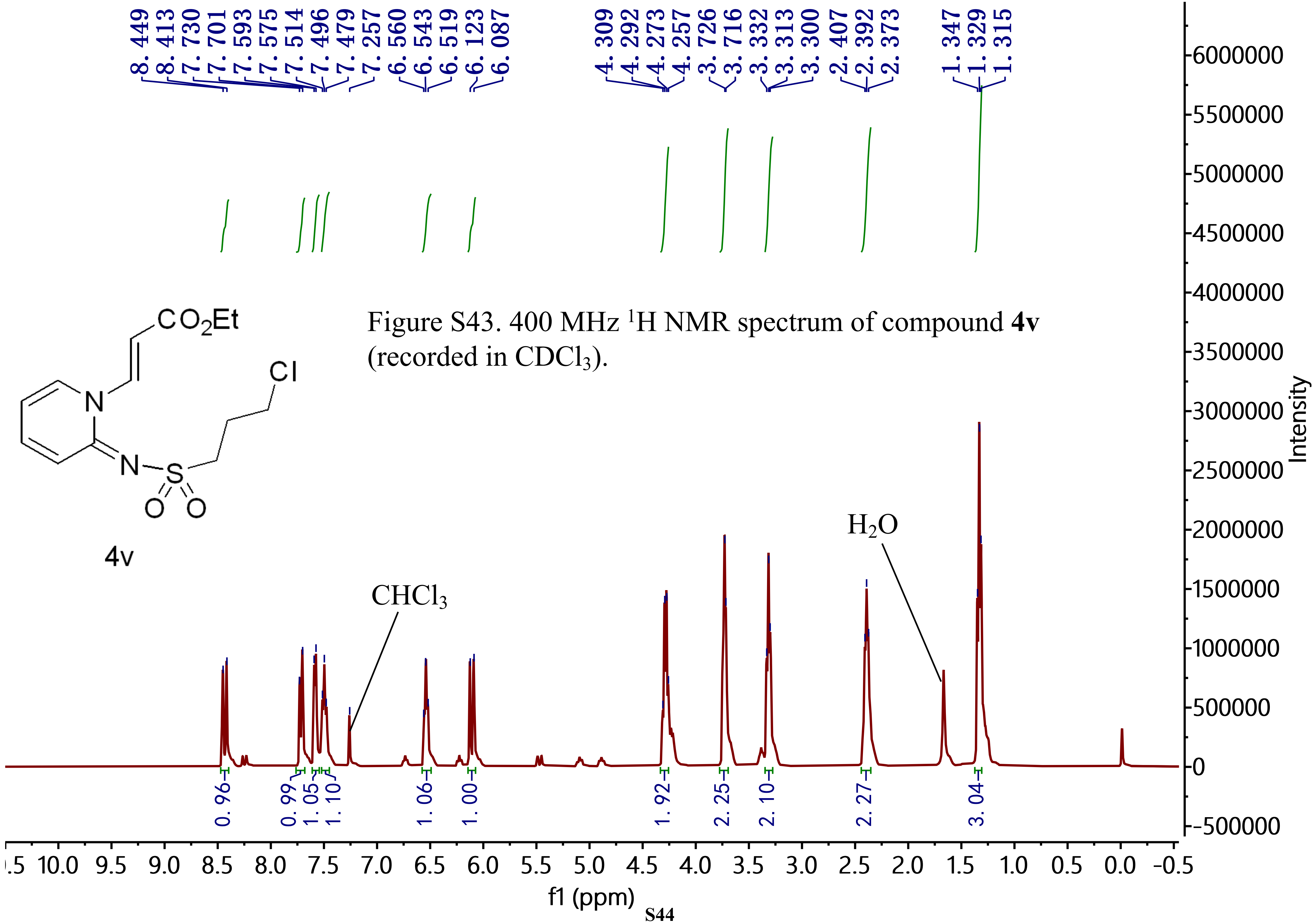

Figure S43. 400 MHz ^{1}H NMR spectrum of compound **4v** (recorded in $CDCl_3$).

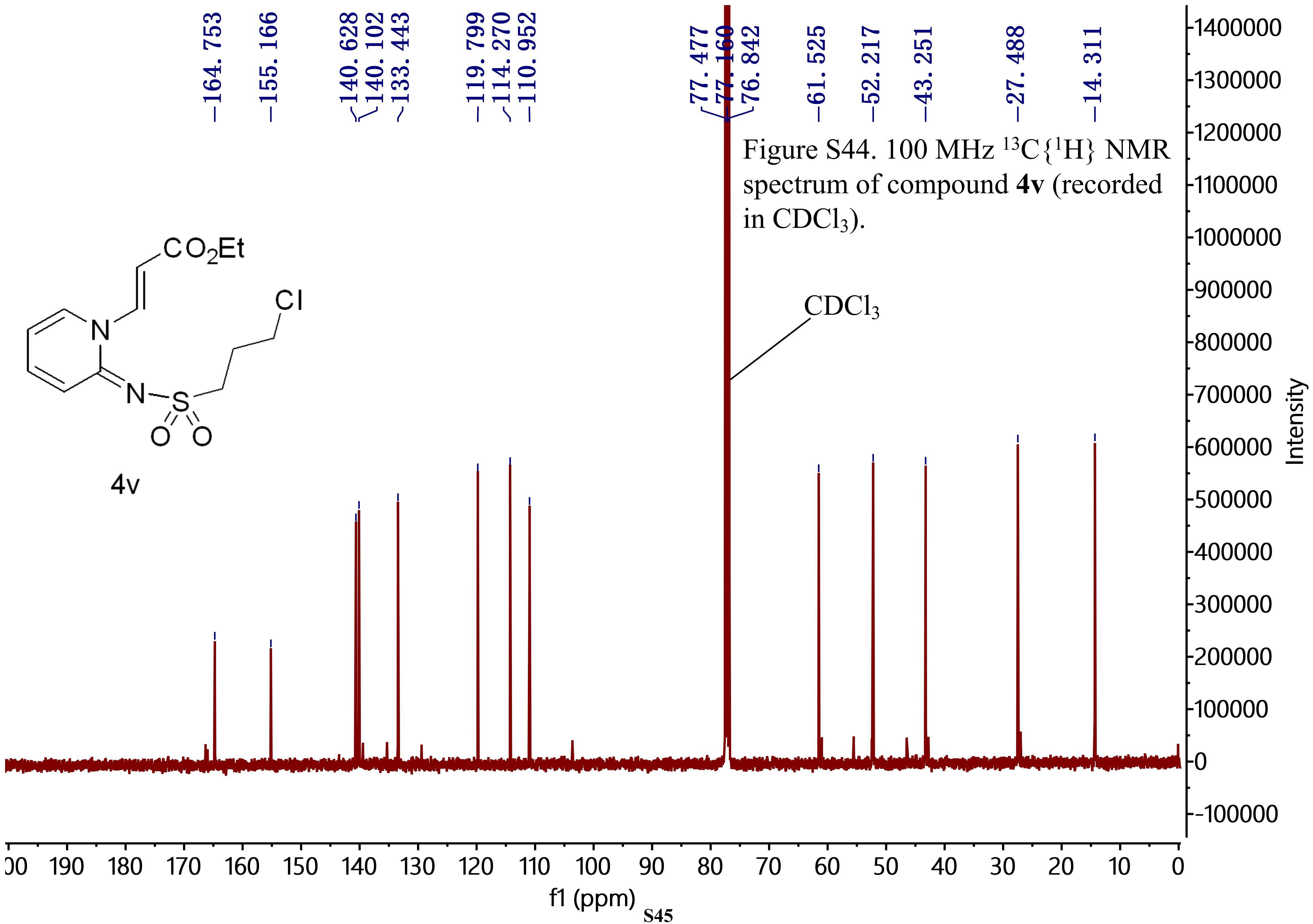

Figure S44. 100 MHz $^{13}C\{^1H\}$ NMR spectrum of compound **4v** (recorded in $CDCl_3$).

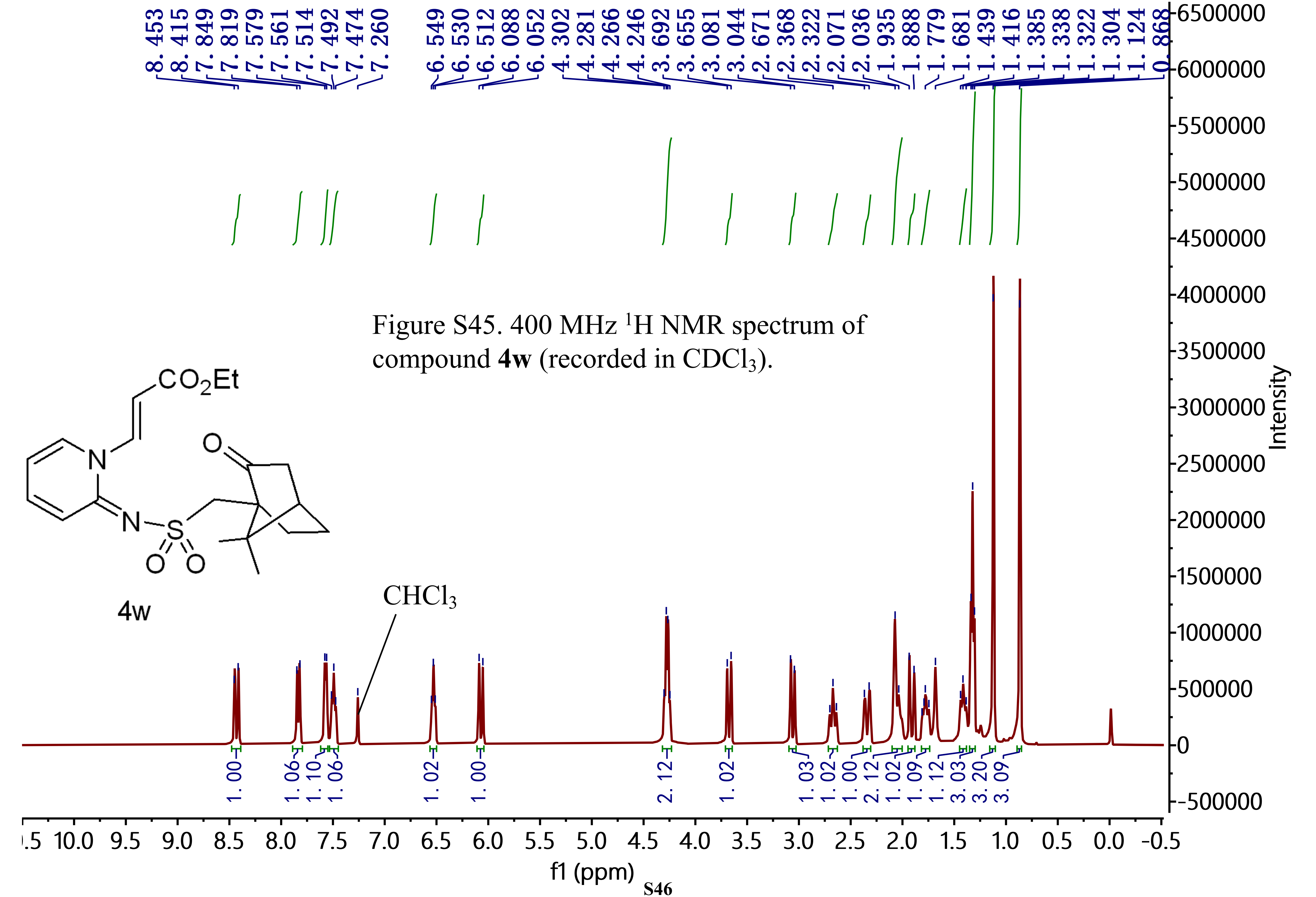

Figure S45. 400 MHz ^{1}H NMR spectrum of compound **4w** (recorded in $CDCl_3$).

Figure S46. 100 MHz $^{13}C\{^1H\}$ NMR spectrum of compound **4w** (recorded in $CDCl_3$).

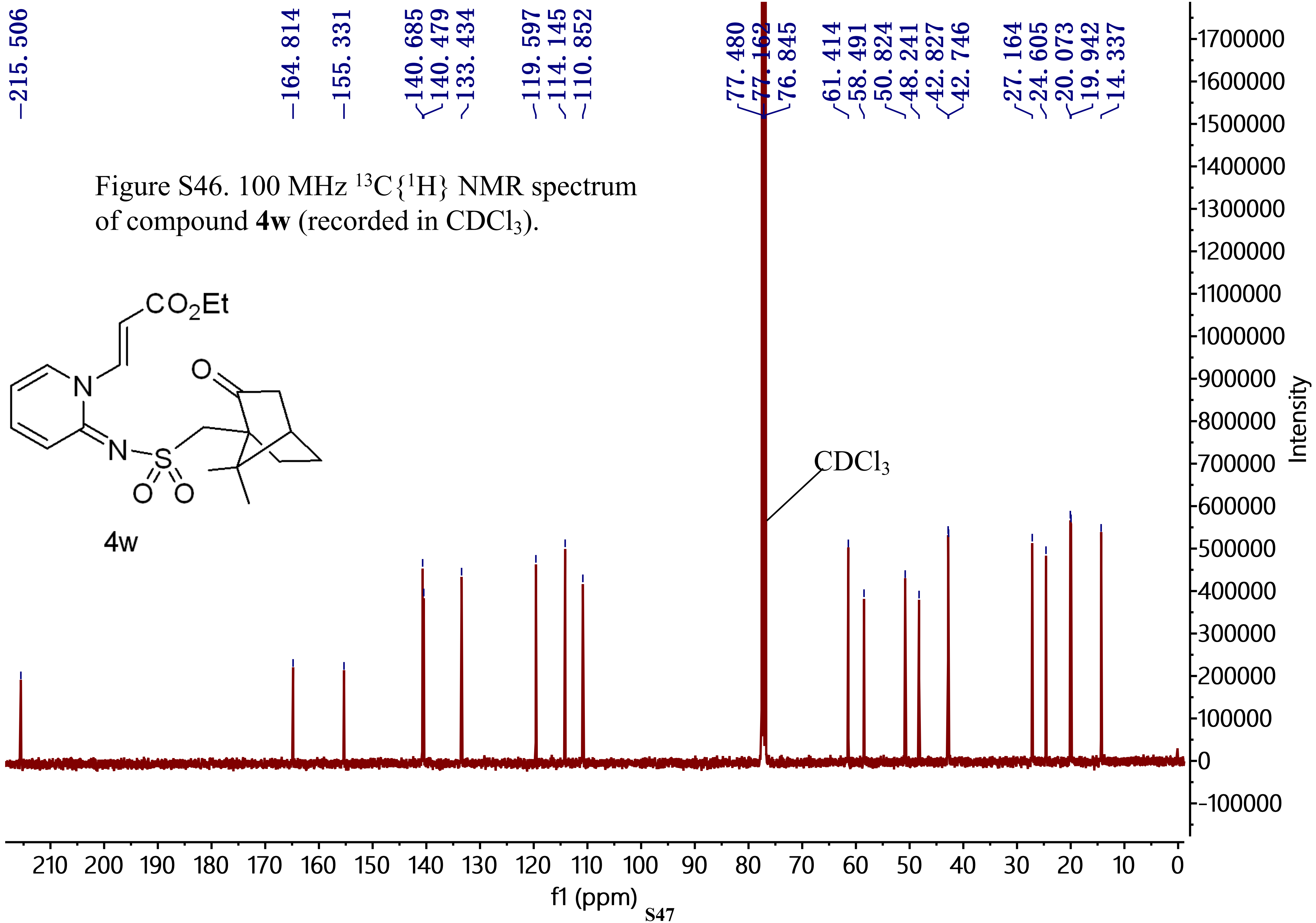

Supplement: Supplementary file 1 [file molecules-28-06511-s001.zip › molecules-2557374-supplementary.pdf]
